# Comparative analysis of genome tiling array data reveals many novel primate-specific functional RNAs in human

**DOI:** 10.1186/1471-2148-7-S1-S14

**Published:** 2007-02-08

**Authors:** Zhaolei Zhang, Andy Wing Chun Pang, Mark Gerstein

**Affiliations:** 1 Banting & Best Department of Medical Research, Donnelly CCBR, 160 College Street, University of Toronto, Toronto, ON M5S 3E1, Canada; 2 Department of Medical Genetics and Microbiology, University of Toronto, Toronto, ON M5S 3E1, Canada; 3 Department of Molecular Biophysics and Biochemistry (MBB), Yale University, New Haven, CT 06511, USA

## Abstract

**Background:**

Widespread transcription activities in the human genome were recently observed in high-resolution tiling array experiments, which revealed many novel transcripts that are outside of the boundaries of known protein or RNA genes. Termed as "TARs" (Transcriptionally Active Regions), these novel transcribed regions represent "dark matter" in the genome, and their origin and functionality need to be explained. Many of these transcripts are thought to code for novel proteins or non-protein-coding RNAs. We have applied an integrated bioinformatics approach to investigate the properties of these TARs, including cross-species conservation, and the ability to form stable secondary structures. The goal of this study is to identify a list of potential candidate sequences that are likely to code for functional non-protein-coding RNAs. We are particularly interested in the discovery of those functional RNA candidates that are primate-specific, i.e. those that do not have homologs in the mouse or dog genomes but in rhesus.

**Results:**

Using sequence conservation and the probability of forming stable secondary structures, we have identified ~300 possible candidates for primate-specific noncoding RNAs. We are currently in the process of sequencing the orthologous regions of these candidate sequences in several other primate species. We will then be able to apply a "phylogenetic shadowing" approach to analyze the functionality of these ncRNA candidates.

**Conclusion:**

The existence of potential primate-specific functional transcripts has demonstrated the limitation of previous genome comparison studies, which put too much emphasis on conservation between human and rodents. It also argues for the necessity of sequencing additional primate species to gain a better and more comprehensive understanding of the human genome.

## Background

### Whole genome tiling array experiments

The human genome is the blueprint that encodes most of the functional components in the human body: proteins and RNAs. With the completion of sequencing of the human genome, the focus of the genomic research is shifting to identifying all the functional units encoded within the genome. A new technology, the maskless oligonucleotide tiling array, has recently emerged as a powerful tool to interrogate transcription activities on the whole genomic scale and at unprecedented high resolution [1,2]. Using known genome sequence as blueprint, short oligonucleotides were synthesized to cover or "tile" each chromosome at regular intervals. Repetitive elements and regions of low complexity are usually avoided in such tiling experiments. Biological samples such as mRNAs or cDNAs are labelled with fluorescence and hybridized to the microarray spotted with the probes. Just like regular microarray experiments, the observed fluorescence intensities are interpreted as elevated transcription activity at specific genome locations. The tiling array experiments are most useful in verifying predicted exons and identify novel exons and other transcribed sequence elements.

A number of tiling array studies on the human and other genomes have been published since 2002 [3-6]. The major differences among these studies are the resolution of the tiles (length of the oligonucleotide probes and intervals between them), and the coverage of the genome. As of early 2006, the study by Bertone et al. (2005) is the only one that covers the entire human genome. These researchers designed ~51,000,000 probes of 36 mers, positioned at every 46 nucleotide interval on average, which cover ~1.5 GB of the non-repetitive genomic DNA sequence from both strands of the human genome [4]. The biological sample used in this study was fluorescence-labelled cDNA, reverse-transcribed from triple-selected polyadenylated RNAs [poly(A)+] from liver tissue. In total, these researchers identified ~17,000 transcriptionally active regions (termed as TARs) in the entire genome. There are strong correlations between the TARs and the known gene annotations or predictions, e.g. 64% of the genes annotated in RefSeq and 57% in Ensembl and 70% in UniGene were observed in this study [4].

### Widespread transcription activity in the human genome

The big surprise from the tiling array study is that transcription activities were observed in many genomic regions that do not overlap with known gene annotations. In fact, only about 40% of the TARs correspond to known exons, and a significant fraction of the TARs (6,656 or 38.7%) are more than 10 kb away from any known exons. Table 1 divides the TARs into groups according to their distance to the nearest genes that are on the same strand and also the opposite strand of the TAR. To estimate the enrichment or depletion of the TARs in the different regions, in Table 2 we break down the human genome into 25 categories in the same way as for the TARs and list the total length of these regions. Table 3 lists the density of the TARs in these regions, for instance the upper-left corner indicates that on average 574 base pairs per Mb in the Distal/Distal category has evidence of transcription as observed in the Bertone experiment. In contrast, on average 34,200 base pairs per Mb (3%) has evidence of transcription. It is likely that only a fraction of the human genes are transcribed in the liver cell line, thus transcription activity is not observed for all the annotated exons in the genome.

Such widespread transcription activities have also been observed in other human tiling array experiments as well [3-6]. There has not been a consensus opinion on the exact nature and origin of these TARs (or called "transfrags" as in [6]), however, it has been pointed out that many of these novel transcripts are not likely to code for proteins as they do not have open reading frames of longer than 300 nucleotides.

In addition to these microarray studies, widespread transcription activities outside of known human genes have also been observed in other types of experiments. Long serial analysis of gene expression experiments (LongSAGE) suggest that over 15,000 additional new exons exist in the human genome, and over half of these may be from new genes [7,8]. Ota et al. analyzed full-length human cDNA library and discovered ~5,000 novel non-coding cDNA transcripts [9]. A large number of noncoding transcripts has also been reported to exist in the mouse genome [10,11]. In addition to the mammalian genomes, large number of intergenic transcripts were also observed in plants and fruit fly [12-14]. Taking all these pieces of evidences together, it was estimated that over half of the human genome could potentially be transcribed [15], or at least 90% of the transcription activity in the genome is outside of well-characterized protein-coding exons [6].

### Possible functional roles of the novel transcripts

There have been many theories proposed to account for the origin, property, and possible functions of these novel transcripts. It was suggested that some of these TARs may be novel protein coding genes, novel RNA genes, anti-sense transcripts, alternative transcripts or just simply biological artefacts (please see [2] for a detailed discussion on the possible hypotheses). Because of the prevalence of such intergenic transcription activity (see above), it is unlikely that these novel transcripts are experimental artefact or false positives. It is also unlikely that any single mechanism can fully explain the observed novel transcripts, but perhaps the combination of explanations can account for the bulk of the observed novel transcripts. For example, it is possible that those TARs that are near the known genes could represent previously unidentified exons in the same gene structure, or represent alternative transcripts caused by alternative promoters. Depending on their degree of sequence conservation, some of the "distal" transcripts, i.e. those that are far away from known genes, are likely to be candidates for novel protein coding genes or noncoding RNAs. This notion has been suggested by many, including the researchers who conducted the tiling array experiments, as the most likely explanation for the bulk of the novel transcripts [9,11,16].

### Some of the TARs may be functional noncoding RNAs

Mammalian genomes contain many RNA genes that do not code for proteins; these are collectively called noncoding RNAs or ncRNAs [17,18]. The most well known noncoding RNAs include rRNA, tRNA, snoRNA, Xist and microRNAs (miRNAs). Table 4 lists some ncRNAs that were recently discovered and also implicated in human disorders. Some of these longer ncRNAs are sometimes referred to as mRNA-like ncRNAs (mlncRNAs) because they share properties with mRNAs such as splicing [19]. With the accumulating evidence on their prevalence and importance, ncRNAs have become increasingly appreciated as crucial components of cellular and organismal complexity, which prompts some to ponder whether we still live in an RNA world [20].

Many of the known ncRNAs in human and mouse were discovered accidentally or from large-scale cloning experiments. The novel transcripts found in the tiling array experiments offer a new resource to identify novel non-coding RNA transcripts. Kampa and colleagues have screened the "transfrags" from Chromosomes 21 and 22 and identified 193 novel ncRNA candidates; they were able to verify 126 or 65% of these ncRNAs by RT-PCR. Remarkably, this extrapolates to ~4200 ncRNAs in the entire human genome [16]. Several software tools have been developed to predict ncRNAs by computational approaches, especially on predicting miRNA, which have defined secondary structures. These programs mostly work by searching for conserved motifs, existence of secondary structure, cross-species conservation, or combination of above methods.

**Figure 1:**
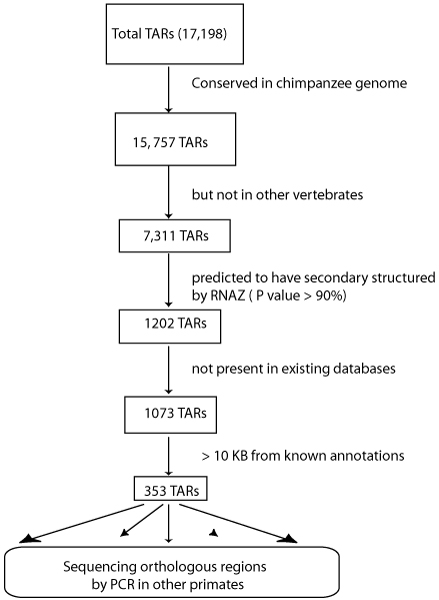
A flow chart of the analysis pipeline.

In this paper, we describe our bioinformatics analysis of these novel transcripts that were identified in the tiling array experiment [4]. We will investigate their sequence conservation in other species, their potential of forming stable secondary structures and ultimately the possibility that they could code for functional noncoding RNAs. Figure 1 is a flowchart outlining the basic analysis steps.

## Results

### Large numbers of novel transcripts are conserved in other vertebrates

We have BLASTed the TAR sequences against the genomic sequences of a number of fully or partially sequenced vertebrates, including mouse, rat, chimpanzee, chicken, dog, sea squirt, frog, and two kinds of pufferfish (Table 5). All these sequences were downloaded from Ensembl website, repetitive elements were removed by RepeatMasker [21].

Sequencing projects of two primates, Macaque (*Macaca mulatta*) and orangutan (*Pongo pygmaeus*), are currently under way. We downloaded the trace sequence files of these two primates and included them in the homology search as well. Because of the incompleteness of these genomes, the existence or absence of homologs in these libraries does not reflect the true level of conservation for each TAR. We also searched for TAR homologs in mammalian cDNA and EST libraries, including H-Invitational Database (H-InvDB) [22]), which contains 21,037 validated human full-length cDNA: mouse full-length cDNA library (FANTOM) [10,23]); human and mouse EST libraries from NCBI; and a macaque cDNA library from [24].

Table 5 shows that 69% of the TARs have EST matches, and 43% of the TARs have matches in the human cDNA library, which further validated that the bulk of the novel transcripts identified from tiling arrays are real transcripts instead of experimental artefacts. As expected, more TARs are conserved in the chimpanzee genome than in the rodent genomes (90% vs. 50%), which is in line with what would be expected for random genomic regions. This is obviously the result of closer evolutionary relationship between the two primate species, but it also implies that there must be many primate-specific transcripts that are shared between human and chimpanzee but not between human and rodents. A significant number of TARs are also conserved in chicken (21%) and pufferfish (16%).

### TARs in noncoding RNA databases

We also searched for homologs of the TARs in several sequence databases that contain known noncoding RNAs (Table 5, bottom). We found that there are ~2,637 non-protein-coding RNAs among the TARs, or about 15% of the entire novel transcripts, which also include 138 miRNAs. Note that some of these databases such as RNAdb also include hypothetical ncRNAs that are predicted from cDNA libraries.

In addition to number of homologs in single organisms, Table 6 further lists the number of TARs that are conserved in more than one species, i.e. with different conservation profiles. There are 4,806 transcripts (27%) that are only present in human and chimpanzee but not in any other genomes. Among these, 4,574 are not found in any ncRNA databases.

### Distal versus proximal TARs

Table 7 further divides these TARs according to their distance to the nearest annotated genes. It is intriguing to note that the "Distal/Distal" category has more TARs than any other category (highlighted by big bold fonts), even for those TARs that are only found in chimpanzee. It is likely that these are candidates for potential new protein genes or noncoding RNAs. It is also clear that the TARs that are far away from known genes tend to contain more primate-specific transcripts than TARs near genes (2036 versus 1209 or 27.8% versus 14.3%). This may be because the intergenic regions are less conserved between primate and rodents, which consequently could be the places where primate-specific transcripts are born.

### Many TARs are predicted to have stable secondary structures

It has been proposed that many of the transcripts identified in the tiling array or cloning experiments are novel non-protein-coding RNAs that have potential regulatory or catalytic functions. Okazaki and colleagues analyzed the mouse full-length cDNA library, and estimated 15,000 or about half of the library are non-protein-coding and functional RNA genes [10], but this number has been debated [25]. A more thorough computational study of the mouse transcriptom by Numata et al revealed a set of ~4,200 functional non-protein-coding RNA candidates [11]. Kampa and colleagues analyzed the tiling array data of human chromosome 21 and 22 [16]. They identified 193 novel RNA candidates and experimentally verified 126 of them. These researchers only used evidence of transcription in their predictions, which is powerful as demonstrated by the respectable 65% verification rate, but the false-positive rates can be further reduced if additional lines of evidence are incorporated. In this study, we utilize 2 lines of evidence: sequence conservation and RNA secondary structure, to make prediction on conserved novel RNA transcripts hidden in the human genome.

#### Sequence conservation

Functional elements in the genomes are presumably under selective pressure to maintain their sequence, therefore sequence conservation in other organisms are generally a good indication of functionality. However, we have to be cautious when applying such principle onto RNA sequences. It has been observed that except for structural RNAs such as rRNAs, noncoding RNA genes are in general less conserved than protein coding genes. This is particular true for regulatory RNAs [26], as some of the non-protein-coding RNAs, which have identical functions in human and mouse, do not show obvious sequence homologies. In addition, as pointed out earlier, too much reliance on conservation can overlook lineage-specific transcripts. Given the limitations of using sequence conservation alone in detecting ncRNAs, it is obvious that additional approaches are needed to address these concerns, such as the probability of forming stable secondary structure.

#### RNA secondary structure and thermodynamic stability

**Figure 2:**
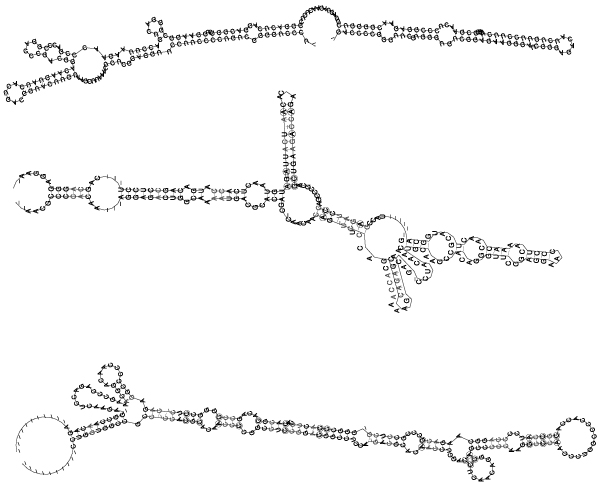
**Example of secondary structures of three candidates for novel primate-specific non-protein-coding RNAs as predicted by program RNAZ**. The genomic coordinates (NCBI build 34) of the three TARs are chromosome 2:10,415,689–10,415,908; chromosomes 11: 11,3423,510–113,423,729; and chromosome 7:56,614,161–56,614,382.

Functional ncRNAs usually form stable secondary structures, thus the potential of forming stable secondary structure are often considered as an indicator of functional RNAs [27]. We evaluated a number of existing tools and elected to use RNAZ in the analysis [28]. For every category of TARs that are listed in Table 7, we further filtered them by running RNA structure prediction program (RNAZ) on their sequences, the results are listed in Table 8. This filtering step reduced the number of ncRNA candidates by 6–7 folds; for example, only 1202 primate-specific TARs are predicted to have stable secondary structures, and only 1073 of them are novel sequences that do not have similar sequences in existing databases. We are more interested in the "Distal" TARs since they are at least 10 kb away from known genes and likely represent new ncRNA transcripts. There are 353 of these novel ncRNA candidates in the final set of candidates. Chromosomal coordinates and DNA sequences for these ncRNA candidates can be found in Additional Files 1, 2 and 3. Figure 2 shows the predicted structures of three possible ncRNAs. Please see Additional Files 1, 2 and 3 for more details.

It is interesting and encouraging that our analysis has discovered a large number of potential noncoding RNAs that only exist in the primates. Conventional genome annotation efforts often limit the cross-species comparison to human and mouse, such strategy likely have overlooked many lineage-specific protein or RNA genes. As we discuss below, special strategies are needed to uncover these lineage-specific sequences.

## Discussion

### Primate-specific noncoding RNAs in the human genome

It is important and fascinating to identify and characterize the genes that are responsible for the primate or human distinctiveness. In this paper, we discussed a bioinformatics analysis on the novel RNA transcripts discovered in our previous tiling array work [4]. We are interested to identify those functional novel transcripts that are primate-specific, i.e. they emerged only recently in the primate lineage and thus have no obvious sequence homologs in other mammalian genomes. This is a novel research area that has been largely overlooked, and it potentially will have great impact in the field of non-protein-coding RNAs, comparative genomics, and also medicine.

Most of the current efforts in detecting novel coding or noncoding transcripts require the transcript to be conserved in at least another mammalian genome, mostly in rodent genomes since they were the only available mammalian genomes until the chimpanzee draft genome was finished in 2005. Rodents and human last shared common ancestor at about 75–80 million years ago; their evolutionary distance from human is considered sufficiently distant to be able to separate conserved functional sequence that are under selective (purifying) pressure from those background neutral DNA [29,30]. A potential limitation of only using rodents as the yardstick in such comparative studies is that it overlooks those genes that have only emerged recently in the primate line-age, which likely determine primate-specific traits. Three-way comparisons between human-mouse-rat genomes have revealed 2302 rodent-specific exons, and similar number of human-specific genes [30-32]. These new genes were believed to have arisen through the following processes: (i) accelerated evolution in one lineage, (ii) arisen de novo from non-coding DNA, and (iii) derived from retroposition or recombinations [33]. Similarly, lineage-specific ncRNAs must also be present in either rodents or primates, which remain to be discovered.

### Comparison with other predictions

Pedersen and colleagues recently developed a computational method called EvoFold, which utilizes the algorithm of phylogenetic stochastic context-free grammar (Phylo-SCFG) to detect conserved structured RNAs in the genome [34]. These researchers first aligned the whole genome sequences of eight vertebrates (human, chimpanzee, mouse, rat, dog, chicken, zebra-fish, and puffer-fish), and applied the EvoFold program to derive 48,479 sequences in the human genome that are predicted to have secondary structures. These predicted sequences can be accessed at the UCSC genome browser.

We are interested to analyze the overlap between the Evofold predictions and the TARs. For each TAR, we identified the closest ncRNA candidate as predicted by Evofold. Surprisingly these two datasets have very little overlap: 548 TARs overlap with an Evofold prediction, and 624 TARs (including the overlapping ones) are within 100 bp of a nearest Evofold prediction. Among the 548 TARs that overlap with Evofold, only 16 were predicted by RNAZ to be noncoding RNAs with P-value greater than 0.5. The lack of overlap between TARs and the Evolfold predictions is not really surprising, as the latter only looked at the genomic regions that are conserved in eight vertebrate species.

### Phylogenetic Shadowing

As discussed above, the conventional phylogenetic and comparative methods have their limitations in identification of lineage-specific transcripts. An alternative approach, "phylogenetic shadowing", has been recently used in a number of studies and is likely to be very useful in this area. Phylogenetic shadowing is an alternative method to phylogenetic footprinting, which is a more commonly used comparative technique [35]. Both methods use comparative approach to identify functional elements hidden in a group of orthologous sequences, but they work in different ways and are most suited in different situations. Phylogenetic footprinting is most useful in searching for conserved elements that are present in organisms that are very distantly related. As at such great evolutionary distance, any conserved sequence would have been the result of selective pressure, therefore must be functionally important. In contrast, phylogenetic shadowing is best suited to study sequences from a group of closely related species. It analyzes patterns of sequence variations and mutations in a multiple sequence alignment, and separates the slowly evolving sites from the fast evolving sites. The sites that evolve slower are inferred as being under stronger selective pressure thus functionally important. In order to rigorously calculate the sequence variations without bias, phylogenetic relationships among the species is usually required. For closely related species, such phylogeny information is normally easy to obtain. Boffelli and Rubin were the first to employ phylogenetic shadowing, who used it on sequences from primates to discover regulatory elements and exon/intron boundaries [35]. Phylogenetic shadowing was recently used among a group of 9 primate species to identify conserved miRNA sequences [36].

### Future directions

We have initiated a sequencing project to obtain orthologous sequences for the 353 candidate transcripts from several related primate species. Experimental details and analysis will be reported in the future.

## Methods

### Searching for homologs in other genomes

For each TAR, we used Blastn to search for homologous sequences in another fully sequenced genome or sequence library. It is important to select the most optimal Blastn e-value threshold, so that we will not miss any real homologs, and also avoid too many false positive hits. To select the e-value cut-off, we did the following control experiments. We selected the experimentally verified human miRNA hairpin sequences from the mirRegistry database [37] as query sequences, and BLASTed them against the mouse genome. We also included some negative sequences into the query set. All of these known human miRNAs have homologs in the mouse genome, so the resulted e-values from this Blastn search should be the optimal cutoff for selecting homologs in a different genome. The results confirmed that e-value = 0.01 is sufficient to identify the homologs and separate the real homologs from negative controls.

### Predicting secondary structure in RNAs

Programs such as MiRscan, miRseeker and ProMiR are dedicated to search for miRNAs [38-40] and programs such as RNAZ, RNAFOLD, Mfold, ddbRNA, RANDFOLD, MSARI, QRNA, FOLDALIGN were written to detect stable RNA secondary structures [28,41-46]. A track (EvoFold) was also implemented into the UCSC Genome Browser, which indicates the potential of forming secondary structures for any give genomics locus. A number of databases have been created to collect and categorize these ncRNA sequences, which include Rfam [47], NONCODE [48], microRNA Register [37], and RNAdb [49].

Among these RNA structure prediction tools, the RNAZ program has been evaluated as the most effective [50], and was used as the primary prediction tool [28]. The effectiveness of the RNAZ program comes from its unique approach in combining the predicted thermodynamic stability with the structure and sequence conservation index [28]. The program has been tested on positive and negative control sequences. At the P value cutoff at 0.9, the program has the sensitivity of 75% and specificity of 98% [28]. We are currently also testing other prediction software such as QRNA, we will compare these two prediction results and investigate the possibility of using the intersection of the two predictions.

## Authors' contributions

ZZ conducted most of the computational analysis; AP is responsible for evaluating and running the RNAZ program. MBG was responsible for the initiation of the original tiling array experiment and providing the data.

## Supplementary Material

Additional file 1 chromosomal coordinates of the final 353 candidate TAR sequences based on NCBI build 34 Click here for file

Additional file 2 DNA sequence of these candidate sequences Click here for file

Additional file 3 UCSC genome browser screen shots of these 3 candidate sequences as shown in Figure 2. These screenshots show that these sequences are either absent or less conserved in the mouse and rat genomes, thus are primate-specific. Click here for file

## Figures and Tables

**Table 1:** Distribution of transcriptionally active regions (TARs), categorized by their distances from the nearest gene annotations

	Annotation on the opposite strand						
	
		Distal	10 kb	1 kb	exon	intron	Total
	
Annotation on the same strand as TAR^4^	^1 ^Distal	3,695 (21.5%)	537 (3.1%)	149 (0.9%)	861 (5.0%)	1,414 (8.2%)	6,656 (38.7%)
	^2^10 kb	799 (4.6%)	224 (1.3%)	59 (0.3%)	265 (1.5%)	146 (0.8%)	1,493 (8.7%)
	^3^1 kb	347 (2.0%)	92 (0.5%)	32 (0.2%)	52 (0.3%)	20 (0.1%)	543 (3.2%)
	exon	4,637 (27.0%)	1,454 (8.4%)	345 (2.0%)	120 (0.7%)	172 (1.0%)	6,728 (39.1%)
	intron	1,554 (9.0%)	137 (0.8%)	24 (0.1%)	35 (0.2%)	28 (0.2%)	1,778 (10.3%)
							
	Total:	11,032 (64.1%)	2,444 (14.2%)	609 (3.5%)	1,333 (7.8%)	1,780 (10.4%)	17,198

^1^Distal: distance from nearest annotated exon > 10 kb

^2^10 kb: distance from nearest annotated exon is less than 10 kb but longer than 1 kb

^3^1 kb: distance from nearest annotated exon is less than 1 kb but do not overlap with exons

^4 ^Genome annotation was based on NCBI assembly 34 downloaded from Ensembl.

**Table 2:** Distribution of human genomic regions, categorized by annotations on both strands (Mb = megabases)

	Annotation on the antisense strand (Mb)						
	
		Distal	10 kb	1 kb	exon	intron	Total
	
Annotation on the sense strand ^1^	Distal	1,655 (54.%)	130 (4.2%)	17.6 (0.6%)	37 (1.2%)	438 (14.2%)	2,279 (74%)
	10 kb	133 (4.3%)	26 (0.9%)	49 (0.1%)	7 (0.2%)	34 (1.1%)	206 (7%)
	1 kb	17 (0.6%)	4 (0.1%)	1 (0.03%)	1 (0.04%)	2.8 (0.1%)	27 (0.9%)
	exon	44 (1.4%)	7.6 (0.25%)	1 (0.04%)	1.1 (0.04%)	3.8 (0.12%)	58 (1.9%)
	intron	450 (14.6%)	35 (1.2 %)	3.0 (0.1%)	4 (0.1%)	12 (0.4%)	505 (16%)
							
	Total:	2301 (75%)	204 (6.7%)	27 (0.9%)	50.7 (1.6%)	491 (16%)	3,076

^1^Annotation is the same as in Table 1.

**Table 3:** Total length and density of TARs in different types of genomic regions^1^

	Annotation on the antisense strand						
	
		Distal	10 kb	1 kb	exon	intron	Total
	
Annotation on the sense strand^2^	Distal	950,963 (574)	137,168 (1,048)	38,856 (2,202)	223,699 (6,012)	364,308 (831)	1,715,000 (753)
	10 kb	212,945 (1,596)	61,991 (2,334)	15,186 (3,584)	69,413 (9,683)	37,434 (1,077)	396,969 (1,930)
	1 kb	97,974 (5,454)	24,062 (5631)	8,793 (8,647)	14,475 (13,391)	4,960 (1,751)	150,264 (5,570)
	exon	1,370,991 (30,952)	422,364 (55,666)	103,263 (90,951)	33,079 (29,331)	53,984 (1,4043)	1,983,681 (34,200)
	intron	398,063 (884)	34,368 (970)	6,230 (2,104)	8,752 (2,107)	7,059 (586)	454,472 (900)
							
	Total:	3,030,936 (1,320)	679,953 (3,330)	172,328 (6,380)	349,418 (6,890)	467,745 (953)	

^1^Numbers in the brackets are the normalized densities of the TARs, i.e. number of transcribed nucleotides per megabase of genomic DNA

^2^Annotation is the same as in Table 1(A)

**Table 4:** Noncoding RNAs that are known to have medical implications.

**RNA name**	**disorder**	**Reference**
H19 RNA	Tumor suppressor	[51]
BIC	Hodgkin lymphoma	[52, 53]
DLEU2,	lymphocytic leukaemia	[54];
NCRMS	rhabdomyosarcoma	[55]
7H4	postnatal development	[56],
NRSE/REST	found in adult neural stem cells	[57].

**Table 5:** BLAST matches in the other genomes and databases (E-value < 0.01)

**Genome or database**	**Number of hits**	**fraction**
Human EST library	12,021	70%
Human cDNA library	7,546	44%
Chimpanzee (*Pan troglodytes*)	15,757	92%
Macaque cDNA library	5,955	35%
Mouse genome	8,011	47%
Mouse EST library	7,546	44%
Rat genome	7,691	45%
* Rodents: mouse ∪ rat	9,184	53%
Dog (*Canis familiaris*,) partial genome	7,924	46%
Chicken (*Gallus gallus*)	3,600	21%
Frog (*Xenopus tropicalis*)	2,279	13%
Japanese pufferfish (*Takifugu rubripes*)	2,254	13%
Green spotted pufferfish (*Tetraodon nigroviridis*)	2,069	12%
** Pufferfish: *Takifugu rubripes ∪ Tetraodon nigroviridis*	2867	17%
Sea squirt (*Ciona intestinalis)*	607	4%
		
**Noncoding RNA Databases**		
Rfam	252	1%
RNAdb	1,995	12%
mirBase	138	1%
NONCODE	379	2%
*** Rfam ∪ RNAdb ∪ mirBase ∪ NONCODE	2,637	15%
		
**Total number of TARs reported by Bertone et al.**	**17,198**	

* number of TARs that have hits in either mouse or rat genome, The symbol ∪ represents the union of two sets

** number of TARs that have hits in either species of pufferfish

*** number of TARs that have hits in either of the noncoding RNA database

**Table 6:** Conservation profiles of TARs among vertebrates

Conservation profile *	**# of hits**	**fraction**
Human ∩ Chimp	15,757	92%
Human ∩ Chimp ∩ rodents	8,828	51%
Human ∩ Chimp ∩ rodents ∩ dog	5,988	35%
Human ∩ Chimp ∩ rodents ∩ dog ∩ chicken	2,435	14%
Human ∩ Chimp ∩ rodents ∩ dog ∩ chicken ∩ pufferfish	1,244	7%
Human ∩ Chimp ∩ rodents ∩ dog ∩ chicken ∩ pufferfish ∩ sea squirt	276	2%
		
Human ∩ macaque	5,955	35%
Human ∩ macaque ∩ chimp	5,815	34%
		
Human ∩ Rodent	8,776	51%
Human ∩ Rodent ∩ dog	6,166	36%
Human ∩ Rodents ∩ dog ∩ chicken	2,493	14%
Human ∩ Rodent ∩ dog ∩ chicken ∩ pufferfish	1,270	7%
Human ∩ Rodent ∩ dog ∩ chicken ∩ pufferfish ∩ sea squirt	276	2%
		
Num. of TARs in human AND chimp, but NOT in rodents	6,929	40%
Num. of TARs in human AND chimp, but NOT in any other vertebrates	4,806	28%
		
Num. of TARs in human and chimp, but NOT in rodents, and NOT in databases (Rfam, RNAdb, mirBase, NONCODE)	6,132	36%
Num. of TARs in human and chimp, but NOT in any other genomes, and NOT in databases (Rfam, RNAdb, mirBase, NONCODE)	4,574	27%
		
**Total # of TARs**	**17,198**	

* ∪ represents the union of two or more sets

**Table 7:** Conservation of TARs in chimpanzee and rodents, categorized by their distance to known genes on both strands

	**Same strand**	**Opposite strand**				
		Distal	10 kb	1 kb	exon	intron
	
Found in human AND chimp (15,757)	Distal	**3245 (20.6%)**	**469 (3.0%)**	**130 (0.8%)**	**826 (5.2%)**	**1233 (7.8%)**
	10 kb	724 (4.6%)	197 (1.3%)	56 (0.4%)	232 (1.5%)	123 (0.8%)
	1 kb	327 (2.1%)	87 (0.6%)	30 (0.2%)	46 (0.3%)	18 (0.1%)
	exon	4492 (28.5%)	1368 (8.7%)	323 (2.0%)	116 (0.7%)	165 (1.0%)
	intron	1358 (8.6%)	115 (0.7%)	21 (0.1%)	31 (0.2%)	25 (0.2%)

		Distal	10 kb	1 kb	Exon	Intron
	
Found in human AND mouse (8,776)	Distal	**1256 (14.3%)**	**155 (1.8%)**	**51 (0.6%)**	**668 (7.6%)**	**402 (4.6%)**
	10 kb	340 (3.9%)	79 (0.9%)	20 (0.2%)	198 (2.3%)	33 (0.4%)
	1 kb	136 (1.5%)	42 (0.5%)	9 (0.1%)	25 (0.3%)	10 (0.1%)
	exon	3398 (38.7%)	1028 (11.7%)	217 (2.5%)	96 (1.1%)	125 (1.4%)
	intron	419 (4.8%)	29 (0.3%)	6 (0.1%)	24 (0.3%)	10 (0.1%)

		Distal	10 kb	1 kb	Exon	Intron
	
Found in human AND chimp AND rodents (**8,446**)	Distal	**1209 (14.3%)**	**146 (1.7%)**	**46 (0.5%)**	**649 (7.7%)**	**382 (4.5%)**
	10 kb	330 (3.9%)	75 (0.9%)	19 (0.2%)	180 (2.1%)	30 (0.4%)
	1 kb	133 (1.6%)	42 (0.5%)	8 (0.1%)	21 (0.2%)	10 (0.1%)
	exon	3306 (39.1%)	983 (11.6%)	206 (2.4%)	93 (1.1%)	121 (1.4%)
	intron	393 (4.7%)	26 (0.3%)	6 (0.1%)	22 (0.3%)	10 (0.1%)

		Distal	10 kb	1 kb	Exon	Intron
	
Found in human AND in chimp NOT in rodents (**7,311**)	Distal	**2036 (27.8%)**	**323 (4.4%)**	**84 (1.1%)**	**177 (2.4%)**	**851 (11.6%)**
	10 kb	394 (5.4%)	122 (1.7%)	37 (0.5%)	52 (0.7%)	93 (1.3%)
	1 kb	194 (2.7%)	45 (0.6%)	22 (0.3%)	25 (0.3%)	8 (0.1%)
	exon	1186 (16.2%)	385 (5.3%)	117 (1.6%)	23 (0.3%)	44 (0.6%)
	intron	965 (13.2%)	89 (1.2%)	15 (0.2%)	9 (0.1%)	15 (0.2%)

**Table 8:** Predictions of RNA secondary structures by RNAZ (p-value > 90%)

		Distal	10 kb	1 kb	exon	intron
	
Found in human AND chimp (1436)	Distal	418 (29.1%)	64(4.5%)	17(1.2%)	62(4.3%)	184 (12.8%)
	10 kb	70 (4.9%)	19(1.3%)	3(0.2%)	19(1.3%)	24(1.7%)
	1 kb	30 (2.1%)	8(0.6%)	4(0.3%)	3(0.2%)	1(0.1%)
	exon	164 (11.4%)	61(4.2%)	15(1.0%)	6(0.4%)	6(0.4%)
	intron	226(15.7%)	20(1.4%)	3(0.2%)	5(0.3%)	4(0.3%)
		Distal	10 kb	1 kb	Exon	Intron
	
Found in human AND mouse (241)	Distal	32(13.3%)	2(0.8%)	2(0.8%)	39(16.2%)	11(4.6%)
	10 kb	4(1.7%)	0(0.0%)	0(0.0%)	15(6.2%)	0(0.0%)
	1 kb	2(0.8%)	1(0.4%)	1(0.4%)	2(0.8%)	0(0.0%)
	exon	71(29.5%)	32(13.3%)	5(2.1%)	2(0.8%)	3(1.2%)
	intron	9(3.7%)	3(1.2%)	1(0.4%)	3(1.2%)	1(0.4%)

		Distal	10 kb	1 kb	Exon	Intron
	
Found in human AND chimp AND rodents (234)	Distal	31(13.2%)	2(0.9%)	2(0.9%)	38(16.2%)	10(4.3%)
	10 kb	4(1.7%)	0(0.0%)	0(0.0%)	14(6.0%)	0(0.0%)
	1 kb	2(0.9%)	1(0.4%)	1(0.4%)	1(0.4%)	0(0.0%)
	exon	70(29.9%)	32(13.7%)	5(2.1%)	2(0.9%)	3(1.3%)
	intron	9(3.8%)	2(0.9%)	1(0.4%)	3(1.3%)	1(0.4%)

		Distal	10 kb	1 kb	exon	Intron
	
Found in human AND in chimp NOT in rodents (1202)	Distal	387(32.2%)	62(5.2%)	15(1.2%)	24(2.0%)	174 (14.5%)
	10 kb	66(5.5%)	19(1.6%)	3(0.2%)	5(0.4%)	24(2.0%)
	1 kb	28(2.3%)	7(0.6%)	3(0.2%)	2(0.2%)	1(0.1%)
	exon	94(7.8%)	29(2.4%)	10(0.8%)	4(0.3%)	3(0.2%)
	intron	217(18.1%)	18(1.5%)	2(0.2%)	2(0.2%)	3(0.2%)

		Distal	10 kb	1 kb	exon	Intron
	
Found in human AND in chimp NOT in rodents, and NOT present in databases (1073)	Distal	**353(33.1%)**	56(5.2%)	13(1.2%)	22(2.1%)	151 (14.1%)
	10 kb	57(5.3%)	15(1.4%)	2(0.2%)	5(0.5%)	20(1.9%)
	1 kb	24(2.2%)	7(0.7%)	3(0.3%)	1(0.1%)	1(0.1%)
	exon	83(7.7%)	24(2.2%)	9(0.8%)	2(0.2%)	3(0.3%)
	intron	196 (18.3%)	17(1.6%)	2(0.2%)	2(0.2%)	3(0.3%)
