# Supplementary material for: Comparative analysis of genome tiling array data reveals many novel primate-specific functional RNAs in human
Source: BMC Evol Biol. 2007 Feb 8;7(Suppl 1):S14. doi: 10.1186/1471-2148-7-S1-S14 (PMC1796608; doi:10.1186/1471-2148-7-S1-S14)
Supplement: Additional file 1 — chromosomal coordinates of the final 353 candidate TAR sequences based on NCBI build 34 [file 1471-2148-7-S1-S14-S1.doc]

##TAR_id annotation_sense annnotation_antisense start_build34 end_build34 strand

chr1(-):109913398-109913617 Distal Distal 1 110345560 110345779 -

chr1(-):223812426-223812670 Distal Distal 1 225048455 225048699 -

chr1(-):224900444-224900663 Distal Distal 1 226144748 226144967 -

chr1(-):240661950-240662261 Distal Distal 1 242344378 242344689 -

chr1(+):219559879-219560190 Distal Distal 1 220679555 220679866 +

chr1(-):223825600-223825845 Distal Distal 1 225061629 225061874 -

chr1(-):242487143-242487434 Distal Distal 1 244197472 244197763 -

chr1(-):29870130-29870395 Distal Distal 1 30388015 30388280 -

chr1(+):223825148-223825367 Distal Distal 1 225061177 225061396 +

chr1(-):89797378-89797597 Distal Distal 1 90089443 90089662 -

chr1(-):196681122-196681350 Distal Distal 1 198510992 198511220 -

chr1(-):240669261-240669531 Distal Distal 1 242351689 242351959 -

chr1(+):19152997-19153216 Distal Distal 1 19587325 19587544 +

chr1(-):222045540-222045805 Distal Distal 1 223202962 223203227 -

chr1(-):46826901-46827120 Distal Distal 1 47307764 47307983 -

chr2(-):173349489-173349754 Distal Distal 2 174890883 174891148 -

chr2(-):908059-908370 Distal Distal 2 908060 908371 -

chr2(-):217195840-217196059 Distal Distal 2 218677004 218677223 -

chr2(-):43466979-43467198 Distal Distal 2 43272770 43272989 -

chr2(+):43257560-43257782 Distal Distal 2 43063351 43063573 +

chr2(-):127290009-127290277 Distal Distal 2 129459021 129459289 -

chr2(-):216580178-216580397 Distal Distal 2 218061342 218061561 -

chr2(-):15962542-15962784 Distal Distal 2 15933105 15933347 -

chr2(-):118994004-118994223 Distal Distal 2 121168395 121168614 -

chr2(+):129987126-129987462 Distal Distal 2 131135903 131136239 +

chr2(+):18604689-18604908 Distal Distal 2 18654856 18655075 +

chr2(+):119459480-119459745 Distal Distal 2 121633871 121634136 +

chr2(-):10445305-10445524 Distal Distal 2 10415689 10415908 -

chr2(-):6176618-6176837 Distal Distal 2 6146978 6147197 -

chr2(-):217608918-217609206 Distal Distal 2 219090083 219090371 -

chr2(-):217580701-217580932 Distal Distal 2 219061866 219062097 -

chr2(-):108392031-108392296 Distal Distal 2 109535234 109535499 -

chr2(+):127202283-127202502 Distal Distal 2 129371295 129371514 +

chr2(-):820100-820350 Distal Distal 2 820101 820351 -

chr2(-):125344276-125344495 Distal Distal 2 127511146 127511365 -

chr2(+):10445351-10445570 Distal Distal 2 10415735 10415954 +

chr2(-):2234328-2234547 Distal Distal 2 2141153 2141372 -

chr2(+):127290962-127291181 Distal Distal 2 129459974 129460193 +

chr2(+):130877035-130877254 Distal Distal 2 132800156 132800375 +

chr2(+):217620591-217620856 Distal Distal 2 219101756 219102021 +

chr2(+):1876547-1876766 Distal Distal 2 1783372 1783591 +

chr2(+):15988465-15988684 Distal Distal 2 15959084 15959303 +

chr2(-):127130799-127131018 Distal Distal 2 129299811 129300030 -

chr2(-):241019276-241019495 Distal Distal 2 241622669 241622888 -

chr2(-):108426989-108427208 Distal Distal 2 109570192 109570411 -

chr2(-):129483425-129483690 Distal Distal 2 132171439 132171704 -

chr2(+):20807867-20808132 Distal Distal 2 20724540 20724805 +

chr2(-):118403040-118403276 Distal Distal 2 120577431 120577667 -

chr2(-):203405997-203406216 Distal Distal 2 204883924 204884143 -

chr2(-):72528946-72529165 Distal Distal 2 72292674 72292893 -

chr2(-):119441829-119442048 Distal Distal 2 121616220 121616439 -

chr2(+):10445305-10445524 Distal Distal 2 10415689 10415908 +

chr2(-):217599433-217599744 Distal Distal 2 219080598 219080909 -

chr2(-):1916578-1916797 Distal Distal 2 1823403 1823622 -

chr2(-):70427387-70427606 Distal Distal 2 70191095 70191314 -

chr2(-):127028929-127029148 Distal Distal 2 129197941 129198160 -

chr2(-):109071690-109071909 Distal Distal 2 110543834 110544053 -

chr2(-):97841928-97842219 Distal Distal 2 99010599 99010890 -

chr2(+):6673364-6673583 Distal Distal 2 6643728 6643947 +

chr2(-):95192208-95192427 Distal Distal 2 96222828 96223047 -

chr2(+):95192116-95192473 Distal Distal 2 96222736 96223093 +

chr2(-):23826104-23826323 Distal Distal 2 23708051 23708270 -

chr2(-):173538443-173538708 Distal Distal 2 175079839 175080104 -

chr2(+):6126181-6126400 Distal Distal 2 6096541 6096760 +

chr2(-):127129210-127129429 Distal Distal 2 129298222 129298441 -

chr2(-):767947-768166 Distal Distal 2 767948 768167 -

chr2(-):130619433-130619697 Distal Distal 2 132650168 132650432 -

chr2(+):23834014-23834233 Distal Distal 2 23715961 23716180 +

chr2(-):177729844-177730063 Distal Distal 2 179261250 179261469 -

chr3(-):127099640-127100089 Distal Distal 3 128389525 128389974 -

chr3(-):135339385-135339742 Distal Distal 3 135917564 135917921 -

chr3(-):49585162-49585427 Distal Distal 3 50530433 50530698 -

chr3(-):127076540-127076759 Distal Distal 3 128366425 128366644 -

chr3(+):195011646-195011865 Distal Distal 3 195071184 195071403 +

chr3(-):127414303-127414568 Distal Distal 3 128583874 128584139 -

chr3(-):135384914-135385133 Distal Distal 3 135963093 135963312 -

chr3(+):13527447-13527666 Distal Distal 3 13562571 13562790 +

chr3(-):135410184-135410403 Distal Distal 3 135988363 135988582 -

chr3(-):127124049-127124406 Distal Distal 3 128413934 128414291 -

chr3(-):128343191-128343454 Distal Distal 3 129467208 129467471 -

chr3(-):126912803-126913068 Distal Distal 3 128202688 128202953 -

chr3(-):126318846-126319178 Distal Distal 3 127608723 127609055 -

chr3(-):127013144-127013363 Distal Distal 3 128303029 128303248 -

chr3(-):11072823-11073073 Distal Distal 3 11107955 11108205 -

chr3(-):99134374-99134593 Distal Distal 3 101176094 101176313 -

chr3(+):126061474-126061783 Distal Distal 3 127351347 127351656 +

chr3(-):142208287-142208506 Distal Distal 3 142700403 142700622 -

chr3(-):12935424-12935735 Distal Distal 3 12970548 12970859 -

chr3(-):128799136-128799354 Distal Distal 3 129738426 129738644 -

chr3(+):14340501-14340788 Distal Distal 3 14375625 14375912 +

chr3(+):126961347-126961566 Distal Distal 3 128251232 128251451 +

chr3(-):126834290-126834601 Distal Distal 3 128124175 128124486 -

chr3(-):14345332-14345551 Distal Distal 3 14380456 14380675 -

chr3(+):133304544-133304761 Distal Distal 3 133880566 133880783 +

chr3(+):13678900-13679309 Distal Distal 3 13714024 13714433 +

chr3(-):126856052-126856271 Distal Distal 3 128145937 128146156 -

chr3(+):13041820-13042039 Distal Distal 3 13076944 13077163 +

chr3(+):126840687-126840906 Distal Distal 3 128130572 128130791 +

chr3(+):106655246-106655465 Distal Distal 3 108448371 108448590 +

chr3(-):126885841-126886130 Distal Distal 3 128175726 128176015 -

chr3(-):138923489-138923754 Distal Distal 3 139452807 139453072 -

chr3(-):127405633-127405852 Distal Distal 3 128575204 128575423 -

chr3(-):135384822-135385041 Distal Distal 3 135963001 135963220 -

chr3(+):13679090-13679309 Distal Distal 3 13714214 13714433 +

chr3(+):126889548-126889767 Distal Distal 3 128179433 128179652 +

chr3(+):99134374-99134593 Distal Distal 3 101176094 101176313 +

chr3(-):126930829-126931048 Distal Distal 3 128220714 128220933 -

chr4(+):7869215-7869489 Distal Distal 4 7812267 7812541 +

chr4(-):154308612-154308847 Distal Distal 4 154497657 154497892 -

chr4(-):44977382-44977625 Distal Distal 4 44581392 44581635 -

chr4(-):154394328-154394547 Distal Distal 4 154583373 154583592 -

chr4(-):155320501-155320766 Distal Distal 4 155509554 155509819 -

chr4(-):26053629-26053848 Distal Distal 4 25704373 25704592 -

chr4(-):74833071-74833290 Distal Distal 4 75059691 75059910 -

chr4(-):186615540-186615779 Distal Distal 4 186631554 186631793 -

chr4(-):27209272-27209491 Distal Distal 4 26801971 26802190 -

chr4(-):153533732-153533951 Distal Distal 4 153722769 153722988 -

chr4(-):191627340-191627560 Distal Distal 4 191674431 191674651 -

chr4(+):184944120-184944367 Distal Distal 4 184960149 184960396 +

chr4(-):173280383-173280602 Distal Distal 4 173547013 173547232 -

chr4(-):184464583-184464832 Distal Distal 4 184480609 184480858 -

chr4(-):71626199-71626418 Distal Distal 4 23192729 23192948 -

chr4(-):109659171-109659390 Distal Distal 4 109796911 109797130 -

chr4(+):134668651-134668870 Distal Distal 4 134824731 134824950 +

chr4(-):23508651-23508870 Distal Distal 4 23176146 23176365 -

chr4(+):601209-601428 Distal Distal 4 596990 597209 +

chr4(-):184944801-184945027 Distal Distal 4 184960830 184961056 -

chr4(+):7034113-7034332 Distal Distal 4 6976645 6976864 +

chr4(+):188674989-188675392 Distal Distal 4 188690957 188691360 +

chr4(-):153424967-153425186 Distal Distal 4 153614004 153614223 -

chr4(-):111483395-111483706 Distal Distal 4 111671888 111672199 -

chr4(-):154421962-154422212 Distal Distal 4 154611007 154611257 -

chr4(-):151364564-151364829 Distal Distal 4 151567103 151567368 -

chr4(-):184420892-184421249 Distal Distal 4 184436308 184436665 -

chr4(-):100216201-100216466 Distal Distal 4 100364354 100364619 -

chr4(-):52991721-52991940 Distal Distal 4 52926845 52927064 -

chr4(-):185486945-185487210 Distal Distal 4 185502959 185503224 -

chr5(+):69936089-69936308 Distal Distal 5 68386529 68386748 +

chr5(+):10296587-10296806 Distal Distal 5 10179585 10179804 +

chr5(+):172707906-172708125 Distal Distal 5 171986987 171987206 +

chr5(+):1662698-1662917 Distal Distal 5 1577183 1577402 +

chr5(+):153935548-153935767 Distal Distal 5 153368397 153368616 +

chr5(-):142002064-142002329 Distal Distal 5 141447613 141447878 -

chr5(-):149416080-149416324 Distal Distal 5 148850149 148850393 -

chr5(+):179768884-179769130 Distal Distal 5 178979253 178979499 +

chr5(+):14288122-14288341 Distal Distal 5 14059688 14059907 +

chr5(-):139721520-139721739 Distal Distal 5 139167087 139167306 -

chr5(-):1042526-1042816 Distal Distal 5 1015614 1015904 -

chr5(+):107245710-107245929 Distal Distal 5 106876067 106876286 +

chr5(+):170754203-170754422 Distal Distal 5 170031736 170031955 +

chr5(-):136399129-136399348 Distal Distal 5 135812150 135812369 -

chr5(-):1087848-1088141 Distal Distal 5 1191966 1192259 -

chr6(-):169875726-169875945 Distal Distal 6 170016626 170016845 -

chr6(-):82983439-82983657 Distal Distal 6 83012336 83012554 -

chr6(-):137984152-137984371 Distal Distal 6 138117604 138117823 -

chr6(+):163316236-163316455 Distal Distal 6 163351961 163352180 +

chr6(+):145547276-145547495 Distal Distal 6 145680728 145680947 +

chr6(-):34140515-34140826 Distal Distal 6 34190955 34191266 -

chr6(-):95038414-95038633 Distal Distal 6 95067311 95067530 -

chr6(-):89222971-89223190 Distal Distal 6 89251868 89252087 -

chr6(-):38131795-38132014 Distal Distal 6 38182236 38182455 -

chr6(+):134282733-134282951 Distal Distal 6 134416185 134416403 +

chr6(-):38157430-38157658 Distal Distal 6 38207871 38208099 -

chr6(+):167801061-167801305 Distal Distal 6 167836786 167837030 +

chr6(+):5768274-5768493 Distal Distal 6 5728274 5728493 +

chr6(-):169288736-169288955 Distal Distal 6 169324461 169324680 -

chr7(-):46877757-46878022 Distal Distal 7 46972342 46972607 -

chr7(-):153942390-153942609 Distal Distal 7 155129163 155129382 -

chr7(-):65008173-65008398 Distal Distal 7 65780003 65780228 -

chr7(-):61707157-61707480 Distal Distal 7 62478983 62479306 -

chr7(+):149264065-149264284 Distal Distal 7 150409369 150409588 +

chr7(+):99993968-99994187 Distal Distal 7 101070791 101071010 +

chr7(-):127890130-127890441 Distal Distal 7 128967024 128967335 -

chr7(-):43658635-43658897 Distal Distal 7 43753213 43753475 -

chr7(-):57079832-57080143 Distal Distal 7 57452327 57452638 -

chr7(+):127885902-127886149 Distal Distal 7 128962796 128963043 +

chr7(+):44424974-44425315 Distal Distal 7 44519556 44519897 +

chr7(-):65006938-65007225 Distal Distal 7 65778768 65779055 -

chr7(-):71472583-71472802 Distal Distal 7 72244421 72244640 -

chr7(-):51098142-51098361 Distal Distal 7 51375999 51376218 -

chr7(-):1026075-1026324 Distal Distal 7 1126037 1126286 -

chr7(-):133366460-133366679 Distal Distal 7 134440152 134440371 -

chr7(-):72046554-72046773 Distal Distal 7 72818396 72818615 -

chr7(+):1041676-1041941 Distal Distal 7 1141638 1141903 +

chr7(+):45549856-45550075 Distal Distal 7 45644438 45644657 +

chr7(-):127885902-127886149 Distal Distal 7 128962796 128963043 -

chr7(-):127886710-127886929 Distal Distal 7 128963604 128963823 -

chr7(-):72489144-72489360 Distal Distal 7 73260996 73261212 -

chr7(+):30414648-30414867 Distal Distal 7 30514643 30514862 +

chr7(+):44978989-44979208 Distal Distal 7 45073571 45073790 +

chr7(-):72671831-72672050 Distal Distal 7 73443683 73443902 -

chr7(+):99903085-99903396 Distal Distal 7 100979908 100980219 +

chr7(+):156538951-156539170 Distal Distal 7 157654686 157654905 +

chr7(-):61435533-61435794 Distal Distal 7 62207358 62207619 -

chr7(-):38996537-38996756 Distal Distal 7 39091114 39091333 -

chr7(-):65008455-65008696 Distal Distal 7 65780285 65780526 -

chr7(-):44910161-44910426 Distal Distal 7 45004743 45005008 -

chr7(-):61844494-61844808 Distal Distal 7 62616320 62616634 -

chr7(-):54809646-54810030 Distal Distal 7 55087504 55087888 -

chr7(-):155506835-155507072 Distal Distal 7 156693608 156693845 -

chr7(-):127911094-127911322 Distal Distal 7 128987988 128988216 -

chr7(-):56653019-56653269 Distal Distal 7 57025514 57025764 -

chr7(-):56439439-56439660 Distal Distal 7 56614161 56614382 -

chr7(-):99993968-99994187 Distal Distal 7 101070791 101071010 -

chr7(+):96684426-96684645 Distal Distal 7 97761240 97761459 +

chr7(-):49945090-49945309 Distal Distal 7 50222944 50223163 -

chr7(-):156677435-156677654 Distal Distal 7 157793170 157793389 -

chr8(-):58416875-58417140 Distal Distal 8 58527292 58527557 -

chr8(-):143244474-143244693 Distal Distal 8 143077318 143077537 -

chr8(-):43673158-43673374 Distal Distal 8 37694051 37694267 -

chr8(-):141821046-141821357 Distal Distal 8 141604589 141604900 -

chr8(+):143164071-143164290 Distal Distal 8 142997098 142997316 +

chr8(-):143164071-143164290 Distal Distal 8 142997098 142997316 -

chr8(-):41081800-41082019 Distal Distal 8 41070162 41070381 -

chr8(-):143374130-143374349 Distal Distal 8 143135998 143136217 -

chr8(-):103869563-103869782 Distal Distal 8 103757919 103758138 -

chr8(+):143562258-143562487 Distal Distal 8 143378950 143379178 +

chr8(-):142790241-142790460 Distal Distal 8 142611300 142611519 -

chr8(+):145344172-145344391 Distal Distal 8 145200393 145200612 +

chr8(+):94395494-94395736 Distal Distal 8 94506180 94506422 +

chr8(-):97504158-97504377 Distal Distal 8 97386753 97386972 -

chr8(-):142313857-142314076 Distal Distal 8 142097400 142097619 -

chr8(+):21497538-21497757 Distal Distal 8 21194266 21194485 +

chr8(-):94890629-94890894 Distal Distal 8 95001315 95001580 -

chr8(-):70885526-70885770 Distal Distal 8 70994986 70995230 -

chr8(-):142487329-142487548 Distal Distal 8 142270872 142271091 -

chr8(-):103981384-103981603 Distal Distal 8 103869740 103869959 -

chr8(+):92017443-92017826 Distal Distal 8 92127842 92128225 +

chr8(-):103661985-103662204 Distal Distal 8 103550341 103550560 -

chr8(-):143695856-143696075 Distal Distal 8 143557913 143558132 -

chr8(-):143562258-143562487 Distal Distal 8 143378950 143379178 -

chr9(+):65996737-65996965 Distal Distal 9 69597305 69597533 +

chr9(+):33390702-33390921 Distal Distal 9 33211255 33211474 +

chr10(+):111456422-111456641 Distal Distal 10 111887385 111887604 +

chr10(+):79434250-79434490 Distal Distal 10 79569233 79569473 +

chr10(-):98818762-98819027 Distal Distal 10 99249703 99249968 -

chr10(+):132246920-132247145 Distal Distal 10 132679696 132679921 +

chr10(+):71999176-71999391 Distal Distal 10 72240388 72240603 +

chr10(-):78379490-78379706 Distal Distal 10 78514464 78514680 -

chr10(+):81681361-81681626 Distal Distal 10 82124149 82124414 +

chr10(-):47746462-47746743 Distal Distal 10 47717808 47718089 -

chr10(+):132667943-132668162 Distal Distal 10 132917131 132917350 +

chr10(+):48792218-48792464 Distal Distal 10 49188782 49189028 +

chr10(-):70575545-70575764 Distal Distal 10 70833419 70833638 -

chr10(-):121983219-121983434 Distal Distal 10 122404180 122404395 -

chr10(-):79990763-79990982 Distal Distal 10 80125746 80125965 -

chr10(-):128394603-128394822 Distal Distal 10 128827351 128827570 -

chr10(-):104848054-104848273 Distal Distal 10 105279004 105279223 -

chr10(+):132336035-132336254 Distal Distal 10 132768811 132769030 +

chr10(-):1318391-1318610 Distal Distal 10 1312399 1312618 -

chr10(-):127735265-127735530 Distal Distal 10 128106020 128106285 -

chr10(+):133976853-133977118 Distal Distal 10 134318426 134318691 +

chr11(+):77847459-77847678 Distal Distal 11 76034735 76034954 +

chr11(-):75930064-75930283 Distal Distal 11 74116998 74117217 -

chr11(+):71246262-71246481 Distal Distal 11 69333167 69333386 +

chr11(-):114131727-114131946 Distal Distal 11 112157269 112157488 -

chr11(+):95014672-95015047 Distal Distal 11 93195114 93195489 +

chr11(+):77425044-77425263 Distal Distal 11 75612319 75612538 +

chr11(-):115397919-115398138 Distal Distal 11 113423510 113423729 -

chr11(+):80744098-80744317 Distal Distal 11 78931386 78931605 +

chr11(-):135335466-135335731 Distal Distal 11 133358024 133358289 -

chr12(-):104173891-104174156 Distal Distal 12 102798069 102798334 -

chr12(+):107768836-107769055 Distal Distal 12 106390501 106390720 +

chr12(-):114834668-114834933 Distal Distal 12 113725797 113726062 -

chr12(+):130367471-130367690 Distal Distal 12 128957158 128957377 +

chr12(-):2284650-2284869 Distal Distal 12 2269593 2269812 -

chr12(+):108118311-108118576 Distal Distal 12 106739976 106740241 +

chr12(+):63751404-63751623 Distal Distal 12 61983004 61983223 +

chr12(-):124504814-124505034 Distal Distal 12 123115076 123115296 -

chr12(-):52457025-52457244 Distal Distal 12 50714030 50714249 -

chr12(-):76146929-76147174 Distal Distal 12 74606677 74606922 -

chr12(-):50379345-50379590 Distal Distal 12 48609495 48609740 -

chr12(-):107742853-107743075 Distal Distal 12 106364518 106364740 -

chr12(+):106136091-106136310 Distal Distal 12 104757756 104757975 +

chr13(-):98327745-98327964 Distal Distal 13 101638572 101638791 -

chr13(+):67172599-67172841 Distal Distal 13 70975684 70975926 +

chr13(+):19163242-19163461 Distal Distal 13 22981242 22981461 +

chr13(-):66598628-66598847 Distal Distal 13 70401713 70401932 -

chr13(+):111134991-111135302 Distal Distal 13 112864835 112865146 +

chr13(-):36110897-36111116 Distal Distal 13 39922413 39922632 -

chr13(-):92786246-92786468 Distal Distal 13 96097074 96097296 -

chr13(+):18558567-18558786 Distal Distal 13 22376567 22376786 +

chr14(+):90896693-90896912 Distal Distal 14 95070050 95070269 +

chr14(-):98674295-98674606 Distal Distal 14 102848997 102849308 -

chr14(-):99951462-99951681 Distal Distal 14 103932168 103932387 -

chr14(+):99671134-99671491 Distal Distal 14 103651840 103652197 +

chr14(-):13719769-13719988 Distal Distal 14 14581705 14581924 -

chr14(+):98727629-98727866 Distal Distal 14 +

chr14(+):99708144-99708363 Distal Distal 14 103688850 103689069 +

chr14(+):99614135-99614429 Distal Distal 14 103594841 103595135 +

chr14(-):99432304-99432646 Distal Distal 14 103413010 103413352 -

chr14(+):93842768-93843033 Distal Distal 14 98017273 98017538 +

chr14(+):98645894-98646113 Distal Distal 14 102820596 102820815 +

chr14(+):98475032-98475271 Distal Distal 14 102649733 102649973 +

chr14(-):99675341-99675560 Distal Distal 14 103656047 103656266 -

chr14(-):99607730-99607949 Distal Distal 14 103588436 103588655 -

chr14(+):96839509-96839728 Distal Distal 14 101014170 101014389 +

chr14(+):99939489-99939708 Distal Distal 14 103920195 103920414 +

chr14(+):97410416-97410635 Distal Distal 14 101585077 101585296 +

chr14(+):99989847-99990184 Distal Distal 14 103970553 103970890 +

chr14(+):94969804-94970174 Distal Distal 14 99144189 99144559 +

chr14(-):31949199-31949418 Distal Distal 14 36081760 36081979 -

chr14(-):99190775-99191010 Distal Distal 14 103171526 103171761 -

chr14(+):99423748-99423993 Distal Distal 14 103404454 103404699 +

chr14(+):99952618-99952883 Distal Distal 14 103933324 103933589 +

chr15(-):33535415-33535680 Distal Distal 15 38355732 38355997 -

chr15(-):63837252-63837535 Distal Distal 15 68456503 68456786 -

chr15(-):95913516-95913873 Distal Distal 15 99803054 99803411 -

chr15(-):46854444-46854767 Distal Distal 15 51523438 51523761 -

chr15(-):68562381-68562600 Distal Distal 15 73163527 73163746 -

chr15(-):19429934-19430153 Distal Distal 15 24228858 24229077 -

chr15(-):67226738-67226957 Distal Distal 15 71826453 71826672 -

chr15(-):46855566-46855785 Distal Distal 15 51524560 51524779 -

chr15(+):33535369-33535634 Distal Distal 15 38355686 38355951 +

chr15(-):35276753-35276972 Distal Distal 15 40096986 40097205 -

chr15(-):66832373-66832592 Distal Distal 15 71443551 71443770 -

chr16(-):68920241-68920552 Distal Distal 16 68318732 68319043 -

chr16(-):89209094-89209313 Distal Distal 16 87961839 87962058 -

chr16(-):29527333-29527570 Distal Distal 16 29241252 29241489 -

chr16(+):85514525-85514744 Distal Distal 16 84327520 84327739 +

chr16(-):3726562-3726781 Distal Distal 16 3693208 3693427 -

chr16(-):89369201-89369415 Distal Distal 16 88362828 88363042 -

chr17(-):82954131-82954459 Distal Distal 17 80939264 80939593 -

chr17(-):41586347-41586643 Distal Distal 17 39855127 39855423 -

chr17(+):45658489-45658754 Distal Distal 17 43971427 43971692 +

chr17(+):75079869-75080134 Distal Distal 17 72578695 72578960 +

chr17(-):80952355-80952586 Distal Distal 17 78364427 78364658 -

chr18(-):60741580-60741891 Distal Distal 18 58917465 58917776 -

chr18(-):43717400-43717636 Distal Distal 18 42182872 42183108 -

chr18(-):56055337-56055556 Distal Distal 18 54230528 54230747 -

chr18(-):45117249-45117514 Distal Distal 18 43573514 43573779 -

chr18(-):46214058-46214400 Distal Distal 18 44666026 44666368 -

chr18(-):44706312-44706531 Distal Distal 18 43160539 43160758 -

chr18(-):61084244-61084463 Distal Distal 18 59259906 59260125 -

chr18(+):76386474-76386693 Distal Distal 18 74459076 74459295 +

chr20(+):56322272-56322491 Distal Distal 20 57319322 57319541 +

chr21(-):43005923-43006158 Distal Distal 21 45269354 45269589 -

chr22(-):16539177-16539396 Distal Distal 22 17973504 17973723 -

chr22(-):46986963-46987182 Distal Distal 22 48617484 48617703 -

chr22(-):40416993-40417212 Distal Distal 22 42045501 42045720 -

chrX(-):97883022-97883287 Distal Distal X 99683742 99684007 -

chrX(+):8621551-8621881 Distal Distal X 9217128 9217458 +

chrX(-):127052964-127053183 Distal Distal X 128912804 128913023 -

chrX(+):129481471-129481690 Distal Distal X 131341292 131341511 +

chrX(+):110089-110400 Distal Distal X 273799 274110 +

chrX(+):7504256-7504475 Distal Distal X 8179861 8180080 +

chrX(+):2575220-2575439 Distal Distal X +

chrX(-):38485130-38485391 Distal Distal X 39261644 39261905 -

chrX(-):115083131-115083381 Distal Distal X 116851630 116851880 -

chrX(+):1009488-1009753 Distal Distal X 1213757 1214022 +

chrX(+):7035409-7035628 Distal Distal X 7710675 7710894 +

chrY(+):27517507-27517801 Distal Distal Y 27216001 27216295 +

chrY(+):9842107-9842326 Distal Distal Y 9540625 9540844 +
