# Supplementary material for: Comparative analysis of genome tiling array data reveals many novel primate-specific functional RNAs in human
Source: BMC Evol Biol. 2007 Feb 8;7(Suppl 1):S14. doi: 10.1186/1471-2148-7-S1-S14 (PMC1796608; doi:10.1186/1471-2148-7-S1-S14)
Supplement: Additional file 2 — DNA sequence of these candidate sequences [file 1471-2148-7-S1-S14-S2.doc]

>chr1(-):109913398-109913617

ATCATGTTCCCTGGCTTAGCAGCAGCGACTCCTCAGTGGAACCCACCACCGCCCATGGGCATAGAGGGCACCCCACCACAGAGCTGTCCTATGACGGTGGCAGAGGTTAGAGTGGGGAGCAGAAGCCGGGCTCCCAGCCAGCCTCCCTGACCTCCCCCTGTCTCCCTGCAATGAGGACAGAGGGGAAGACATGGCAGGCAAGGAGTGGGTGGGCAGAGCA

>chr1(-):223812426-223812670

TTGCTGCAGCCACGGAGCCCCCCACTGTCCCCTTGGCCTCAAGCCTGCCCCAGGGCCACAGGGGAGTCCCAGCCAGTCTGTTGGACAAGGGAGGGCGCAGGGAGGGACCCCAGCTGTCTGGTGCTGACTGGCTTACAGATCATGCTGTGTTTATGCCAGCTCTGGACAGGCTGGGGCCAGGGTAGAGCCTGAGAACAAGGGACTCCCTTGCCCCCCAGCACAGTACTAGGCTTACTTCCCAAGTG

>chr1(-):224900444-224900663

GGCAGAGGAGCACCCTGGGAGGTAACTCCCTGAAGGAAGAGAGCTGTGGTGGCCAGTGACTGGGGCCTGGAGGGAGGAGTGTCATCTTTCACTTGGAAGAGCTGGGTAGGAATGGTCCAAGAGCTAGTGCATCTCCAACTGAAAGCAGGAAAGAGTGCAGGAGGCAGTCCCGGCTCCAGAGCCCCTGTTCAAGAGGAAGGACCAGGGCACCTAACGGAGC

>chr1(-):240661950-240662261

ACCTGCAGACTGGAGCACCCCAAGTCAGGAGGCCAAATTGCGGGCCCATCTCCAACCCAGTCCTGCCCACATGTAACCCAGGAAACACTGCTGTCTCACCAGCACGCCTCACCACACTGGGAAAGTTCTCCCGCCCAGTCCTGTAGCCATCCCTCCCTGCACGCAGAAACGGGAGGTGCACCCAGGAAGCCCTGAGGCCTGGGAGACCCATCTCCATGGAGCACCCCACGAGGCAAGCACAGCACTCAGGCTCACCTGGGGCTGTGCCCCCTGACAGGTGTGTGACGTGTGTGAGTGTGAGTGCGAGGTACA

>chr1(+):219559879-219560190

CCTTCTACATGATCCTCACCCAGCCTGGTGCTCCTCATTGCCCACTGTCCCCCATACCAGGCTCCTGACACCCTAAAGCCAAGGCCAGAAAGAGCCCCCAGCCTCCACTCCTGGTGCTGCTCTGAGCACCTGTCCTTCTGGTTGTTGGCAGGCTGTGGCGGGGGTGCCACCTCTCCAGGTTGCCTTTCTCAGATTCCTTCTGGTCTGCTGGCCCCTTCCAGGCTGACTTCTGACAGCACCCTTTACAAACCAGCCCAGCCCACATCTGAGCTCTGTGCCTGGCCTGCCTGGCCCACTGCTGACCCTGGTCAC

>chr1(-):223825600-223825845

CGAGCCTGGCGTGAGCACCTGCACCTGCCACTTTGCAAGGGAAGAAGAGGGGACAGAGGGCAGGGGTGCCTAGGACGTGACTTTGGGGAGGGCAGGGGTGAACCAGTGGCATCTAGGGGAGGGCCTGTCACCCAGGGGCTGGCGGGGGGTAGCTCTCCCGAGGGCAGCAGAAGGTCCTCAGCCTGGCCCAGTGCCCTGCACTTCTGCCCTGTGCCCCCTGTCTGGCCCCCTGTGTCAGCGGCCAGG

>chr1(-):242487143-242487434

TGGTAGGGAGGAGGAGGCTTGCCTAGCAGGACCATCCACCCCTGTCCTGGCCCCAAGTCCAGCCTGCCTGCCTGTCCTGGCCATTGCCTTGTCTGGGAGGCCTGGGCATGATGGTCAGGTGGTGTGTCCCTGGGTTCCAGGCCTTGGTGGTCCCTTGGGAGCTGAGCATCCTCTGGGCAGGGGGAGGGCCCAGGGTCCACTGTTTGCATCTGCCCCTGGGGCTGTGCCTCCACTCCAGCTTCTCAGCCCTCATGGGGATGTGGCCAGGGCTCCTCACCTGCTCTGTGGCTCC

>chr1(-):29870130-29870395

GCAGTGAGGGAGTCTGGGACCTAGAGAGACTGGCCAAGGAGGAGGGGCCTCCCCTTCCCCACCTAGGCCCCTTCACCAGGGCACACTGCAAGGCACCCATGTCTGTGCCAAGGTCTGTGCTGGACACTAGAGGTCTGAGTGAAGTGACTAGGACCTTGCCCCACTCACCCAGCCCCTGCCAGCTTCTGCCTCCACTCCTGCACGTGGCATGGGCTCCTGCAGTTCCGCTGAGCTCTGGAGGTGCCCTGGTCTGGGAGACAGAGATG

>chr1(+):223825148-223825367

ATGAATCCTGGGAAGTTGATGGGACTGGTTTCTACCATGGGGGACACCACAAAGCTCCAAGCTTGGCTCGGGAGGCTGGGCACAGAAGCCCATCCTTGTCCCAGCTCTGCAGACCCCACTGTCCTTGCCAGCCCCACCCTCTGCACCTCCCAGTCCCTTCAGATATTTCAGTCCCCAGGTAAGTGCTTCTGTCTCCTGACACAGGTGCTCCTGCACACAC

>chr1(-):89797378-89797597

GCAAGGTGGATGCCCTACTCTCTGCCAGTCAAAGCCCTAAGGGCCAGGGCCAGGGCACTGGGACCTCTGCTTCAGCAGGAAAGCTAAATGCTCCCCATGCCGTGGGCCAGCAGAGACTTGACTCTGGCTCACAGGGCTCCACTCCTGCCTTCCCAGCCTCAGTCAGGAACGTCTGATTGTGCAGCCTCAATCTCATCCAGAACACTAGCTCTCCAGCCTC

>chr1(-):196681122-196681350

AAAGCCCTCCTCTCACTCCCCATGCACCATGGACTCCAGTCCCCGCGGGCTTCTTTCTGGCTGTCAGAGGTGCTCTCCCGGGCCTCCCACTGCATGGTCCTCTCCGCGCTGGAATGCTGTCCCCTCACCGCTTAGCCTGGAAGCCTCTGCCCCGCCAGGCCTCAGTCCAGGCCTGCTCAGGTCCCCGTGTTATATGGCCCCACAGCACCCAGCACTTTCTATGGACTAG

>chr1(-):240669261-240669531

CATCTCGAATGGCTGGAGATGAGGCTGGGTGAGTCAGGAATGAGCCTTGAAGGCCACCTGAGAAATGGGGACTGAGCTCATACCACAGGCTAAGGGGATCCACCAGGGCCGAGTCCCCAAAGCAAGCACCTTCCAGACCTTCCAGAGGCTGCACCCAGTGCTCCCGCTGCCGCTGTGAAGCACTGCCCACAGGAGGGAGCGGGCAGCCAGCTGGGGCTGCCATGGGCCCTGGACGGGGCTGTGTCCGCCGCCATAGCAGCTGTTCCCGTGG

>chr1(+):19152997-19153216

TAGCAAGGGTTGGTGGGACAGACAGTGCAATTCTCAGCTTCTCAGCTCTCCATGATGCTGGAATGGGGGAGCCCATGGTTCCAACTCTATGGATTGTCTAACTGGACAGGCTGGGACCCCAAACTCTTCCAGCTGCACAAGCATGGGGGCAGCTGTGGTGTGGGAATGCGTCACCTGCTGTGTATACTGGTTCTTGGGGGCCTCCACCCCTTGATCTGAT

>chr1(-):222045540-222045805

AACAGGACAGGGTGTGTGGGCACAGGCAATAGCAAGGACCCACAAGGGGGGTAAACGGTGACCCTGGGGCCTGGCACAAGTGGGCTCTGTGGAGAGTGGGGGCCTGGGAGTGAGGTGGAGTAGAGTTGTATTTGACAGCAACAAGCCTTGCCGCTCTCTCTCCTGCCCATCCGTGGTGCAGAGGCTCCTGGAAGCTGGAGGGCAGTGGAGGCTGCCTGATGGAGTCTCCTGGAACACGGAGCAGTTGTGGAGAAGTGAGGGTCTTG

>chr1(-):46826901-46827120

GGCTGAGGGGCTGGGGAAGTGGCTCCGTGTATATGAGCATAACCCTCTCTGCCACCCAGCAATCTTGCATCCCCAGCTTCTCAGTGGGCTTCGAGGAGAGAGAAGCACTCCAGCCCTCTCCACATCCTTGTTTCCCCCATGGTTCCTGGGTGTCTAGGCCCTGCTTCTCTTCTAGAGATAACCTGGGGGAGATGCTGCCAACCCTCCTGGTGACCCAGCA

>chr2(-):173349489-173349754

CTGCCAGGCCCTTCTGTGGAGGCTGAGGAGCAGGCTTGGGTGGAGAGACAGAAAGCTTAGGTGGGGCTGAGCTTGCTGGATGCATGAGGCTGGCAGGAGGGCCTTGTGAGGGAAGGCAGGAGCAGCCCTTGTGCACTCAGGTGCTGAGGAAGGCTGCTGGCAGGTGTCTGTGCCTTTGCTGCCTCTTGCCTCCCTGGCCCTGATTCTAAGAATGAGAAGTGGGATCACTGAGCAGGAATGTGAGGAGTGTTTTGATTGTCTCACGG

>chr2(-):908059-908370

TTTGCTGCTGATGAGATTTCCTCTCACAGAGCTCAGGGTTGGAAGGCCCTTCTCCCGGCCGAAGTTCCTCCCTGCTTTGAGCCCAGCAATCTCTGTGGGGCAGGGGAGGCTTCCCCAAAGGGACAGTGAGGGAGATGGCCTGTCCAGAGGCTCCCAGTCCAGGCCCACCAGCTCCCAAGGGCAGCCCGTTGGGGCAGGGACGGCTCTAGCAGTGGAGTCAGCGGCCATGTAGGCAGGTGGCTGAGCACCTGAGCACTATGCCCTGCGGTGTATCTCAGGGTGTCTGCGGGGACCCTGGGCACTCACTCCTCT

>chr2(-):217195840-217196059

CAGGAAGGCAGTGCCCACCTTCCCCTGCACCTGCTCCATCACCTCTCCCACACTCACTGTCCAGAAACTCCCTGTTGCTGCCTGAATCCCACTGGCTGAAGGCGAGTATTTCTTTGTCCTCTAGCCCCCCTTCCTCACCACTTAGATAAGCAGCAGCATGTGGCTCCAGAGCAGAGTGGGCGAGACCCTGGACCTGGCCGTAGTTCTCATGCTGCCTGTC

>chr2(-):43466979-43467198

CCACCCCTTTGGCCTCTGCTCCCTGACTCCTGCTCTTTCAGGGGCCATGGGAGAGAGAGGCCTGACCCCTGGATTTTCACTGCCACCTCTTAGCCATTAGCCTGGCGCCTGAGGCCCTCGTCACACCCCAGGCTATGCTTGGGGCTGGGAGCCCTTTGCTGCCCACGCCTGCCCCTCTCAGGAGCTGGAAGAAGCCGCTGCTATGATAGAAGTTTCCATC

>chr2(+):43257560-43257782

AGATTTCTCTTGATTGGCCCCAGCCCTGGTTGGTGCCTGTCTGCAGACAGGGCAGGTAGGGCTGTGGGTTCGAGAACTCCTCTATGTCCCTGGGTCCAGGGCCCTGGAGAAACCTGTACCCACTCCTCCCCACAGGAGCTAGTGGTCGCTGGGACCTGGTTCCAGATTTGGCTGTAGCCTCTGAAATGCGGGCAGAGGAGGAGGGTCCCAAGGGACTTGTGCT

>chr2(-):127290009-127290277

CGTGCCCCCTGCTGGGCTGGGAAAGGCCTCCCCAGCACGCATTCCCATGCACCCGAGTGCACCCGGGACTCCCGAGACCCAGAGCACTGCTTGTTTTTAGGCAGGGCTCTGAGTGGAAAGGGCTGAGCTGTGCCAAGCTCCTGGACTTGGTGCCCTCGGGGCCAGGGATGACTGCTCTGCACCACCGGGGCGTGCCAGAGTGACCTGAGACTCCATGCCTGGCTGAGAAAAGTCCTCTCCCCAGTGCCGCTGTAAGTGCCCGGGCTTTG

>chr2(-):216580178-216580397

CAACCTGGGGCTGAGATCCACCTGTCAGAATAGACATGGACACCAGAGGAGCAGTGGCTTTCACGTTCACTGGTGACTCCATGAGCCCACCAGACAACAGGCCCTGAAGGCTGAGGGTGGTTCAGAATCCAGAAGAAAAGCAGCTCCACTGTACTGCCCTGGACCCTGGTCAGCCTTAACACAGGCTGTGATCATCTCAAACCTTTCGAGACCTTTATCC

>chr2(-):15962542-15962784

CCCATGGCATGGGGGTACCGGAAGGTAGTCCCCTCACTCTGCCTGCCCTTCACCTCCTCTCCCTGAGGAAGGCATGGGGCATGTCCTGGGCATTATCAGCTCCACTTTCCGAGGCTCAGAGAGGTCCCTCATTTCAACAGCAGCCCCCGTCCCCACCCTGGGTCTCCTCAGCACCCTGTCAGCTCCATGTATCCCAGACCTGGGCCCAGGGGAGCTTTCCTCAGCAGTGGAGCTAGGCCCTCT

>chr2(-):118994004-118994223

CCATCATGTTACAAGGGCCACAGTGCTGCTGCGGGCATTTCAGCACTGTGCCGGGGTGGGAACCAGTGTCTGGCTTTGCCTGGAGAAGCAGAAGGGCACAGTTGGGGGAAGTGTGCTGTGTGCTGTGGCGGCAGGGTGGGGAAGGGTGCAGCCAGGGCCAAAGGCACAGTGCTGAACACGCAGAAAAGGGCAACAGGCACACGATGGGACCACAGCGTGT

>chr2(+):129987126-129987462

AGGAGGAGCACCCCACATTGCACTCCTCTCCGGGCTTTTCACCCTCCTCCCCAAGCCCAGCCCTCGCCTGCAGTGACTCACACTGAACCCCAGAGCCCCTCAGACCATCAGCACCAGCTGGGCCTGTGGGACCCAAGGCACCCACAGCACCTGGAGGCCCTGAAATGCACACAAGGAACGTGTCCTGAAAGGCAGCAGAGGGGATGTGAATTGGAGCAGGGGTTCAAAGTGGAAGGGGTGAGGCAGGGAGGAGGGCCCGAAGCCTGGGAGCCCCAGAGCAGCAATAACTATAGGAAACCCCATCCCCAGGAGGGGTCAACATGGGATACATGGATGG

>chr2(+):18604689-18604908

AAGGACCAAAATAATAATCTATCAGCATAGAAAGCTAAGGATTCTTCATAGAAATCTGTGTGCTCATTCCTGGGCTAGGCTATGCTGTAAGCCACACTCTGGACAAGACAACCAAGAGCCCAGTGTTCATAATATATGGGCTGATGGGCGCAGGGACTATTTCAAGTGCATCCCAAGATGCAACAATAGATGGCTACTTTGGCCAAATAAACCCTGACCA

>chr2(+):119459480-119459745

GGAGCCATGCCCCTGGCCTGCAGTTGCTGCCTGGCCTGGCCTGGTCCTGCTGCACCCTCACTGCCCAGCACTGAGGGCCAGTCCTTTGAGTGCGGGGGGATGAGGAGAAATGGCCTCTGATAGGGTGGTGGTCATAACTCCCAGGAAGAATGAGGCTGGGGCTTAGGGGCCAAACCCAGGGGCCAAAACCAGGTGGGCAGAGGCAGATTCTCCTCCCTTGCCCTGCCGGGGTCAGAGCAGCAGCCCCCTCTTCCCATTCTGAGGCC

>chr2(-):10445305-10445524

CTGGGGCCCAAGCACCCACAGCCTCTTCCCTAGCCCTCTGTAGACAAGGAAGACCCGGCTTGAGGGCCTCTTGCCCCAGAATCATCTTGAGAGCCCTGAGTCCTGCCCGACCTTCCGCCCTGACTGGAATTCTTGGGCTGGGCCTGGGCTGCCTGTTCATAGTGACTGCCATGAACATTCGAATTTGAGAGCCTCCAAGGAAGGGGACAGCCCCAGGGAA

>chr2(-):6176618-6176837

GGGAGAGACTTTGCAGGATAATTCTGGGGCAAATCCAGGCATACGCCAAAGAATGCTGATTCCCAGTGCCTGAAAGGGGGGAAGGTTTTCGCAGCTTCCAGGAGAGGCGCCTGGTGGTAAGGAAGGGTGGGCCCACCACACTGGGACTGGGAGCCACCCCGAGGGAGAAAGTGCTGCAGTCAGACCTGCTGCTAGAGCCTGGGTCTGGGCCACATTGCCT

>chr2(-):217608918-217609206

CTAATTTACTGCCCCTGTCCTGGGACCTCCTCCCCAGATTGGCCTTTGAGCCTGAAGGCACCTGGCCCTTCTTCCAGCCAGACGTGAGGCCACGTGAGGGGAGTGTCTGTGGCAGTCACCTTCTTTGAGGTTGGGGTGGGGTTGGGCCTGTTGCCCCCTGGCTCAGGGTGGGCGAGGAAGCCCAGCTCCCTCTCCCTCAGTAGCATCCTTCAGGTTGGCTCTTCTGACTGCCCCCTCACCAGGAGGTGACTTCTGGCCCAGCTTTCTCCCCACCCCTGACAGATGGAGT

>chr2(-):217580701-217580932

GGGGCATCCCCTGTGGCTGCTTAGAGAGGCCTAAGCAAGGGCAGAGATGGACCTGAAGCCCTGTCCCTGCCCTCCAGGCCCCAGGGGGGAAATATTTTGTAAACTCAGATTTCTTGGGGCTGGGCTGCCAGGGCAGGCAAAACTGGGGCCCCTGGGAGAAGCTGTTCTGGAAGGCTGCACATGCCACCCATGGGGTCACTGGGCTGCCAGGGCTCTCTTCTGGGAGACTCTG

>chr2(-):108392031-108392296

GTGGGATGGGGCTGGCCTGGGTAGAGGGTACTGACCCAGGTTGGGGGCACTGACTGGGTTGGGGACACTGGCCTAGGTGGGAGGCATTGACTTGGGTGGGGGGCACTGGCCTGGGTGGGAGGCACTGACTTGGGTCGGAGGCACTGGTCTGGTGGGGAGCACTGACCCACACCAGGCACCCCTCTGGCTCTGGAGGAGGTATGCCCAGGGTGGGTACACAGGCCTCTTGCAGGCGGCTATGTGGGCTGCTCCCCTGGGGGGCAGAA

>chr2(+):127202283-127202502

CAGGCCTAAGTCACTGCCCACTTCATCTTGGGGCACACAGGGCAGGGCAGAGCTGCCTTTGCTGAGTCACCAGGCACACTTCAACACTCCGGCCCCAGCCAGGCACCCAACAGGATTACATCAGCAGCCTCAGTTTCTTCCCCATCCCTGGCCTTTTCCTGTGAGTCCTCCTTATGGAGACCACACTCCTCCCGGGAGGGGCCTGGCCTTTAAGCCCAGG

>chr2(-):820100-820350

GCAAGGGTGGGGGGCTCCAGGATGGGAGAAGCACCGCCGAGGCAGCTCCTGAATCAGTTGCCATAGGGCTGGGGCTCCCACCCTGTTGGTGTTCCGCCCTGTCCCACATCAGCTCTGGTCTTCGCTGACCACCCTTCTCCCTTCTCCCTGTTCTACCCACCCCTCTCTCTTGAAATCTCCCCTCCCTTGGCATCCTCTTTCCTTCCTAGAGTTTCCTGATGGTGGCATCCTGGAGGAGGCTCTGCGTCCCC

>chr2(-):125344276-125344495

GCCCAAGGCTGTGGTGGGTCGTTTTCTAGGCTTGGGAAACTGGGTCCTAGCAGACCAGCAGGTGAGTGTGTGCCAGGCCCGCGGCGTGTTGAGGGGCCTGTCGTGAACAGTTGTGAGAGGTGCAGCTGGAAAGGCAGCAAGCCTGGGACGGTGCGTGAGGGAGGGCTCGAGGGCCACCCCGAGCCAGCTGCTAAGGCGGGCAAGGGCAGCAGCAGCCCAG

>chr2(+):10445351-10445570

TCATGGCAGTCACTATGAACAGGCAGCCCAGGCCCAGCCCAAGAATTCCAGTCAGGGCGGAAGGTCGGGCAGGACTCAGGGCTCTCAAGATGATTCTGGGGCAAGAGGCCCTCAAGCCGGGTCTTCCTTGTCTACAGAGGGCTAGGGAAGAGGCTGTGGGTGCTTGGGCCCCAGGATTAAAGAAGTGAGCCCTGAGGCGAGTGTGGTGGCTGCTCCCTGG

>chr2(-):2234328-2234547

TTCTGAAAGGGATGGAGGTCAGGCCAGGAATGAGACCTCAGGGAGTGGGCAGACCACGGGCACCCACTGTGCCAGCTGGAGCCAGGAGGGGCTGAGGGCACAGGCTGCCTGTCCTGGTTGCTGCGAGGGCTGCTCCCCTCCCTGCTTGTCTGCTCCTGCAGCCTCGGGCCATCGGGAGCTGTCAGCATTCCGAGGGGAGAGACACCTGATTGTCTTTGAT

>chr2(+):127290962-127291181

ACCACAGAGCTTCCGTGGAGGCAGGAGGTGGCAGAGCGCTGCGAGGCGGCTGTTCCAGGGCATGGGGGTAGTAGTGGCAATGCAAGTCCTGCCTGCCCCAGTTGCCAAGAGTGAGTGGGGAGACAGGAGGGTATGCCTGCTGCTGGGAGACCATGCCAAGCCGCCCTGTTTTCAGTTCTCATGTCTAGGTTGACAGAGCCACCTGGGAGCTTGAAGCCTC

>chr2(+):130877035-130877254

GCTGGGAGCGGCCCTTCAAGGGATACAGCTGGTGTTGCCTGGGGACGGGGAATCCTCATCCTTCCGGCCTCCCTAAGGCAGCCTCCCCTGCCCATGACAGTGACACACTCAGCAACTCGGCCCCTAGCCCCTTACCTGGGTGCTGCTCCAGCTGTGCCCCGGTGGGCTGTGCTACCCCCAGGTCGACTCCTCCTTGTCCCTGGCACTGCTGCGTCTCCCC

>chr2(+):217620591-217620856

GAAAACAGCCGTCCTCCCAGGCCCCTGCTCCACTTCCCTAATACTAGCCCCTGGAGCAGCCGAGCCTGGAGAAACGAGGACCCGGCATGGAAAGACCCCAGGCTCAAGGAGGCAGAGCACAGCCAGGGTGGGGGACCCCAGCCCACCGGGGAAACCCGCCCGGCCCATTGCTGCGCACAGGATCTCACTGGTGGTCTCACATCCATCCTTTTGGGAATGGGATCAGGCCTGGGGGGTCGGCACTGATGACCAGCACTCAGGGGGAC

>chr2(+):1876547-1876766

GAGAGGGGGAGGCCCAGGTGCAGGGGCCGGAGAGCTTATGCTTAGGGTGCAGTGGGAGGGCAAGGGTTGCCTCGCACATGGGAGACGCCAGGGCCGGGCTGCGGGTGACACTGTGGCCAGGCCTTCCTCCCTGGGCCTCACCCTGAGCTGGGCAGACACTGACCGGGGCTGGCTCTGGCTCTGGGCGGAGGTGCTGGGGGGCTCACTGCCCAGGTCATCT

>chr2(+):15988465-15988684

AGGTTTTCTGAGGAGGGTGGGTTTGAGCTGGGGGATTCGTCCACTCCCTGACCCCACCTTGGCCCTCTCCTCACCTTGGCCTTTCCTGGGCAAGGGGCCATGGGGCAAGAGACCTCCCCAGACCTCCAGGTCCGCACCAGGCCCTGCAGCTGGCAAACCTCCTCATCCCCAACAAGGGCGGGCATCTCCCAGGCAGCACCCCCTCTCCGGAAAGCATAAT

>chr2(-):127130799-127131018

CTCTTGAGGACAGAGACAACATGCCAGCACACAGCTCCTAAGGAGCCTGGGTCTCATTGACCTACACAGCAGCCAGGCCCATGAATACCCACCCACGTGGCCAGGGAAGGTTGGGGGTGCCTCCCTAAGGGGCCAACTGGGCTACTGCCAAGCAGCTGTCTGAGGTTGCCAAGGGGCACCCTGACCCGGGAAGTCACTGTCTGGGTCTGGTTCTCACATC

>chr2(-):241019276-241019495

GGTGCTGGAGAGGTCCCTGTGGAGGACAGGAGGTGCTGGAGAGGCCCCTGTGAAGGACAGGAGGTGCTGGAGAGGCCAGTCCCTGTGGAGGACAGGAGGTGCTGGAGAGGCCAATCCCTGCAGAGAAGCGGGGGTTTCTGAGAGGTGGGTGCATTGGTGTCTGGATGACGGAAGACAGCTCCAGAGGGCCTCACACCAGGCAGGAGGGAGATTAGGAACC

>chr2(-):108426989-108427208

TGCGGGGAGCTCGGGACAGGAGAAAGCTCTGTTCCTCACACATCCTGGGACTCCTTAGGGGCAGAGGCAGCAAAGGAGCCTTGTGCTGCTTCTGCTGCACTCAGCCCCAAGCCCGTCCAGGAAGACCTGCACCCTGCTCGGGGTGCTGGGGGAGTCGCGGGAGTGTCAGAGCTGAGCTACAGGCTGAGAAGGAAGGAGCAAGTGCAGCACCCCTGGGCGC

>chr2(-):129483425-129483690

GGGATCCTGGGCATCCCTGGGAGAATTGGGGCTCCCCCAAGAGCTCCTGGGACCACTGCTGGGAGGGGCCTTCAAGGTTAGGGTGGCCGGGTAGGGGCTGGGCCTGACCTGAGACCCTGCCCACCAGGCCTCTTCCAGGCCAGGTTTGCTGTGCTGGGTGGGAACGTGGGAGAAGTCACTCTCTATCTGCGGCCCCCATCAGTGCCTCTGCCACCCGGCCCAGAGGGGGCAGGGACTCTGGGGCGGACAAGGCTGTGGGGGTGGTG

>chr2(+):20807867-20808132

GAAAGCAGTAGCCACCCCACCAGCTTTCTTCTTAGAAATCTCCCAGCAAGCTGTCGGCTGCCTCTCCCTTAGAAACGGGGCTCTAGCAAGACACGAAGTGTGTCCACTGGAGGCAACCCTGAGGTCCCTGGGCCACGAGCAGTTTGCGGGCGGCCTGAGGAGGCAGGCAGAGCTTCCCCCAGGCTGGAGGGGCCGTCTGCGGGCACTGGGGCTGGCAGGGAGCATTTCAGTGGCCCACAGCCCTCGTCCCTGCCACGGGCTTCTGC

>chr2(-):118403040-118403276

AGGCTGCTTGGAGTGGCCACTGTGGGCCCCACCAGGGCCTTGCCGCCTCCTCCCAGACAGGGGTGGAACCACAGCTGCTGCCACTTCTACTGCCTAAGGCGCAGGGATGGGGGGTAAGAACCCAGGGTCCCCTCACTGCCCCCTGCCCATCAGGCTATGGCAAGAGCCTTTCCTCACCCCAGATGCTGCCTCCTCAAATGGAGCATCCCAGCTCTCCAAACCACCAGAGCCCGTCCT

>chr2(-):203405997-203406216

CATATGATTCTCTGCACAATCACCATCCTACTCCACCCTTGGGGGTAGGAGGAAAGACAGAGCTTGCATCCCAGAGTGCTGCCCCTCCTGTCCCCACAGGACCAGGGAGGAATCAGAAAACAGGGGCCAAGTCCATTGCTGTACTGGGAAAATGACACCCACAGTATTCTATCCCTCAACTCTACCATGAGTTGGTGAGTTGGCACATTTGGTCACCTTT

>chr2(-):72528946-72529165

ATCAGGGGCAGGAACTAGACGGCTGCTCCAGGGGCAGGTCTAGTTCCAGACCCCAGCTCCCCCACAGCCTACAAGCCTCAAACTTCTGTTCTCATGGGCTGGGTGCCAGAAGTGCTGGTCTGTGGGACTCCACACGCCTGCCATTCCCACCACCGCTTCTCAGGAAAGCAGCCTTGCAAGCAAGTGCTGGGAGTTGTGGTCTGGGACCCTGTCCCTCTGG

>chr2(-):119441829-119442048

GGGCACGATGGCTACAGGGCGTGGCTTCTTGTCATTCTGCCCAGAGGGCTGTGAGTAGAAGGCAGGCCCCTCTGTCTGCAGGGGAGTGGGTGAGTGGACCCACAGCACACGGGGTTTCTGAGCTGGCCCCTACAGCATTCCCCAAGGGCCAGACAGCAGTGAGCCCCCCAGAGCACCCACTGGATGCCTGGTCTTGGCTGCCACCAAGCCTGCTGCTCAG

>chr2(+):10445305-10445524

TTCCCTGGGGCTGTCCCCTTCCTTGGAGGCTCTCAAATTCGAATGTTCATGGCAGTCACTATGAACAGGCAGCCCAGGCCCAGCCCAAGAATTCCAGTCAGGGCGGAAGGTCGGGCAGGACTCAGGGCTCTCAAGATGATTCTGGGGCAAGAGGCCCTCAAGCCGGGTCTTCCTTGTCTACAGAGGGCTAGGGAAGAGGCTGTGGGTGCTTGGGCCCCAG

>chr2(-):217599433-217599744

GGAGAGGGCCATGTCACATGAGCAGGGGGACAAGCAGTCCCCAGAGGATAGGTTAACCTGGGAGGATGGTGACCAGCTCTTCAAAAGTAGCACTGGGGAGGGAGATGCGAGGAGCTGGGGGCCACTGTTGGACCTCCACATGGGGGCTGCAGAGAGATCTCTTCATGTGATCTGGCATGAGGCCCCCAGGGCAGCCCAGAACCTGCCCAGGCTTCCCTGATCCCTCCCTAGGGGGGATGTTTTGGCCTCTAATACATGGTTCCCTGTGTCTGAGTGTCTGTGTCTGGTCCACTGTGGGGAGCTTTGCATCTC

>chr2(-):1916578-1916797

TGGATGGGGGTGCCAGGCTCACACCCTCACTCAGTGAACCCCAGGCCGTGGAAGGGCAGTTGCTTTCTTGGCCTTGGGGAGGTGGGTGGCCATTGAGCAATGAGGCTGCCCAGAAGGGCACAGGCCCTGGAGGGGACCCGTGCTGGCACAGCAGGCTGGCCTGCCTAGGGGGGCTCTGGACACCAGCTGCCCTCCCTTCTGCCTGCTGCGCCCCCAGGCA

>chr2(-):70427387-70427606

CGTGCTCATCAGGCTGTTTACTCTGGGGGCGGCTGTGCACTTAGTGCCCCCTCCACGCCCCAAATGAGCGCCGGGACTGGCCAGGACTGGCAGGGCTGGCTGGGCAGGTCCAAGGGCTCAGGGACCCGCCCCAGGGCCTCCTGGGCACACCTGGGGCCCACAGCCCCCCATTCCTGGGCCTTAAGGCCTGAACTCTGAGGCCCCCCATGTACGTGCACCC

>chr2(-):127028929-127029148

GGAGAGGCCCAGGGTGAGAGGTGACAGCCCAGCTGAGGCACCGTGGTGGCCGCCTGTGTGTGCCCAGTTCACAGGAGCCTCACCCTCCAGGAGGCCTCACTGAGCCGCAGCAACCACGAGGCTTGCCCTCCTCACTCACCACGTCTGCCCTCACCCACCAGGCCCGCTCCTGCCTGGAGGACGAACCACATGGCTGTCCTCCCCACCCCACCTAGTTTCC

>chr2(-):109071690-109071909

TCCCTGGGGCCCTGGGAGAATCCTGGGTCAGATTACAGCAGCACAGGTCACCTGCTCCAGCCCAGACAGGCCCAGGATCTGGCCCATCCACTGGCCCGGCCCTTCCTGGCCACCACTGTAGCATGTCACAGCCTGGGCTCTGGGTGGGCCTGGTCCCTCTCCTGGTTGCCGCCACTGCAGGTGAGTCAACAGCGAGACAACACAGTAACACCCTGCACAG

>chr2(-):97841928-97842219

TTGGGCGGGGAGACGGCTGAAGTTGCCAAGAGAGAGCTCAAAGGGAGGCAGGGCTGAGGCCAGAAACCCTAAGGGATGGATCAAGGAGAGGAATTGAGGAAGGACAGAGAGCAGGAGCATATGGACAGAGGAGAGGCAGAAGCCGGGCTCTGCAGGGCTGTGGAAGCTGGAGGAGTAGGTTGTCCCAGAGTCCCGTACCCCTGGGGATCCAGGAAGGTAAGAGGGAAGTCAGCAACAAGGAGGTGGGAGAGGTTCCTCGGCATGTGGGGCGGAAGCCAGACTGTGGTGATTG

>chr2(+):6673364-6673583

TCTGACATCCATGTCCATGGGAAGCTCCTGCCCTGTCCTCAGTGCTTTCCTTGCTGGACAGGCACAGGCCCTGGGGAGCTGGCGTGCAGCCTCCCTCCCACTGCCCACGGCTCCCCTCCCACTGCCCACGGCTCCCCTCCCACTGCCCACAGCTCCTCCACATCAGTGCCTGGTTCCCTGCACCTCCAAGTCATCGTCAGCACCCACAGCACCAATTTCC

>chr2(-):95192208-95192427

GATGGTGAGGGTGCAGATGGTTCATGATGACAGTTCCTGGGAGCTACAGGAGAAGTGAGGAGGGCTGGTGGGCCTGTGGCAACTTCCCACAGCTCACAGGAGGGCTGGAGCTGACCCGGCTGCTCCGCTCTGCTGCAGAAACAACAGCTGCTGCTCCCTGGGCTCAGGGCTGTGTCTGCAGTAGCCTGGGGACACCAGAGCCCCAACCCAGTGGAGCCCC

>chr2(+):95192116-95192473

TATTTTGGAAATGAAACAGTTTTATATCCCTAAAGCAAGAGAGATGCACACCAGCTTGGGGCTCGCACTGGAGAACACAGAACAGCAGGGCAGGGGCTCCACTGGGTTGGGGCTCTGGTGTCCCCAGGCTACTGCAGACACAGCCCTGAGCCCAGGGAGCAGCAGCTGTTGTTTCTGCAGCAGAGCGGAGCAGCCGGGTCAGCTCCAGCCCTCCTGTGAGCTGTGGGAAGTTGCCACAGGCCCACCAGCCCTCCTCACTTCTCCTGTAGCTCCCAGGAACTGTCATCATGAACCATCTGCACCCTCACCATCCCTTAGGGGCTGGGCAGTGCTGGGGCTATTATTCTCCTTGACCTTG

>chr2(-):23826104-23826323

GCTGTCCCCCAGAAGCCTGCCAGTCCTGACTCGGCCGCCTGTCACTAGGGCAGGAGATGGCTTGAGAAGGCATCTCTCTCAGCCCTACACCTGCCCAGCCTTGGGATCCAGCCACCACTTGTCTCCCTTAGGGCAGGAGCCGCCCTCCCTCTGGAGCTCTAACCTCTTGTCATTGTCACCAGCCACTCACTAAGCTCTGCTCTCTGGGCCAGGTGCCACC

>chr2(-):173538443-173538708

CAGCACAGGCCCAGAGTGCGTCCCAGTTCTGAGCTCGGACCTGGAAGGCGGGGTTCAGTGTGACTCATCAGACTCCTCAACCACCGCCTCCAGGGAGCCCCGGGCCTGAAAACGGGGTCTTCATTTCTGTGGGCTCCAAGTTGGAAACTTGCAGAGGAAGATGAGGGAGCTCACTTCTTCCGGGGTGGAAGGGGTGTGAACGCGAGCCAGCACCCGCGAAGGGTGAAGGTCCCCGGTTCAGGAAGCCCTCTCCCCAAACCTCTAGC

>chr2(+):6126181-6126400

GTAGAAACCCAGAGCCCAGACACCAGCACTGGGGGTCTCAGGGAGGCTACAGCTCAAGGGCTGGGGGACATGGGTGCTCTGGGCTCACTGTAGGGAGAGAGATTCCCACAGTTGAAGGCACCCAGCTCCTCCAGGGCACCAGCCTTGGTTTGAGCTATGCTGGATGGGGCGGAGGCTGAACCTTGGTCTCACAATGGAATTAAGATTGAATGGTGGGGTG

>chr2(-):127129210-127129429

CACTGAGATGTACCTGGGTGTGGTGGCCTCCTGAGCCCTGCTGACAGGGGAGATGGTCTGCCTGGACAGGGGACTGGCCAGGGCTGTCTTCAGAGCAGGCGGCACCACCCACGGGGGCCATGGCTGGGAATGTGTCCACTGCTGCTGGGCAGCCCCAGCCAAGCCCAAGGGAGGCAGAACAGTGGCCTCCGGTCAGTGTCCCTTTGCCCAGCTCCAGACC

>chr2(-):767947-768166

GCAGCCTTAGCAGGATCCTGGACCCTGGTGTGCACACTGCACTCGGGGTCACTCCTACCCTTTATCGGGTGATTTGTGGATGCCCAGCTTCACCCAAGGCTGTCCTGTGGCTGCAGGATGCCTGCTGTCCCCCTCAGCCTGTGGGGCCCAGGAGGTCAGAAGGTGCTGGCTGATGTATCCCTGTGATCAGGGCTCTGTGGGGAATCTCTGGGGGTCTCAG

>chr2(-):130619433-130619697

CACAGAAAGCTGGGGGCTGGAGGGACAGTTCGGAGGGCAGCAGGGCCTCCCCGGCCTCACTGTCCGCATCTGTCCTGTGGGAGCTCGCGGGCCTCGCTATGGCCCAGGCACCCACAGCCTCACCAGGGCTGCCTGGGTCACCGGGCTCTCCAGGAGGCAGGCAGGGGCCCTGGGGTCCAGCGCTGCCCCCATCTCAACTCTTTCCTTCATCAGGGCCCACGAGGGGCCCTGTGGACTGGACCCAGGCCCGCCCATTCCCAGGGAG

>chr2(+):23834014-23834233

AAACCCCAGCTGTCCCCTCCGGGAAGGCTCTGTGCACCTTCTCCATGCTGGTGTGGGCGCTGTAGCACTCCCTGGCCAGTACCCAGGACACCTGAGCTCCCCACCAGCTTCCACACGCAGCAAGCACCGCATGTGCTTGCTCCCCACTGGGCCTGGTCCACAGCTGCACACGCCAACACATGCACCTGGTCCAGGGCCCCAGCCATCCCCTGCCCTCCAC

>chr2(-):177729844-177730063

CAGTGGAAACCCCGAGTTGCCATCCGCGGGCCTGCGCGCAGTAGCGGAGCCCTCGCCCCGCGCAGTCCGGCGGAGGTGAGCCGGCCGCGCTCGGGAGGGTTGCCCAGAACGGCTCCAGCGCTCGGCCCAGGAGCGCCGCAGCCGGGAGCACTGCGGGAGACCGAATTCCGAAAGGAACGGCTGCCTGGCGGCCTCCGCTCGGGGCTGCCAAACCCATGTC

>chr3(-):127099640-127100089

CGCTGCCTCTCCTTAGAGCCCCCTGCTTTCTCCAAAGGAACAGGCGAGGTGGCTGGGCTTCTCCACAGCCTTCCCTGGGGGCTCCCCTGTGGGTCTTTCTGCGGCTCAGAGGTGGGAGGCCCCAGCACCTCTCCGCCTGGGCGGGGCAGGCAATAGGAGCAGAGGACATCCTCCTGACCTGTAGGGCGTCTGAGGCCACCTTCCAGGGGACCTGCCCCCGCCTGCGCAGCAGCCTCGGGGGATGCGGGGGCTGCACCTGTAGAAAGGCTGACAACCCTGGCCAGGGCGTGAGGAGGCTGAGCGGGGCCCAGAGGTGTGGGCCAGGAGAGAGGACACCCGCTGTCGGGGGAGGGGAGCCCTGTGTCCAGGAGGGAGGCCGCTGCCAAGACCTGTGCCGAGGGGTGGCTAGGACACACTTGGACTCTGCACACATGGCCCGCAGGGGAACCC

>chr3(-):135339385-135339742

ATAGAGATGGGGCCAGAAGATTCTCCCAGGAAGTTCCAGCCTCAGCACCCAAGGCCGTCCTCAGCTGGGGTTGGAAAACCTGTCACACTCAGTGCCTGCCCTCCGGGGGAAGGCTGGGGTAATCTCCATGCAGAGAGAGCCCAGGCCAGGAGGCAGAGCAGGAGGAGCCACCCTTCCCTCAGCCACCGCTCCCAGGGACTGTGTGGGATTCACTCCGACTTCACCTGGAAGCCAGCCAGATAGCACTGGAGGCTCTGGGAGCTCAGTGGAAGAGGACTCCTCTTGCTGAAGGTCTAGAGAGTGGATCTGGCCTGACTCACTGCCTTGGGGTGACACTGACAAGCCTGGCACAGCATCC

>chr3(-):49585162-49585427

CTCTCATCAGTCCCCTGACCCAGCCCAACTCCAACTCATTCTGTGCTTCTGCCCTTGCTGTGCTCCCTCCTCCCTGGAGCCTCAAGAGCTCTTTCTGCCCTGCAACCCCGGTCTCTCTCCCCAGCCTGGGATGCACCTGGTCCTCCCCCAAGCTCCATGCTCCCCTGAGTCCCCTGACACTTCTCTTAGGATTTCCTGCCCCTCCTGCCTGGTCCTGTGCCTCCTGTCCCCAGCCCTGGCAGCAGAGGTGGGGAGGGACTCTCAGA

>chr3(-):127076540-127076759

TGCTGGGTGGGCCAAGGGGGAAGTGATTGATCTGTGGATAGCAGAGAACAGTCAGTTGAGATGTATAGCAGGAGGGGCTGCCCAGGGCAATAGGACTGGAACTGGACATAGACTCCTTTGAAGGCTACCCTCCCTGGATCTGCAGTGTGTGCTTCTCTGCTGACTGGCCCTGAGAACTAGGGATGAACTAACTTCAGGGCTGGTTGGGAGTTCCCCTAGC

>chr3(+):195011646-195011865

CCTCTGTCCTGGCGCCCTGGGCTGGCCTGGGCCAGACACCGCACCCCCAGGACTGTTTTCCCATCTGCAAAACAACTCGGGCCTCTCTAAGCCAACACTACCTCTCACTGCAAAACACAGCCCCCTCCCTCCTCCAGGGGCAGTTGGCCTTAGAAACCAGCCCATCTTTAGGGGAAGAAATCCTTATGGGGCAGGCGGCAGCAGGGCATAGCACAGAGGA

>chr3(-):127414303-127414568

GCAGTGGCTGCCCGGCCTCAGGACAGTTGAGCCTCCAGCCAGGTGCGTGAGGCCGCTCGGTGCCCCAGACCTTGCCCCGCTGCACAAGGGCCAGCATGCCGCTGCCTCCGACCTTCGCCCTGTGCCTCATCTTTGCCCAGCATCCCTCCAGGACTGGGTTCACAGGTGGCCATGGTCCACAACCCTTCCCTGGCCTGCCCCCACCCCAGGCAGGTCAGAAGCTCTGGAGCCCTGCTGTCCCTGTGCTCTGGGCAGGGGTTCAGGCT

>chr3(-):135384914-135385133

CATCCTAGGGAGGTGAGGGAGTGGCCAATGGCCCCACCAGACTAACATGGCACTCAGGAACCGCAGGGGGGCCAAAGGGCCTGAGTGGGTCTAATGAGGGGGACCTGGGAGCACCCAGTGGTGACCTGCATGGGATATAAGCAGGGTTCAGGTGGGGTACAGGGTATCTCAGCAGGTGCCTGTGTGGACAGGAGCCAACAGAGCCAGGGAGTGCCTTCCT

>chr3(+):13527447-13527666

TGGGGGGCCTGGACACTGACTTTCCTAGTATCTCTTGCAACTGGGGACAGATGGGAGACCCACACTGCCCCCACTGTGGCTGCAGCTGCTGTCAAATGGGAGGTGGGAGAGATGACTAAGCAGGCTCAGGGCCGAACCCACTGCTGGGGAAGGAGAGGCACTGTGACCCTTGGAGGGAGCTGGGGAGAAGTCCTGGCGGGGAGTCCGGGGCCCACCATTC

>chr3(-):135410184-135410403

GAACCTGTGCACACTGGCCCTGGGGCACCTACCTCCCCAAGGAGTCAGTGAGCCCTGAGGGGGCTGCAGCACATCGCTGGAGTGGCGCATCCACAGTCCACACAGCTCCTTCACAGCATGCCTTGTGCCCAGCACATGGACATCCCTGTGCCCAGGCCCCACCATGTGGCCAACACGGAAGAGATGTCTCACCTCTGTGGCCTGCATGGAGTATAAATTC

>chr3(-):127124049-127124406

CCCCACCACCTGCCCAGGACCATTTGTGAACCATGGTCTTTGACTACCCTCCCCGCCTTCACAGTGCCTGGTCCTGAGCCCTGAGCGCTGGCCCTCTCTCTGCGCCTCTGTGTGTCCAAGGTGCCGGGCAGCAGGGGCAGCACCAGGGGCAGGTGTGGTGGGGAAGGAGCTGCAGCAGAACTGAGGCTCTCCCACAGCCCGGCTGAGGTCCAGGATCCTCCAGGAGCCACGGTGTCTGGGGAATGGATCCCAGTGTGCAGCATCTGCCCCCCAGGGACAGACAGTTTTGGACTAAGCTGCTCCTGCACAGCCTCTTTGAGCTGCTGGGCCCCAGCAGCCCTGGAAAGGGTGCCCCCTG

>chr3(-):128343191-128343454

CTCCCCCAGACACAGATGGAGTCTCAGCCAGGGCCCCCTTTCCAAGCCCTAGGTGAGTAGGGGCCTGACTGTCTGGCGGGTCTCGAGCCCACCAGCCAGTCAGCAGCAGGGCCCCATACAGTCTTCTGGATGGCACTGTGGGCCATGAGGGAGGCTGGCCACACTGCCTGCCTCGAAGACCAGCACACCTGTAGGTGTGTGTGCATGGGCGTGAGCAGGGGGTCTGGGAACAGCAGTGTGGATGTGTGTGCTCCTGACGCTGTC

>chr3(-):126912803-126913068

ACCTCCCACAGGGCACGGGACCAAAGCTGGGAACTGACTTGTTGGCAGGACAAGGGAATTCTTGAAGGCTCTGGGACCACTCAACAAGTCAGAGGCTGAGGCTGCAGCGTTAGCATGCCCGGAAGTCCCCTTGCCGGGCAGCAGGATTGGGCCCTTGGGTCCTGTTTTCAGCACCGTGGAGTGGACAGCTCCTCAGCTGCTGCCCCCAGCTCTGGGCCCAAGTCGGGTCATTGGCTCCTTAGGGAGAGGAAGCCAGGCACTCCAGC

>chr3(-):126318846-126319178

GAATGCACCTGTGGGAACCTGGGGCTCTGTTTTCTTGTAACCCCTTGGATCACCACCAGCATACCAAGGACGGCCCAGCCAAGTGCAAAGGCAGCCTGGCTGCCTGCACTGGAGAGCTGCTGCCCTGGAGGAACAAGCCTGGGCCTCTCCCAGCCCTGTCAAGGCCCCTTCCTCTGGTGTTTGGGGAAAGGGGTTGGACTCACATCTTCTTGGACAGCTGCCTGACCCCAGAGCTGGTGCCCTGCTCACATTCCCTCCATGGAGATGAGGCCAGGACTCAGGACCACCCTCTGCCTCCAGGGGACACAGCTCCCACCACAGGCTCTGGCACAG

>chr3(-):127013144-127013363

CAGGTGGGCAGCAGAGCCCCCAGGGTTCCCAGAGGCCAAAGGGACATTAGACCCACAGCACAAGGCCTGATGCCAGGTGACCCACATCCTGCCTGCCCTGGCCAGGTGGCAGCCGGACACTGTTTAAATGCCTCTGCCACCTGGCCCTGAAACTTACTGCTCTCATGGGAACCAAGAACTCTCTTGGTGTTTGGGCAGGCATGGCCACCTGCTGGATGTG

>chr3(-):11072823-11073073

CAAAGGGGCTGGTGGGACAAGAATGTAGTGGACACAGGGTGGGGCGTGGCCTGGGGGTGAAGGGCAGATGCCTCAGGAGGCGCTTGGAGGCCGGAGGCTGGTCAGGGCTCGAGGCCAGACATGCAGGCTCCCCGTCTGGGCTTTTGGCTGTGCTGAGATGGGCAGGGCTTGGTGGGGATGCTGCCATATCTGGAAAATAGCTCTGGCAGCCCGTGGGGGCAGCCAGCCACGTTCCCGCATGTGTCCACACC

>chr3(-):99134374-99134593

AGGAAAGGAAAATGGGGCAGGGCCTGTATGAAAATGAGGAATTCATTGAAGGAGAGTCCAGGAGGAGCAAGACAGGCTTGACATGCCCACTGCAGGGCATCTGGTTGCTTTGAGGGGGCTGCTTGGAGGAGCTCAGGCAAGTTCGGCCCTCAGCCTCCTGGCTTCTTCAAGTCCTGCAGTCCTGGGGTCCAGGTCCCAGGACACAAGACCACTCAAAGCT

>chr3(+):126061474-126061783

CTCACTCACAAGCTACCCCTCAGATGGAAAACACCCCATCTCCTCTGGCACTTTCCTTCCCAGGCTGAGGACTTACATCCTCCAACCTTCACTAACTTCTGGGGAGGTCAGCCAGGTTCAAGGGCACTGGACAAGGCAAGAATGACATGACTGTATAGCTCCCCTCACCCCCTGAGCCGTCCTCTGCCCAGTGTTTGTCAAGCTTCCTTCCTGCCTCCTAAGAGATCGGGAGTGGGCCAGGAAGGCGGCCCTGCCCCAGCCCTGCTGCTATCAACTGGCTCAAAGGTGGGGAGGCCACTGGTCTGACCAG

>chr3(-):142208287-142208506

GAAAGTGACTCTCCCTCCCCAGTGTCCTGGAGACAGTGTGGGGACAGCGTGGGTGCCGGAGGAGGGGCTGAGAGTGGAGGTGGCCGGACCCTGCCCTCTGTCCCGAGGGGATGTGGAGGGCCCCCCTCTGGGTCCCTTGCCCGCCCAGCTTGGACAGCTCCTGGTCCACCTCGGAGTCCCAGGGGACATGGAGAGGGCCTGCAAGGAGAGGCTGCTTTGC

>chr3(-):12935424-12935735

CCTGAAGCTGTGAGGGGGGAGGGAGATCTGATTCCAGGTTCTAGGGGCTGCCCTCTCATCCCCAGCCTGTCTCCTGGAACCTGCATTCTCATCTCCAAAACCCACTCAGGTTCCTGGGCTCTTTGAGTGAGAGAGGGTCTCACAGCAGCCGTGGAGGGTGTGGCCTGGCCCCTGCAGGCTGACGGTACTCTTTATGTTTGGGAGCGAGGCCTGGGCTGGGGAGGCTGGAGCCATCACCTCCCTGGGGCCGGAAAGACTGCCTTTGACAGGACAAGGGCTGCCTTGGCTGTGTGGCCCTGGGTTCACAGGACA

>chr3(-):128799136-128799354

GAGCAGCCTCCACCCCGCAGCCTGCGGCCAGCCCTCCCCAAGCGACGCTGGGTCCCCAGCGGGACGGGAATGGCAGCTCAGGCTCCGGGCTGCTGTTGTCGGGGGAAAAACGGCTCGGCGCTGTGGCCGCTCCCAGGGGCCAGGCTGGGCCCTGGTGAGGCAGGCGGGTGGTTGTGGAGGGCAGAGGCTGGGAGCCCGACCCCTGAGGAAAAAGAGACA

>chr3(+):14340501-14340788

CAGCATGGAGACTGTGGGTGACCGGGAGCGGGGAGATGCATTCTGCCCAAGAAAGGAAAGGAAGAGAATGGTGCCCTTCTCAAATGGTGCATTGGAGGGGGCCAGGCTAAGAAGAAGGAAGAGCCGGCCAGGCAGAGAACACAGCAGATAGAAAGGCACAGTGGCTGGGAAGGATACAGCGTGATGGGAGGAAGGGGTCATCCCATAGGGCTGAGTGCTAGTTCAAGGGCAGAACTGGGGTAAGGAAGTTGAGGCAGGGGCATGAGGAGCATCATATGTTGGAGCCAC

>chr3(+):126961347-126961566

CATGGGTTTCTCCTCATGAACTCACTTCCTGCTAAGGATGTCTCAAGGGGACTTCTCAGCACCCAGGCAAAGCCCTGGGCACTCACCATCTCCCTCATGGCTCCAGGAGGGCCTCTCTGCTCATAGCACCAGGAAAACAGCAGAGCTTCTGCCCTCCCCAAATGCCAGCACCCCACTAAGGTGGGACAGAAGTGGCTCCAGGAGCAGCAGAGTGAGTTCC

>chr3(-):126834290-126834601

ACTCCCAGGGTGTTACGGGTGGCCCAGCACCCACTGCGCATGGTTGGCTGCTGTTCTGGTTGCCCAGTGGCCAGGTCACATCGGTGGTCACATAACCGGGACTACGTGCCTGCCCTGGTCTAAAACAGACACCTAGTCATCATCCCACTCATAAACACCCCGGCCCAAGCCTCTGAACCCAGGTCTACCTGCAGCCCTGCCTAGGGCCCCAGCTGTGCTCACCCGGCTCTGCAGGAAGCCCCATCAGCATCACCTACTTGAAGGGAGGATGGACAGGGACCCTGTAGCCCCCAGCCAGGCAGGGGACGTGCT

>chr3(-):14345332-14345551

GAAAGCCCCGGCCTCTGCTGAGCCCTAAGAGGAAGCGGGGGCCCAGGACACATGGGGAGGATGTGGTGAGTCTCCAGCCTAGACGATGGTCAGGGAGGTCAGAAGGGGCTCCTGGAGAAATTCTATCCCAAGACCTGCTGTCTCCTTGCTGACCGTTGGAGCTTTCCAGAAATTCAGAGTCTGCATTTGGCTGAGGTCTGGGTCTTTAACGATTAAATTG

>chr3(+):133304544-133304761

GTCACAGCCAGCAGCTCCCAGGTTTGCATACATTTCCTCAATTAAAGACAGGAGAGCTAGACCAACTGGAATCTCCTACTTGCAATTCCAAATCCCTAAGGAAGGATCTGCCTGGCCCATGATGGAGGGGTTCCTTTTCCTGATACCCCTCTACTATGTGGATTGCTAGGAGGAGGTCAGTTCCCACAGGAAACAGGAAAGGTGGCTGAGGAGCACAC

>chr3(+):13678900-13679309

TTCTGCCGTAGACTGTGGTGAGCCTGGAGTCCGAGTCTCCCCAGTCTCCTCCCTGCCTGCTCCAGCTCACAGGGAGGTTTCCCAGACTTGGGGGTGGTGGAGACGAGGAAGGAGGCTCAGGGGGAAGCTGGCTGGGCTCTGGGTGCTCAGGCGGGCTGTGGGCTCTGATAGCAGGCAGGTCTTGCTCTGCCACTCCATGGGAGTGGTGGCTGGTGGAGATCTTCCCTGGGTGGGGCACCGGAGGATCAGAGGGACCTGGAGGGGCACACAGGGGCTGTAGGCACATGCAGGAGAGTTGCAGGGCTGACGGCCTGGTGGCCTGTGGGGTACTCAGCAGACCAGGCCAGACTGAATGGCTCCCACCTCAGGTGCTCTGGGGGAAAGGTGGGCATACCAGCCTGGCCAAGATT

>chr3(-):126856052-126856271

ACGGATGTAGCAGGGACCCCACATCTGGGGCCACACCTAGTCTTTTGTTTCTAAACTGCCCTGCCAAGAGAGAGAGGGCCCCTGGGACTGGGGTGAGCATAGACTGGGCAGGCAAACTCAGGCTGGGGCCTTGGGGTGACTTTGTGGGCAGATGGAGCCCCCCGAGGCCTGTGCTTGGAGCCCAGAGGCACCGCCTGCCCTGGGGACTTCATGCAGCCCT

>chr3(+):13041820-13042039

AGTTGTAGGAAGAGGGGTGACCAGCTCCTGCAGTCTCAGGGCCTGACTCCATGCCCTGGCTTTCCACCGATGGGCGGGGTAGCCCTGGAAAAAGTCCCCTCCCTCTCTGGACTCATGCTGTCTGGACCTGGGCTTCCTGCAAGGGGGCCACCTGCCCAAGGCTGGATGACGGGCCTAGGGCACATCTAAGGAACAAGGACAGGACAGAGGCAAAGCCACA

>chr3(+):126840687-126840906

GACAGGGGACAGAGAGCATACCTGCAAACCTGAGATGTGGGTTAATGGTTCCGAGATAGGCTCTGAGGCCAACTGGCCCTGGCTATAGTCACAGCCACAGCACGGGGGCCAGTTGCAGCATCAGTTATACAGTAATGCTGTGTCACAGCTGCCCCAAGCGCCAGGCCTCATAGTTGGCATTTACCCTCACCTCGGGCCCTGGCTGGGTGGCCCATCCTTC

>chr3(+):106655246-106655465

CTGCCAGAGCTGCCCCCACTTCTGCATGTGTGCCCACCGCCAACCCCTGCAGCTGCATGTGTGCATGATGCCAGCCCCAGCCTCCAAAGCTTCGCATGCACATGCATCTGGCCCGCACAGCCACGCATGCACTGCACTTGGACCAACCCTTGCCACTGGCAGCAGCCCTCACCATCATGTATTTGCCTGCAGCCAGCCCCTGCCACCAGTGCATGTGCCT

>chr3(-):126885841-126886130

CGGGGCAAGGGTGCAGCAAGGTCCCTGGGAGGCCAATCTGCCAACTTCAGGCACCCAGGCAGCAAGAGAAAACCTCTGTCCGGGCTCCCCTCTGGGGTTGGGGGCCAGGCTGGTGAAGAACACAGGCCCTCCCCAGGTAGGACCCAGCCGCTGGAGGAGGACAGTATGTTTTCGTTTTTGCTGCTGCCCCTCTGGAAACCACAGTCAGCTCTGCCTTCCCACGGAAAGAAGCAGGGAAGAGGGACTGGAGCCTGCCTTCGGGGAGCCCAGCTCGGTGCCTGGCACTGCAA

>chr3(-):138923489-138923754

TCCAGCAGCCTCAGCCACCAGGCTGACTCTAAGGAAGCAGGGCCAAGGTCAGGGCCGAGGGGCCTCCCAGCTGTGGGTGCTCCCCTGGGCTGCAGTCCAGAAGCCCCCAGACTACACATGAGGCCTCCTGGGTCTTACTCACTGGGCTAACTGCTGGGTTGTCCTGGCCTCGACTTCTCAGATGGAGCTGGGAAAGGCAGCTGGAGGAAGAGAACTGGAGGTGGTGTCCAAGGCCACAGCTCAGGCTGCCCCTGGAGGAGGCCCAG

>chr3(-):127405633-127405852

AAAGGGAGGAAGGTGGCTGTGACCAGAGCGAGGGGGGGTCCTGATGCAGCTGGGGAGTCAGGCTCGAGTGCATGCTGTGCATGTTCTCTGCCTCTCTTCCTGGGGGGCAGGCGAGTCTCCAGAGCCTGGAGGACAAGTTAGCCTTGCGAAGGATAGCAGAGGACCCAGCCCAGGGGTCCCCAAAATGGAGCACCAACCCTCCATGCAGGCCAAGCCACTG

>chr3(-):135384822-135385041

AATGAGGGGGACCTGGGAGCACCCAGTGGTGACCTGCATGGGATATAAGCAGGGTTCAGGTGGGGTACAGGGTATCTCAGCAGGTGCCTGTGTGGACAGGAGCCAACAGAGCCAGGGAGTGCCTTCCTCCACAGCTTAACATGATGGGCCTACCTGTAACCCAGATAGCACTTCTGCTACCCCAGCGAAACAGGGGCTCAAACTGGGAATGAACAGGAGT

>chr3(+):13679090-13679309

CACTCCATGGGAGTGGTGGCTGGTGGAGATCTTCCCTGGGTGGGGCACCGGAGGATCAGAGGGACCTGGAGGGGCACACAGGGGCTGTAGGCACATGCAGGAGAGTTGCAGGGCTGACGGCCTGGTGGCCTGTGGGGTACTCAGCAGACCAGGCCAGACTGAATGGCTCCCACCTCAGGTGCTCTGGGGGAAAGGTGGGCATACCAGCCTGGCCAAGATT

>chr3(+):126889548-126889767

AGGACAAGACCCTGGGGCGATGGCCTCCTGATTGCCCCGTGGGTCATCTGGGTCCTGCCCCATCCTGGGCCCCGCAGACCAGGCTCCTGGCCATCTGCCTGACCCCAGGCCTGGAGCCTGGTTCCCATATCCTGGGCACATCACAGCTTCAGCAGACACCATCAGACCGTGGCAAGGGTCCCTCTGTGAACCCCATGGACACCCCTCTTGATCCTCTTTC

>chr3(+):99134374-99134593

AGCTTTGAGTGGTCTTGTGTCCTGGGACCTGGACCCCAGGACTGCAGGACTTGAAGAAGCCAGGAGGCTGAGGGCCGAACTTGCCTGAGCTCCTCCAAGCAGCCCCCTCAAAGCAACCAGATGCCCTGCAGTGGGCATGTCAAGCCTGTCTTGCTCCTCCTGGACTCTCCTTCAATGAATTCCTCATTTTCATACAGGCCCTGCCCCATTTTCCTTTCCT

>chr3(-):126930829-126931048

TGCCCTGCTTGGAGCCCCAGCAGCTTCCCTCAGTAGATTGCATCTGCCACACTCCTGCCCGCTCTGGCTTTCAGGAAATCAGAGAGCAGGATAGAGGGTGGGCTCAGGTTCTTGATTCTGTCGCCCAATTCCTCCCTGCAGGCCTGTGGGCTGGTGGCAACTGCATTCCTAATCAACTGTCCCTCTCCTAAAGCTGCAAGTGTCCCTGGGCCCTGGAACA

>chr4(+):7869215-7869489

AACCAGTGTCTGCAGGTGGGCAGCACAGACCCTCTCAGCAGGAGTAGCCACATGGAAACCCACGCCAGCCCAAGCTCAGTTCTAACAAAAGCTGGGGAAAAGCTGGGGAGACGCAGCCATCCCTGCACACATGGGGCGGGTGGCGGTCTTTGACTTCCCCACCTGTCCCAGCCACACCCAGAGTCTTTAGGCAGGGAAACGTGGCATGAGTCTGGGACTGCTGGAATGATTGCCAAGCACTGCCATTTCCAACGGGGCTCGGTGGCAGTGGCCCT

>chr4(-):154308612-154308847

TGTTCAGCAAACGAGATCTGCTGGCCCTGTTTGTGGCGGGGCCGTGCCATGAGGGATTAATGTGGTGGAGCTGGGAGGTCACACGTGGAAGGGGAGCCTGTTCAGCTGACGGGGGTTGTGGCAGACCCTGGTTCCCAAGGGCCTAGGAAGCTCCTGCCATCCTCTCCTCCAGCGCCGCCTCCCTCCACCTTCCTCCCCAGGTCTCGGGCCTCTGCCTTGCCCGGCAGTTTCTGGCC

>chr4(-):44977382-44977625

GTTGGACTGCTCCGTTGCCCTAGCCTCCCTTGTGGGTGACATTACACAGTCGCCCTCCCAGGCTGCCTCCAAGGACGCCTGGTTCTCCCCCATTCTCCACTCTTTCCATGACAGTTTGGGGGAGGCGGCGCAGTGGCTGCGCAGAAAGCAGCAGCCCAGTGTGGGAGGTGGCACCAGTAGTGCTGAGGGACGGGGCGTGGAGACAGCTGTGAACCCAAGACAAGGAGCCCCCAGCCCACCCTCA

>chr4(-):154394328-154394547

GCTGGCTTCCCATCGATCCAGCTTTTGAGCCCACAGATGGCATCTTCAGGGAGACGTCACGGAACCATGGATGGTGTTGCCACATGCATGCTCTGACAACCTCAACCAAATGAGAAGGAGCAGGGGCTGTCCTCAGTGTGTTGACTCTTCCCTGCTGGTGGGGTCAGGGACATCCTGGCCACCCCAGCAGAGGCCTGCCTCGTTCTCACACTCTGAGATT

>chr4(-):155320501-155320766

ATAGGAAGCACTGCCCAAAGGGAAGACAAATATGGAGGAACAAAAATGGAAACTTTATGCTCCATCAGCTCCTCCCATGTAGAGACCGTTCCCACACCAAAAACCGTGAGAGATCCTGCTTGACTTTTCCCCGGGACAGGGTGGTCCCCCGCAGCCCCCTAGCTTCCACGTTGTGTTGGCGACAATGCCAACATCTTGGAGAGGAGTGGGGTCTGTGGAAGGAAAGGAGCGGCACTCCTAGGCCCACCGTCCCGTTTCTTTTGAAC

>chr4(-):26053629-26053848

ACTGAGGCAGTGGGTTCCCTGCTGATGGCTCTCCAGGTTCTAATCTCTGTCTGTGTTGGGCTTCCAGGGCTAGGCGTCCCGTCACTGACTGCTGGACCCCCTGGACTCCAGGCTCTGCCCTTACCTGGTGCCAGCTGTCCCCATGGCTACCACTGGGCTGCCTCTTGGACTTGCCCCAGGCCTGGTTCACAAGTCTGGCCTCTGCTTCTTTCCAGCCCCT

>chr4(-):74833071-74833290

GAGGGCAGGGTCAGGGAAGCCAGTCACCGGCACAGCTTTCGCCCAACAGCCTCCAAGAAGTCAGACTCACAAGACCTTCTTCCTTGTGTACACATCCCCTTCTTGTGAAAGGAGTGGCTGGACCGGTGGGTAGTCCCTCCCTACCGATTGCCCCAGCTCTGGCTCCTCGCATGTCCAGTGCCCCTCCTGCTACCCGCTAAATACCTCAGCTCCCTGTCAC

>chr4(-):186615540-186615779

TGGCAGGCGTGAGCGGCTGCCGGAAGTCTCCCATTTAGCATTTGTTGATTTGCTGTGTGAACTCCTCCAGGGCTGAGGAGGAGGGCTGGCTTCCTCCTGTCACTATCAAGGAGGATCTGGTCAGACTCCTGTGATTAGGGCTGGCAGCCCTTCTGTGGGACACCTGCTGCCCGTGGCTTGTATGATCATTGTCAGGAGGCATTGGCCGTGTCTATGCCCCCTTTACGCCTTTTTCTACCT

>chr4(-):27209272-27209491

TTCCCACAGACCTCTGCGTGAACAGCCCTGTTCCCCTCCCTGACTGCATTTCCCACCGCTGCGCTGAGCTGGGCTCACCATGGGGAAAAATGGCCCAACGGTTAGCCCTACCCCTCAGTGGGGCCATCTTTACGTTGACACAATGCCAACCACAACATTCCCATCCCTGAGCAAGTTTATGCAGCAACTGTAACACTTGAACCCTGGCTGGGAGAGGTCC

>chr4(-):153533732-153533951

GTCTGGGGTTTGGACAGGAGGTGAGATCACTCTCCCAGAGGCACCATGGGGCAGAAGTGGAGAGCCAGGCACTGTCCCCAGCTGGCCTATCCTGGCCACTTCCTTCCCAGGCCCATGTGGGTCACTGGGCTCCTCAGAGTTGGTGTGACCCTGGGTTCCATGGGAAAGGTCTGCTCCTCACTCCAGCCCAGGCCTCTTTCTGATCCGTAGGCAACGGCTG

>chr4(-):191627340-191627560

AGGAAGCCAGGGATCTGTGTAGGGCTGCAGTTGGGTGCACATTAGTTTTGTGACAGGATGAGAGCTGCAGTGGTTTTATTAATCGTGATAGCCTGGGCTGGTTGTAGCTTCAGGTGAGGGGAGGGAGTCAGCAGTGGTGGTCCCGGAGACATCCATGTGCCCAGCCCTGGCCTTCCTGCCCTCAGGCACAGCAAAAGGCACCGCCACAGGCCCCGACTTCC

>chr4(+):184944120-184944367

CTGACTCTACCTAGCAGGGGACTTCTGCTTTGCGGGACACGGCTGACTACAGCCCCCTGACCCCCAGCCCACGTGTCCTGACGGCCCCCTCCTCTCTCCCCTCCACACTCTCCCCACCACTGGAGCCACCCAGTCACCCCTCCAGCGTCTTGAGGATGGAGACATCTCCGGGCCATTGTTCCTCCTTGAATCACTAACGCGGTAGCATTTCTACACGGAATGCCACTTTCTCTGGCCCCAGCCCGTTG

>chr4(-):173280383-173280602

GACCCACAGGCTGTCGCGTGTGCTCTCCTGGGATGTGGAGGTCACCAGCCAACTGGCTGTAGGGTTGGGTGCAGGTATGTGGTGGTATATCCAGGAGGCTGGCCACTGCCACATTGGCTGTTAGGCATGTTCAGGGCAGGTGCAAAGGGACTAGCGGGGGTGGGGTGGCTAGTTCACTTCTGAATCAGCTTTCAGGCCAAGTGCAGGTGCATTCAGTGGG

>chr4(-):184464583-184464832

CCTCTCTGTGGAGCCCAGGAGAAGGGGCAGTGCTCTGGAGGGGAAGTGGGGGCACGGCTGCCAGGAGGAACTCAGCTCAGGCTGGCGCTGGGGTGTGGTCGAGGCAGAGGGAGGACTTCACGGAGAGGGTGTCCTCTCTGAGGCCTGGAGGAGGCATGAATGAGCAAAGAAGGAGGTGGGTGGTGACTGCCAGAGGAGAGCAGGGAAGGGCTCTGCAGCCTGGAGGCTGGAGGGGAAACTTGCCCAAATC

>chr4(-):71626199-71626418

AAGCCACACCATCAAGAGGAACCTCCCCCCAAGTTGGCCTCACACAGGAACCCTGATTCCCAAGGGAGCCACGGGTCAGCCCCGACCCTGCACAGGCTGGGAGGAAGAAGCAGGCAGCCTTCCTGTTTCCTCTTACTCTGTGCATTCCGACCTGAGACCTGGCTAGCCTACAACAGCCCTCCTAGAGTAGGCTGGAACCCCCTACCCTGATTCTGCTCCC

>chr4(-):109659171-109659390

GCCTGCCTGCTCGCCATTCCCTCTGGGGTCCAGTACTCTCTAGGAGGCATTCAGAAAAGGAGCCCTGACCCACCCTCTGTGGCCTGCTTCCCAGGCCCCCGTCTCCCACTTCCCAGCATTAGTGCCTCCTGGCCCAGGGCCAAACAGCAATGGTTGCAGAAGGAGAAGTGAGGCTGCATGCCTATGGACTGGGAGACTGGTGGACAGTGGAGAGAAATTC

>chr4(+):134668651-134668870

ATTAAGCCTAGGGCTGCATCCAGGCTTGTCCTCTATTTTGTGTCTGAGATTGGAATCCCCTCAAAACCCTACATCCTTGATGGGATTGCAGATCACCAACCCAGATCACAGCTTCCTTTAGGGAAATTTTTTTTAGGACCCTTGATACATTTTTGGGCTACAAGATGAGCCTCAATTAGAATCATCTCTAACTTTCAAAAGATTCTGATAAAATAAATTG

>chr4(-):23508651-23508870

CTGGAGGAAGGAGGAAGCTAGAAAGACAGCTACTTTGTGCAGGAAGGCTGGAGAGGAGAGGACCCTCCGGGGACAGACTTGGAATTGGCAGCTTTGAGCAGGGAGTGGGAAGAGTCCCTGGGGCCTGCTCTGAGGAAAAACAGGGGAGTGCCAAGCCTGGGCTGCACAGGGTTCGAGGCTTCAAAGGGCGAAGCCTGTGTGCTGACTTGACCTATCTTTG

>chr4(+):601209-601428

GGCTGGAGGCTGTGGTGTGCCCCACTAACTGTGCAAAGTCTTCTCTCGGGGACAGGCCCCAGAAACCATGAGGAGCTCCTCCCCCAACATGGGTGGAGAAGTCGGACTGTGTGGCCCGAGGCATGGACTGGCCACCATGGTGCCTGCTCAGACCTCCCCTCTAGAGACCCACACTGAGGAGTGGGGCCACGGCCTCCAGGCATCCCCACCACTCGCTCAG

>chr4(-):184944801-184945027

CTATTCCTCCTCCTCCCCAGCCAGCCCCTGCCCGAGGCTGGGGACTCACAGGCTGTGTCCCAGACCTTGCCCTCACGGTCTGGTCCACCTGCCAATGCGCAGCACAGGACTCAGCATGGCCGGGGAACCACATGTTGCCTGGAGAGAGGCACAGACACAGTGGGAGAACTGGAGGAGGGGACAGAATGAAGCTGGGTCTGATTTCTCACATAGAAAATGGGGTTTGT

>chr4(+):7034113-7034332

GAGCCCCTGACCCCGCTTTCATCTCTGACCCCTCTACCCCTGGAGCCCCTGACCCGCGAAACCCCCTTTCATCTCGGAGCCCTGACCTCCTTGGACCTCCACCCACGCGAACCGTCTGTCCCTGGGCTAGGGAATCTCTCTGCCTCCGCCTTGGAGCCTCCCATTACCGGGCCGCGAAGACCCTGGCCCGGGGACTGTCTCGGGGCCGGGGTAGCTGCCT

>chr4(+):188674989-188675392

TGCGAGGTGGGACCAGTGGCACAGTGCAGGTGCAGCTGCCTGAACAGACCCAGATGGCCTCTGTGCAGGCTGCGGGGCTGTTGAAGCCCTGGGGGCAAGCAGGGTTTTTGCACAGAGAGAGGCTGGCGGGATGCCTATGGATGTTCCATGGATGGGTGTTGAGTCGGAGCAGGTGAAGGGCCCTCCTTGCAGGAGGCAGGGGTCGGGGTACAGTGATGGGCGTGGAAGGCTGACTGGAAGAGGGGAAGTCCATAGGTTGGGGAGGGATGACTGTGACCTGGCCTGGAAAGGGGTACTGGAACCTAAGAGAGAGAACAAGGTTGTTTGAGTAAGGGTGTTGGTGGAGTGACCCTCCACGGGGTATGAACCTGAGAATGGGGTGAGGAGGGCATCTTGGGTGGAAC

>chr4(-):153424967-153425186

AGGGACTCAACTGCCTCCCATGGAAACTCTCTCCTCTCCCTAGCTTCTCACAGGTGACCCTGACGACCCACCACCCCAGCTGTTCTCCATCACCTGACAGCTGAGCCTCCAAGATGCTGGCGAGTTCTGCCTGTGCATGGCTTTGACCAGCTCCAGCCAGAAAGAGGAACTTTCAGAGTCCAGGTTTGGGGCCTGTGGATGAGTTACCTGTTACCAAGAA

>chr4(-):111483395-111483706

TGGTTCTCACTCCTCCACTTGCCCCTGGAGATGCCACACCAAAAGGCGTTGGCTGGCTTCTTCAGGATCACCTCTCTGACCCTCCATGCTTCTCAGGGATGGCCTTGGTGGAGATGTACTTCCTGCTCAGGAGCTGGTACCTGGGGTCGGCCAGCACCTTGAAAGTGGGCTCGTCCTCACTGGATATCCTTGCCCCTTGCATATGAGCCCAAGGACGTGGCCATCAGTTCCTGCTACTGCTTGCTCTCATCACTGTGGCCAGCCTTTGTGGCCAGTGGGTCCTGCGACAACTGCTGGGATGACTTGGGCTTC

>chr4(-):154421962-154422212

GCCAAGCAAGGAAGGGGCCGAAGAGGTGGGAGCATGCGGACCACAGCAGGGCTGGGTGTGGTGAGAAGGGGCTATGCTCCACGGGGTGGGGGCATGGAGAGAGCAGAGCGGAGCAGGGTGTGGGGACAGCTGGATGGAAGCTATAGGACCTCTCCTGGGACCCCGCCAGTAGGCACGTGAGGCCTGCTGCACCCAGTTCTTCTGTCCTGAGAGGGCTGCGGGCCATGTGCAGGAGCTGTGCTTCCTGGCAC

>chr4(-):151364564-151364829

AGACCCTGACACCCCAGGTTGGGCTGCCTCCTGGCATCCCCAGGATGCTGTCCGCATCCCCCTGGTACTTGGATACCGCACTGGGCAGCCTCCCATGCATCACTCCTCCTTGACCTTCTTGGGCTCTGATGCCCCTCAAGGTCAACTTGAAGTCCCACTCCTATGCAGTGCCCTTCTCACCTCACCCAGGCTCCAACATACCCTGCTGGCTGCCACTGCTGCCTCTTGACCTGAATGCCTCCCTTGCCCCAGCTCAGGGTCTAACG

>chr4(-):184420892-184421249

GCCGGGGAGCCTCACAGGATGCCCAAGGGCCCGTGTTCCAGAACATGGCCTGTGGGACCGCCCTGGACCAGCACTGCGGAGATGGCCCCAGGGACGCATCAGCGGCCCTGCACCATTGCTGGTGCCCTGCACAGGTGTGCCCGCTGCATATGGTCTTGGGTCTGACCTGGGCTGAGGGCCAGGAGGAATCATCCTCAGTGTGGCCCTGACCCGCAGAAGCTGGGCTCCAGGCAGCCTGGAACCAACCTCCCCAGTAAAGCCCGGAGCCACGCCACGAACCCCCTGCACTCAGGGCCCCCTCCAAGCACTCCATGTGCTCTAAGCAGCTTCATCTGACCACAGCCCCTGGGCCGCAACG

>chr4(-):100216201-100216466

CAAGGGAGGCTGAGAGTGTAAGAGCTCAAGAGGAAAGAGTCCGTACAGGGGTGGGATCCATGCAGCTCAGAATAGACTGTCAGAACCTACACAAGATGTGGAGAGGGTCCATGCAAGGAAATAGCTCAGCACTGAGGCTGACTGCCAGAGCCTAACACACACCTCACCTTGCTCAGGTTCCAACTCCCACCCTAGACTGCCTCTCTTTGGGAATTCCCTTTTCATCTTGGTCAGGCTGTAATACTCAGTATCAAGAGGGTGTCTCC

>chr4(-):52991721-52991940

GCCCCCGATCACATGTGTACCCCATGCACACGCACACCATACACATGTGTGAACCACAGTAACCCAGCTGCCTAGGGCTTCCTTACAGTCAGTCCTCTGCCTTCCCAGAGTTGGATGCTCAGTGACTCCTTTCTGCACCAGTAGCCTGTTGATCTTGCAGATCCTAAGCAGCACTGTAACCATCATATGATCTTCCCTTCCTCCCTGCCCAAATTCAATT

>chr4(-):185486945-185487210

GAACCTTCACCCTCCTGATCTTGCATCTGCCTATACCATCCAACAACCATCTGAACCTTCACCCTCCTGATTTTGCATCTGCCTACACCATCCAGCAACCATCGGAACCTTCACCCTCCTGATCTTGCATCTGCCTACACCATCCAGCAACCATCTGAACCTTCGCCCTCTGGAGGACCCTTCTCAGAGCCCTCGTGTCCACCCTGTTATGTGGGCCTCCATCTCTGCAGACTCATAAGCTGGGGCCAGGAAAGGGTTCCCTCCCT

>chr5(+):69936089-69936308

TTGTGAAGGAGGCAGTGGATCTCCAGAGCCCAGCAGCAGGGAGGCTACTTTCCGGGAGAGGGCAGCAGCACAGGAGAAGGCCCTGCCGCCTACAGGGGTCCTACTCCACTCCGGGACCCTGCCCATCTGTGGCCATCCTCCCACCAGCAGCCTGTCACCTCCCATCCCTCCCGCTGGTGACAGGCACCGGGGTACCGTGCCCAGCCCCCGGGCACCTCCA

>chr5(+):10296587-10296806

AGATGCAGCCCACACACGGCACTGCCATGGCCCAGACAGCCCATGGAGGGGGGAGCCCCACCTACTACAGCTGGCCACTGATGGCCAGGCCTCCATGGTGCCAGGGAAGAAGATGACAAGGACTGACAATTCTTCCCCCACCAGCGCAGACACAGACAGCCAGGGTTGCTCAGCACTGCACGCCATGCAGCCCCAGACAAAGGGAAGAAGACCGCAAACC

>chr5(+):172707906-172708125

CTGCTGGACTCTGGCTCCCCTCATTTTATTGCTCCCCTCCCTACTCGCTGCCCTTTTCTCTCGTGGCTCCTGCTTCCTTGTGGCCCACTCATGGCTCGTCATGGCCCCCACCTCCCTCTACTCTGGAACTTCATGCCTTTGCTCCTGCTGCTTCCAGCTCGAGGCCTCCGCACCTCCAATTCCTCCAGCTCGAGGCCTCCACACCTCCAATTCCTCCAGCTCGAGGCCTCCACACCTCCAATTCCTCCAGC

>chr5(+):1662698-1662917

GGCTGTTCCGGCGAAGGCCCCACGCCATTCTTCTTATTCGTTGGGCTCCTAGCTCGCACCGGCGACCCTGGAAGCTGCCCCGGGCTGAGGGAGCTTCTTCCAGCCGCGCCTCAGCCCAGGCGACTCCCGCCTGAGGTCCACTCGACCTTAGGACCCCCCAGACCTCGGGATCCCCTCAGATCTCAGGACCCCTCAAGCCTCAGGACCCCCTCAGACCTCA

>chr5(+):153935548-153935767

GGGGTGAGCTCCGGCACCCCAGCCTGAGCTTCCCCTCACCCTGGCAGCTCCTGGTCCCTCACCCCACACCCATGGCCACGTCTTACTCCTCTCACACCTATCCTCAGCCCTGCACAGCCAGGCCTTGCCCCTCTCACCCCTGTCCTCAGCCCTGCACAGCCAGGCCTTTCTCCTTTCACACCTGTCCTTAGCCCTGTGCCCCATGCCCAGCGTGTGCCTG

>chr5(-):142002064-142002329

CTCTCAGAACTCACTGGGAAGAGAAGCCCTGTCCCCCAGCCCTGTACACAGGGTGCTGTAGAGGAGGACCAAAAGATGCCACCACCTTGTTCTTACTGGAACTCTGTGCAACTGGGAGCAGAGAGGCAAACAAACAATACAAAGGCAGAAGGAGGCTGAGAAGGAGGAACACATAGGGCCAGGCTGAGGATGGAAGAGTCACCCCCAGGGCCACACCCTGGCTCATAAGACAGGGCTGGAGGAGGGTGAATTTTATATCCCTTCTC

>chr5(-):149416080-149416324

TGACTGGGCTGCCCCAGGAGAAATTGGAGGCCCTCCTGTTGGGGCAACTGGGTCAGGCTGGGCAGCGAGGGGCTGGAACCCAGCCTCCCTCAGCTGGTGCCCAGGCTCGCCCTGCCAGATTGTGCTGAGTTGGCCCACGCCTGGGCGGAAGATGACACGGACGCAGACACGCCCGGGAGCCGGAGGGTAGGGGGGTGCCCCAGATCCTCCTCTGGGCCTACCCCCTCTGCCACGCCATCATTACC

>chr5(+):179768884-179769130

GAGGTTGGCAAAGGAGGCCGGCCTGGCTCCCACCCTGGCCTGGAGTGGGGTGGCCTGGGCACGGGGAACAGGTGGTGGCAGCATCTGAGCTTTGCACCACCTGGAATCTGGGGTGTGTGGGGTGATGCTGCCTGCAGATTGAGCCTGGGTGGCTAGTCCAGGCTCCAGGGAGCTGCCCCCTTGAGTGTCCCTTTCATCCCAGGCCAGCCCTTCCTCTGTCCCTTGTGTCTGGCTCAGCCCTGGCTCT

>chr5(+):14288122-14288341

GATCACCCAGGCCCTATCTGGTCCTCGGCCCTGGCTGCAGCATCTCCTCCTGCCTCCTGCTGCTGCCTCCACAATGAGCTCCACTCTGAGCCTCCAGTGGCCTTGGGTCTTCTCAGAGGTCACTTCTTCTGGCTGGACAGCTCTGTTCCTCCTCTGGCCTCTCCTGGTCTCTTTCTGGATGTGGACCAGGCCCTCCACGCCCTCTCCATAGCCCTGCAAG

>chr5(-):139721520-139721739

CCTTTCCAACACACACAGGCACCCAGCTCCAGACAGGCAGGAACTCCCCAGGAGAGACGGAGAATGGGGGAGGCCATGGGCCTCCTTCAGGAAGATAGGAAATGTAGAACCAGACCAGGGCAGAGATTTCAGCCCCCAGCTCTGTGGCCTGCACCACCCCTAGATCTCCCCTTCAGTACTACCTGGAGCATGGAATGTGAGCTGGAGGAGAATCCATTAA

>chr5(-):1042526-1042816

TGGCCCAGATGAACGCCGTGGGTGTGGGGGCCATGTGGACGGTGGTGGGTGAGGCCGGGGGTCCCCACAGCGAGGGCTTGTGGGTCTTGGTGCTGCTTCCATGTGGAGGGGAACTGAGGTCTAAGGTACAGCCCAGGTCCCAGGCCGGGTCACCCCCGTCCACCCTGCCTGTCTTCCGTGTGCTTGGGGAAGTGGAGGCAGCCCTGCCGAGGCCCCACGTCCTCTGTCACCTTTGCAGACTTTGGCACCCCGTGATGCTACCGTCCTGAGTGCCCCGGGGCCTGGCTGGTG

>chr5(+):107245710-107245929

CCTAACCTCCTAACCTCTGGAGTCTGGGCTGGCTAGAATTCAGCCCCGGGACCAGCAGCACTTCAGCAATTTAGCACCAAAAGGAAGCTTTGGTCCACTCTCCTGTCCTCCAGGGGTATATGTCTCAGAATATCCCTCCAATCTTCACTACCCTTGTCACAACCCAGGCCCAGGGATACCTTCTGATGAAGATGACAAGCAGCCCCTGCCCCAGCCCCAG

>chr5(+):170754203-170754422

CTTACATTGGGGGCCCTGAGCCTCCAGCCCTTCCAAATCTATTCTCAGCAGGAGCTCAGCCACACCTGTGTCCCAGAACTGAGGCCAGGCCCAGCCTTCACTCCACGCCCAGCCAGCCCCAAGGAACCGACTCCCTGAGGCTCTATGCTCCCTGCCTCCAGTGGCCCCGTGTCTGGGAAATAGTGGCCCTGGCCTGATGCCCTGACCTGGGCAATCCATC

>chr5(-):136399129-136399348

AGCCCTGGACCAGCCAGTCAGAACTGAAGGCAGTACTAGGGCTGGTGGCAATGGGGGAGCCTGAGCCCTCCTGCCATGCTGAGCGTTAACCCTTGAGTTGCTGGATTTGGGGGAAGTCCAGGAGATCCCAGGGGACAGGTCGGGGCAGGCTGGGTGGCAGAGAGCCTGTGAAAGGCTTGGAGCAATATCTATTTACCAATAGAGATATTTCAGTTTTATA

>chr5(-):1087848-1088141

ATGGTCCCCACAGCTGGGCTCCCTCCTCATCCCCATGGGCGTAGCCCACCAGGTGGCTGCTGAGTCCTCATGGTCCGTAGGCAAGAGGAAGGGGGAAAGTGTGAGCCCTTGGCCTCCACGGCAGCCGGCTGGGATCCTGGCGGGGGACCCTCGCTGGTAACAGCATCTCCCCAGGACGTGCGGCCCCAGTGCCTGCACCCAAGCCCAGCCCTGGTGGGGAGCAGCCCCTGGAAGGCAGGAACCACTCCAGGCAAATGCAGCTGGTGCTCAGCTACATCCTCACGAGGGCCCTGG

>chr6(-):169875726-169875945

GAGGTCACAGCCGACAGGGAAGGCGGATGGGGCCAACAGGCTCCCCAGGCCCTGACTGGACGTCTCATCAGCTGGACCCCCGGAGCCCCACCTGGCTGCCCAGGATGCTGTGACCAGGAGAAGATGCAGTCACGGCTGCCCAGCCCCACAGATGATGACAGCGATGGTGGGGGTATGCACCCAGCACCTGTCAGCCCCACCAGGCCCCGTGCTGTGCCCT

>chr6(-):82983439-82983657

AGCTGGGCTGCAGCCCCTGCAGGCAGCTGTTGAACACTGGAGGGCCGCCTGGACCACATGGGATGGGCTCCTGAGGACATGGGGCAGTAATTTTGTTCTGTAGTGTGTGGCATGTGTGGAGGCAGGTGAGGCCCAAGCTTGGAAACCCAGGCCTTCCTGTTCACTCTGAGCTGCTTCCTGAGGCAGGTGTTCAAGCAAGGCTGCAGGGGCAGTGGTGGG

>chr6(-):137984152-137984371

CATGGAAGGGGCAAACAGGAGACACTGCTGGCCAGCCAGTGGCCAGGAGAAGCCATGGGCCAGCTTAGAGGAGGGATGTTATGCAGCAGTACCTGGGAGACACCTTGTAGGGACCTCAAGAAATATTTGAAAGATACCTTACAGGGTATTAAAGGTCCACCATCCCCAGGCTATGGAAAAAACCAGGGAAATTTGAGTTGGGGTGGTTCCTGTTATCTGT

>chr6(+):163316236-163316455

AGCTCCTGACAGCCTCCCTCCTGACCTGGCTCCTTCCCTGAAGTCCAGCCTCATCTCTCGCTGTTGCCCCACCTTCAGACCCAGCTTCAGCTGTCCCGAATTCTGCAGTTCCCCAAGGCCACCATTCACCATGGTTTCCATCATGGTCCCCTGCCTTTGCCTGGGATGCCCTTCCTGCCTCAAGGGAGGCACTGTCGTTTCCTCAGGGGAGCCTGCTCCC

>chr6(+):145547276-145547495

AGAAAGTTCTCAGGAACCTCGGAGACCACTGGTATCAGGTGGAAACCAGTCCATTCCCACAGCACAACCATCTGTTCCACTTCCAGTGGCTGCTCTGGGTCCCACTGTGCTGTCCACCCCCATGGGCTGCCGGGCACCTCCTAGAACTTTTACCCCCTCCTGCAGCCCCTGATATCACATCCCTCTGCATCAGTACCTGGCTATGGGTAGGTTCCAGATG

>chr6(-):34140515-34140826

GTGACAAGAGCAGAGGCAGGGGGCTCTGCCAGGGCAGAGAAACAAGATGGTGGCTCTCCCTGGTGGCCTGAGCCTAGGCCTGGCTGAGGGAACAGGGTCAGAGGGCAGCAGTGTGGGCTGCTTCAGGCCACAGGGCCCCAGAGAGGGCAAGATGGGTGGACCAAGGACAAACCCATGCCAGAGACCAGCTGAGGAAACCAGGCAGGGAACCAGGATCCCAAAGGGTTCCCGCCAAAGCGAGGCATCTGTTTTGGGCTGGGACCTGCCGGGGGACACTACCTGGCCTGCCTGCTGTTTGCAGCCTCAGACGGC

>chr6(-):95038414-95038633

GGAGTCTCTGCACAGGGCCTCCCGGGGCCGCATTCCCGGGGTCCGCTCCACATCAGGTAGCCGCCACGCCTGACACTACCAATAGCTAGGCCCGGGGCGCCATCCACTCCCGGAGGCACCCCCTCAGGCATGGCCGCTGGCTGTGTAAGAGGAAGCTCTGGGCCTGCCTGAGCACCAGGGCCACAGGGGAGCTGGCAGCAAAGTTGCCCCTGCCCCAGAT

>chr6(-):89222971-89223190

CCTGGAGTCGGAGGAGGACTCAAGGCGCATAATGAACAAGAGTCTAGAACAGGTGTGGAGCAGGTGCCAGGGATCCGTGTCACATCTGCTCAGGGCAGCCCCGGGAGCCTGCCTGACCACACAGAAGGGGGTGGCTCTTCCAGCACCCCAAGATGCAAGGCCAGGGAGATGACAGGAACCATGAACAACTGCTCCTGCTCCAGGCCTGGCAATGGACAGA

>chr6(-):38131795-38132014

AGTCCTGGGTGTGTGAACTCAGTGTTGCAGGCCCTGCCGTCTGGGATCCAGTGCCCTCTCCTGTGACCCCACAACTGGGCCTGAGTCTCCAGCAACTCCGCTGGTGCATGTGGGGCCCAAGGCTCCATGCGCTGAACATGAGGGACCCCGGCCACCTGGAGCCTTGGCCTCCTCCTGAGCTGTTGTCACGTGGAAACCAGGCAGGGGTCTGCTCCTCTCA

>chr6(+):134282733-134282951

GAAATAGACCGAGTGGTGGGAACAGGTAGGGTTGGGTGAGCAACCCCAGGCGGCCTCAGGTGGCTGCCCAGGGAACCTGGGCCTCACCTGAACAACCCGTGACTCTACATCTCCATCACTGCAATGTGTACTCAGAGCCCAGGCTGAGGGCAAGGGAGCTGGGGCCCCTGTGAAAATGGGGTAGGGGTCTCAAACCCTGCCTGCCTTCCTGTACTACAA

>chr6(-):38157430-38157658

CCACCCAGACAGAGTCAGGCCAGTCTTAAGAATAGACAGTGTCGGGGGCAGCGTTTTGGAAGCAGGAAAGAAGGGGGTAGAGGCCTGTGGAGAACTTGCCACAACCCCCATGTGCCATCACTCACATGGTGGGGTCAGAGGGTGGTGCAGGACCCCCACTGCCCCAGCCCAAGTGCTGACCTCTGCAGCCCTCCTGCATGCCTGCCCTCTTCAAAACTCTCCTCCTCCC

>chr6(+):167801061-167801305

AGGGCTCAGCCCTGGGCCCCTGCTGCTTGGAGCCGGTGCGGTGGCCACCGTGACCTCACGCTTTCCCTGCCAGTCGCTCTCCGCCGCATCCCGGCCTCCCGCCCCGCCCCCGGCCGCTTTCCAGGGACTGAAGCCCGAGTTGGTGCTGCGTGTCCCCGCGGGGATTATTTTCAAACAGCTCCTCCTGCCCTGGCTCCCACAGCGCGGCCCCAGGCAGCTGCACCACTTTCTCCTCTCACCAATTA

>chr6(+):5768274-5768493

ACAGAAAGCTAAACAAGCCCAGCCTCCCTGCCTCACGCCTCGGGGCTGACTGGCTCTGACGTGGTTCGGCTGTGCTGTTCCAGCCTGGGACGGAGCACCCCGCTGCTTGGCCTTTTCCGTGCCTGCATCCCCAGCAGGCTCTAACTGGGATTTGCTGGGGACTCACTCCAGGCCAGGAGAGGGCCTGTGAGCTCTGGCAGTTTGTGCCGTGTGTGGGCAA

>chr6(-):169288736-169288955

GTGACTGGAAAGCCATGGAATGAAGCCGAGCTCCCTTCGGAAGGGAAAGCTGGGAAGAAAAGTGACAGGCAGCTGGGAAAGAGCTTGGGAAAAGCCACGTCCTAAGCGTAGAGAAGGGGCCACCTGAGAACCAGCCTGAGGCTGGGTGGTCTCCGTGGCAAGACCCAGGGGGCCTGGGGGTTCTCCAACAGCCCCTCAGGGAGCCCGTGCCAGCCGTGCC

>chr7(-):46877757-46878022

CCTCACCAGGCAGAGGGACACGGTGGCAAAGGGGACTGCCTGCGTTTCAGTTCCTCTGTTCTCCCGCCACCCTTCTGGGGAGGAAGCGTGCCTGGTTTGGCCACCATGTGGAGGGAGCCTCGTGGCTAATGGGCCACTCCGAGCTGTCAGAGGATCTACTGGCTGAGGACATCTCCGGAATGGCCTTTGGAGGTGCCGGCCATGGGACACCATCATGGTGACTTCTTCCTGGTTGTCTGGCATGCTGCCCCACCTGGTAGTTGTAA

>chr7(-):153942390-153942609

CAGGTTCCAGGAATCCTTTTCCGGGGGGTTTTCACCAAAGCCCTGAGGCCCACCCCTGCTGCCGCCTCCTGGCTGGGAGCTGCTGCCCCCTCTAAGGTGATACCCCAGTCTGCAGGGGCGAGACCTGCTCTCCCAGCCTCTCCTGCCCCGAGCCTGCACCCAGGCCCTCAGGCTGCTATGTGGGGCTGGCCACTGGGGACACAGGCAGGACCCTGGGGGT

>chr7(-):65008173-65008398

AGCTGGGCCGAACGTGGCTTTTGCGAGGTAGGAGCTGGGCCGGCAGGCACAGCTGAGAGGGAGAGTTGGGCCTGGAGAGGCCGGCTGGAAACAGTACTGGGCCTGGAGAGGATGGTGAGAGGCAAGAGCTTGGCCCATGGAGGCTGCCAACAGGCAGGGCAGGAGCTTCGGCTGATGTGGCCACGCTGAGGCAACAGCTGGGACTGGGGGGTTCCACTTTTAGAAG

>chr7(-):61707157-61707480

CTACATATGGCTGTGATCTGAACTGGGGTGAGGCCAGGGGATGACCCTGTCCCCCTAGTCTGGGCTGGAGCTCCTATTTCCCAGGCTCATGCATGGCTGTGGGTGGGCCCTGAGGGACCCTTTGCTGGCTGGGTCACAGATACAAGGTCCTGGTCTGTGAGCAGCCTGTTCACCGGCTCATGCAGTACAGGGGCCCTGTGGAGTGGACTCGTTGTCCTCCGGCCCACCTCTCTCTGGCCTGGGAGGAGTCTGGGCAGGCCCCTGCCCCAGGGCCAATTCCATCCTGTGGCCTCTTTGCTGATGGAGCCGTCTCTTCCCTCAAAG

>chr7(+):149264065-149264284

GGGGCCTGGGGCAAGAAGTGAGAATGGCACCCACTTCAGGGACCTGGCCCACTCCATCAAAGGTGCTGGGTTCCTGTGCCTTAGGAGGAAGCTCTGTGCAGGGCTGCCTGGGGCTGCTTCCCTGGGATCTACCCCATGTTGGGGTGAGCGCTGAGCCTGACGCTCCTGATAGCCAGGCCTGGGGCCCATGATCCACTCCACCCTGGGCCTCCTCTGAGCA

>chr7(+):99993968-99994187

GGTTTCGTAATGTGCTCGCCCTGCCCCAGGCATCAAGGGAGGTGCTCACTCTGTGCACTCTGAATCCCACCAGGGGCCCACCTGCCACCCTGTTCTCACCTGTGACCACTCACGGCAGCCACAAGAGGCACAGGTGTTAGGTCTGCCCCAGCCACAACACCTCAGCACGTGGTGTGTGGCTTCAACCAGGTCACCGTGGGCACACTGGGAGGGGAGGGCT

>chr7(-):127890130-127890441

GGGCCGCCCAGGGCAGAGGTGGGGGAGTCTGAAACCATCTGTAGGGCCATCCTGAATGGTGCCGTGGGTTGGAAAGGCCCAGCCAGGCTCCCAGCACACGCTTGGGGTGAGACCTTGGCTGGCAGCACAGGGCCTGGTCTTTTCTGCTGCAGGTTGTGAAGGGGGCAGCTCCAGGCAGGTGGGCTGCCCTTGAGGTCAGCCTGGGTGTTTCCAGGGGGGCTACCGTCTCTGGAGCTGTAGCCTGCTGTGGAGTGGGTCATCTCCGAACAGCTCCAAGAGGAGGATACTTAGTGGGGCTCGGAGTTGGAGGGC

>chr7(-):43658635-43658897

GGAGGCAGAGCCTGGGGGCCTGGGAGGGCGAAAGGCTTGAGCATGTGGGTGTGCACATGCGTGGCTCCATGGTGCATGCACCACATACACACGTGTGTGTGCAGGCATGCGGGCACAAGTGTGCATGGACTACACGTGTGCGTGCAGGTGTGAGCTGTGAGATGGGCACCCAGAGAGTGTGAGCTTGGCATGTGTGGGCATGTGAGAAACCTATCACATCCCCCTAGAGGGTCCAGAACCCACAGCCTACAGAAGGGCCACAG

>chr7(-):57079832-57080143

GGTGAGATGGGTGGCTCTGGCCCCAGGACTTCTCCCCGGGACCTCTGCTCAGGACATGGCAGGGACAGAGGTGAGGTTACCATAAACTGCCTTGGAAATGACTACAAACCAGCTCACACCATTCCAGCACCTAACACTCTGACAGCCCAGGGTGGGCCCACAGAAGGGGAAGGGCTAGGGAGTCCAGGACCACAAGAGTCCCCCTTCCCTGAGCCCTTGAGCACAGCCACCCAGGTGTAAGGGACAATATGGGGGTTCTGGGGATTCCCAAGCCTGGGCTCTGGAGGTGAGTTCTAGCAGGGCCCCCAGATA

>chr7(+):127885902-127886149

AGCCAGCAGCCCAGGAAGGAACCACTCTTAGTGACCTGTGGAAGGTTAGGAGACCCTCCCTCGGGGCCCTAGAGCCCACCCTGAACAGGGCCTTTCAGGGGAGAGGGTGAGAAGGTTCACCACCCAGAGGAGCCAGGCCAAAGCCCTGAAACCAAACATCCCCCAGTTCCCTCACTCCTCGATTCAGACTCCAGGGCCACAGCCTGGAGCAGAGCTGGACCCCAGCTCCTCTTGGCAGCACCCCTGAG

>chr7(+):44424974-44425315

CCTGAGGTGGGCTTGGAGTAGGACCTGTGGTAAAGCCTGCGTAGGACCTAGGGTACAGCCTGGGACAGAGCTTCAGAGGAGCCCGGGGTCCTGGTGGACCCTGTGAGGCTATGACTGGCTCAGTGAGCCCTGGCTGCTGCTGTACTCTGAGTGAGGAGCGGCAGGGTGACCAGGCTTTTGGAATCTGTGCTAGGAAGTGAAGGTGGACGGGAGAAGCAGCAGGAGATCAGCTGCAGCCACCACACTGGTCCTAGGCCCAGGTGACAGGACATAGGTGTCTGGTGCCACCTTCACTTCACCCCTCCTGACCTCTGACCCTGCACCGTGGGCATGCACATTAAC

>chr7(-):65006938-65007225

GGCAGCCACAGCCGGGAGGGAGAGCTGGGCCTGGAGAGGCCGGCTGGAAACAGTTCTGGACCTGGAGAGGATGGCGAGAGGCAAGAGCTGGGCCTGTGGAGGCTGCCAACAGGCAAGGCAGGAGCTTGGCCTGAGGTGGCCATGCTGAAGCAAGAGCTGGGTCTGGGGGGGTCCACTGTGAGGAGGCAGAGGCCATGCCTCTAGGCGCCTACGACAGGCAGGAGCTGGGCCTGGCGAGGCCGACTTCAGGACGCTTTGGGCCCACACGGGCCATCGGCGGGAGGCAGG

>chr7(-):71472583-71472802

CCCACGAGAGCCCTGCAGTCCTTCCCACATCCTCGGCCGAGGAGAAATCTCCAGTATTCTGGGCTCAGGCTCCCCTCTCCCTCCCGGGTGGCTGAGCCAGCCGGGGGTGCCTTGGCTACAGCCCCCTCCCCTGTGTCTCAACCCCAGCCACCCAGTAGGAGAGGTCAGGTCTTTCTGATGCCCGACATCGCCTCTACCCCGCTCCAAAGCCTTCACTGGC

>chr7(-):51098142-51098361

CAACATCTACCCTTCCCCCACTGACTGGAGGCCTTGCGTTCAAATCCACCTTCTCTCTCCCTGCTGGCGAGACTCCTGGTTGAGTGGCATCTGCTCTCTTCAAGGCCAGAACGCACAGGGACGCCCTTCTCTGCAGGGGCCCATTCCTGCCACCTACCTTGACCTGGGTGGGAGGAGAAGGAAGCCCACCTGGCCCCAGAAGCTGGTTTACGCTCTGCTC

>chr7(-):1026075-1026324

CTCAGCACGGCCCTCAGAGGACTCCCCCAGCCCAGGGAAGGCATCCTCCAGGCCTCCATCTCCAGCCCACCCCAGCTCAGGCTGCAAAGGCCTTGGCCATAGCTGGGGATGGGGAGCACAGCAGTTCTGCCCCCGGGAGGACCTGAGGCCGGGGGACTGAGCATGGGCAGTCTCGGGGGAGCAGAGCATGGGGGGACCACTCCATCCAGGAGGCCCTGGAGGCTGGGTGCACCGGATCTTTATTCTGTCC

>chr7(-):133366460-133366679

TGCTCAAGGCCCAGATGTGTTCACCCTCCCCCTGCACCATGAGGTTGGCTGGAGAGGGGAGCCATTGAGGAAACCCAGGCAGGGCCTGAGTGGGGAGGCAGGGAGATGCTCCAGGGAGCAAAGCCCACCCTGCAGGGGAGGCTGGGAGTGCATTAGAGCCCTGGCACCTGGAAGGGAGACTCTTCATGGAGACCTGGAGGGTGCTGGCAAGAATGTGGCT

>chr7(-):72046554-72046773

AGCGGGCCCTCTGCCAGCCCGGACGTTCCCAGGTCTGGGTTGGGGAGGCAGCCCTCACCCTGTCTGCTGCCCAGGCCCCTCTCCAGGGGGCTTGGTCATGCCAGGGAAGCCCCCAAGAGCAAACAACTGGCCCAGGCCATCTGGACCATCCAGACCAAGGGCGGCCGGGGATCACTGACGGCATTTGCCCAGACTTCCCAGGCATCCATGCTGTCCTCAG

>chr7(+):1041676-1041941

GCACACAGTAGGCATTTCCTCAGCACACAGTAGGTATGCAGTGCCTGCAGGAATGGAGCCCTGGAGGTCTACTGGACGGGGCAGCACTGCAGCTGGGAGTGGAGGAACCTGAAGGAGCAGACTGGGGGGCCCCACGAGGGGCCACGGGACACCCAGGGAGGGTGGGGAGCTGCTTAGAAGAGGTTGGAGCCCAGGGAGGGCCTGGGTGGGACTGTCACGGGGCGAGTGGAGAGATGGGCTGTGGGCCGAGACCCAAGGCTATAAAC

>chr7(+):45549856-45550075

CGAGGCAGGGGCTGCCTTCCTGGTGCCCAGTGTTTATGTCCGTCCACAGAGGGTGACCTCTAGGGAGGCTGCTGAGAGCTGAGGCTCTCAGAGGGCTGTGTGGCGCAGGCACTGCCTGTCTGGACTCCCAGAAACTCAGTTTGTCACAGCGACTGAGGAAGATGCAATAGCCTCTGATCCCACAGAGGGGCCTTGTGGGGATCCAGGCTCAGCTGGGGGC

>chr7(-):127885902-127886149

CTCAGGGGTGCTGCCAAGAGGAGCTGGGGTCCAGCTCTGCTCCAGGCTGTGGCCCTGGAGTCTGAATCGAGGAGTGAGGGAACTGGGGGATGTTTGGTTTCAGGGCTTTGGCCTGGCTCCTCTGGGTGGTGAACCTTCTCACCCTCTCCCCTGAAAGGCCCTGTTCAGGGTGGGCTCTAGGGCCCCGAGGGAGGGTCTCCTAACCTTCCACAGGTCACTAAGAGTGGTTCCTTCCTGGGCTGCTGGCT

>chr7(-):127886710-127886929

ATTAAATGGAGCAGTGACGTGGGCTTAGTAAATAGCAAAACCCAGCCCACATTAGCCATCTCTTCCCCAGCGCCCAGGGGCAGGGCTCTCTGGCCTGGCTTGTGCTGGTTCCCAGAGTCCCTGCTGTCAGGTGCTGGGAGTGGAGGTGCCCTCGGGTTCCCTTGGGAGTGGTGACCACAGCTCAGGGCCTGGGTTGGATCCTGGTGGCTGCCCTAGGGAT

>chr7(-):72489144-72489360

CAGCCCCACTCACCTTCCTGGGCTGGCCTCTTGGAAGCCCCGGGGTTCTACAGGCTCAGGTTCCCAGCTGCCCTGGCCCTCACATGCTTGGTGTTACCCGGCTGGGCTCCCTAACTGGGCCACCACTGGGAAAGGTGCTGGGTTAGATGGAGTCACCCCTGTGTGTGACCCATTTCTGTGGACCCCACCTGCCCTGTGTACATCTGCATATATGATG

>chr7(+):30414648-30414867

CTCCCCACTGCCGCCTTTTCCAGCCCCCAGCTCCTTGGCTGTCACCGCTCTGCCTGTGGAAGCCCGTCCCCAGCCCACACGGGCCTCTACAGGAGCCCTCAAGGACGCTCCGAGTGGCTGTGCTCCTCCGGTCTCTCCTGGTGCTTCTCCTGGCTGTGCCTCCTGCCAGAGGCAACCAGCCACACCCAGGCCACGTTGCCACCCTCTGCACAGGTGTCGA

>chr7(+):44978989-44979208

CCCTCTCTGGCTGGCTGGCAGACACTGTGCACATTAAGTGTGCAAGTGCAGAGAGACTGAGAGGACATCACTTCCAAAGAGGGGAAGCTGGCCCCAGTGGAGGGTGGGGAATAGCAGGGAGGGTCAGGATCCCCATAGCCCAGGGCTGTAGGGGTTATGGAGGTACTGGTCAGTGTTAGGCCTTTGTGCTGGAAAAATCCATTCTTGCATACTCAGGGCT

>chr7(-):72671831-72672050

TGTGCAAGCATGGCCACATGGTTTTGTTTGTTACTGGGCACACGCCTGGCTCCTGTGTGCCCCAGCCCAGCTCAGCTGCATGTAAGATGGGATCCAGCCACCATCTCAGAGCCCCAGAGCAGGGCAGGTAACTGGGTCTGCTCATGAGAGCCAGGGCACACCTCTGGGGAGCCTGGGGCCAGCGCCACATGCCACAGCTGTCACTCTGCTCCTCTGCCAG

>chr7(+):99903085-99903396

AGAAGGACTGGAGTGTGTGGAGCAGGACGAGGCCCTAGGATACCAAAGTAGGGCAAGGCTGGGAGGGCTATTGCTCCCCTCTGTCCTCAAAGGACACCCTTAAAGACACCTGCACCTCCCAGGACAGAGCATTGGTGCCCTCAGAGATGCCCTCTCTCCATCAGGACATTTCCCAGTGCCCGGCAGTGGTCTAGCCACACAGTCACCCTGCATCATCCTGAAGAGTGATCTGGAGGGCTAGACCTGGCTGAGATGGGCTGCAGACGCTCCCGGGCCTTTGGCTGATGGCAAGGGGGTGTGACTGCTGAGCTC

>chr7(+):156538951-156539170

GGGCTTTGTCTCCAGAGGGGGCAGGCCCTGGGTGACACAGCCCCTCAGCCCCACTGCCCTGTCCCCGAGGCTGGCCAGCCCTTCCTTATCTGCACAGCCTGAAGCCTTGTGTGGCTGACTCGGCCCTGAGCTTGTTCTCACCACCAAAGGAAGAGCCAAGCGCCCCCACCTGCTCACCTCTGCCCTGACTGTCCTCACCCTGGATCCCTTCCCTAATTAA

>chr7(-):61435533-61435794

GCTAGCGGGTGAGGTAGGACTCAGGGGCCACGGACAGGACAGGGCACCCAGGCTGGCCTCCCCCAGGACCCTCTAAGGCCCCTGTAGGCAGGAGGGCTCAGTGGAGTTCCAGGACCCACTTCAAGGATGGTGCCTGTAGCCCCTCCCTGGACCTCCAAGAAGGGCCAACTCCCTCTGCAACCTGCACCCACACAACTCCCTCTAAAAACTGCACTCACCCAACTCCCTCTGCAACCTGCACCCACTGATCTCTTAGAGGTGA

>chr7(-):38996537-38996756

CAAAGGAAGTCGGCTTGAGGTCGCCATTGTCCTGCTTCATGCCTCTTCCCTCCCTTCCCAAGCAGCTCTTCACCTCTGTCTTGGACACAGGATGGGGAAAGGCAGGCAGCCAGCGGGGCCCCTGCTCTGAGGCAAGGCAGGGGACTTCACCAGCACCCTGCCGCTGGGTCGCAGCCTCCCCTGGTGGCTCTTGGCTCTGGTGTGCCCTCTTGCCTTTCAT

>chr7(-):65008455-65008696

GAGTCAGGCCCGCGGAGGCCACCGAAAGGCAGGAGTGTGGCCTGGTGCAGGCCGTGGTGAGGCACGAGATGAGCCTAAAGATGCCATCGGGAGGCAGGAGCCGGGATTGTCGGGGCTGCCGCGAGGCAGGCGGAAACGTGGCCCAGGGAGGCCAACGTGAGGCAAGAGCTGGGCCTGGAGAGGCCGCTGTCAGGCAGGAGCTGGGCCTCTCCATGCCACCGGGGAGCCAAAGGCAGGGCCTG

>chr7(-):44910161-44910426

CTCTGAGCAGGCAAAGGAATGCAGCACCAGGGGGAGTGGATGGCCATGGAGAGCCTTGGGCCCTGTCCTCCCTGCCACACCCTCTCAGGCCCCAAGGGCTTTGGGGCAGGCAGGACCCCAAGGCAGGCTGAGGCCAGAAGCCCACACCAGCTCTGCGCACTCTCTGGGCTGAGGGGTCAACCCAGAGCCAGGGCCAAGCTGGCAGGAGCAGCAGGCTATGGGCCAGGCCTGGGGGCGATGGAGAGCCGGCTCCTCACCCGACTCCC

>chr7(-):61844494-61844808

AGAGGCCACCTGGGGGCAGCAGCTGGGCCTAGAGATGCCAACGAAAGGCAGGAGCTGGGCCTTGAGAGGCTGACTTGTGGACATTTTGGCCCTGGATTTTGTGGCCTGGACTTACGGACATTTGGGCCTCCTGCAAAGAGGCAGGAGCTGGGACTAAAGAGGCCATTGTAAGGCAGGAGCTGGGCCTGTACAGGCTGCCAGGAGTCAGAAGATGGGCCTCAAGAGGCCACTGTAAAACAGGAGCTGGGTCTTGGAAGGTGGCCGTGAGGCAAAAGCTGGCCCTTGGGAGGCCAATGAGTGGCAGGAGCTGGGCCT

>chr7(-):54809646-54810030

TGCATGGAGACAGCCTGGTCCTGGGGACTTCCTCCCCTTCCATCTTGGGTAGCATCCCTGTAGCCCTTCCACTCCTCACCCGGTTAGCAAATCCCTCAGGCCAACACCTCTCTGGCACCGGGGCACCTTGATGGCCCTTTCGTCCATTCCTCAGTGCACTGCTGCCCTGGGGAACGTGGCCTCCTTTCAGCTCCTCAACCACCCCTGAGGTCCCAGGGCACCCCCTCCCTGGGGCTCCTCCAAATGCTGCCTTCCCATCTCTCCTGAAGGGCTGGTGGACATTCTACCTGCATTATAGACTCTGGCTGGCTAGAGGGGACCCTGGGAGCAGTGCCCCATCAGTATTTCAGTGAGTAGCTGACACCCACCACGTGGCCAGGACCCG

>chr7(-):155506835-155507072

GGATGAAAGCCTGGGGTGCGGGTGAGGCTGCAGCCTCTGGGAAGCCAACTTGGCTGGAGGTGAGCAGGAGGGCGCTGCTGCCCTGGCTGTGGGATGGGCACCTCCGCAGTGGGTCTCACTGAAGGCCCCGCCAGCCCAGCAGAAAGCCCCGAGGTCAGGTGACCCCTCAGAGCTGCCTGGGTGGGGCCTGGACATGGTGTGCCCACAGTCACGGGACCAGAGGGAGTGGCCCCAACGG

>chr7(-):127911094-127911322

TGCACGACCTGCACCGCACTGTCCTATGGTCCTGGAAACGTGTCAAGGAGCTCGGGTGGGCTGAGGGACTAAGCCTGTGGGTTCCTCAAGGGGTGGGACAAGTGCCCAGAGGAGACACAGCCTCTGCTAAGGGTGTCCAGGGGCCCAGGCCTGGTGGGGAGTGACCAGGGAAATACCACCACCCCACCACCCATAGGCAGCTTGGAACAGCACAGAGTGCCCGGCTGGA

>chr7(-):56653019-56653269

TCGGGGCAGGGCCTGGAGGCACCCGGAAGCCCGGCAGTGGGAGAGAGGAGCCCCAGCCCCCTGCAGGCTCCTTCCCCTGGGCATCCCGGAGCTGGGGGCCCAAAGGAGGTTAAAGGTCAATGGGCTTGGAGCGGGACTAGTGGATGGGGACCCGGGGAAACCGGGAAGACCAAGGCAGAGGAGGTGGGAAGAGGAGGAAGTGCTCCTGCAGAGCCCCCTCCTGAGGTGAAGGGTGAGCCTGGGCAGTGTCC

>chr7(-):56439439-56439660

CCTGGTGGCCGCCGTGACGCATGAGCTTGGCCTCCGGAGGGCCTCCGCGAGGCACCAGCTGGGTGTGTGGAGGCAGCCCCGAGGTGGGGAGCCTGGCCCGAGGACGCCATGGCGAGGCAAGAGGTGGGCCTGGAGGGCCCACTGTTGAGGTAGAGGCTGGGCGTCTAGAGGCCGCCAACAGGCAGGGGCTGGACCTGGAGAGGCCAACAGAGGCATGAGCTG

>chr7(-):99993968-99994187

AGCCCTCCCCTCCCAGTGTGCCCACGGTGACCTGGTTGAAGCCACACACCACGTGCTGAGGTGTTGTGGCTGGGGCAGACCTAACACCTGTGCCTCTTGTGGCTGCCGTGAGTGGTCACAGGTGAGAACAGGGTGGCAGGTGGGCCCCTGGTGGGATTCAGAGTGCACAGAGTGAGCACCTCCCTTGATGCCTGGGGCAGGGCGAGCACATTACGAAACC

>chr7(+):96684426-96684645

GATGGGGATGCACAGGCCCTGGCTGGCAATGGGTGGAGCTCTGATGGGGTGGGTGGGGCTCTGAATAAGAATGGGTGGGGCTCTGGCTGGGAGTGGGTGCGGATCTGGATGGGGATGCACAAGGCCCTGAATGGGGATGGGTGGAGCTCTGGGTGGGGTGAGCGGGGCTCTGAATGAGAATGGGTGGGGCTCTGAATGGGGTGGGCAGGGCTCTGGATGA

>chr7(-):49945090-49945309

CCACCCTGCTTCACCCTGGGATGGCGTAACTGTGGCCAAGTCACCGTGTGAGCCGAGCCTGTGCCTTCATCCATGATCCAGGGCCTGGTACTGCAGGGAACGAAGGGAGCGGCAGTGCTGGGTACATTAGCAACCCGAGGGCATCAGGTCAGGAACACGGCCAGGCCAGGGAAGAGCCAGGGACAGGCTGGCTGTGGTCCCTCCCTAGCTCTCGGTGTTC

>chr7(-):156677435-156677654

TTCCCCCAAGAAGAGCCTGCTCCTTGCACATCTGTGAGCTTCTCTGCCTGTTGTTCCCTGTCTCTCCTGCTCGGTCTCCATCAGCCTAAGGGCTGATGTTCTGGGCAGAAGGAATGGGGTGTGTTGTAACCCTAGGCTTCTGGAAGAAGGAGGTGCCAGACAATGGGTTCACTCATGAAGCTAGATTCAGGAAGCAGGATGTAGGGTGTCAGGCTCAGAA

>chr8(-):58416875-58417140

TCCGGGACAGCAGGAAGAGCGCGCCCAGTTGGCAAAATCTTTAAGGAATCCTGGGAGTGAGGTGGCCAGGAGTCGTCATTCTCCAGGACTGGCATCCTGCCTTGCCGGGCTTCCTCCTGCTTCTTAGTCCCAGAATGCTGCACCCCGAAGGCCTCCCTGCTGGTTCTTGAACAAACTCTGGGGGCCTCTCAGCCCACAGATGAGGCCAGAGGGAGCAGGCCTCAAGAAAGAAACCCAGGCTCAGGCCATTTCCCCCAAACCCACAT

>chr8(-):143244474-143244693

GTAAACACTAACCTGTGCTGCCCCTGAGCCAGGCCTGAGCTGTGTGCTGCGGCCCTGGAGAGGCAGGAGGTCGCCTGTCCCCCAGGGAAGTTCAGAGGCTCCTGAGCAGGCTGGGGGCACCTGCAGAGGCTGCCTGGAGAGGGCGGTGCTTAGCAGAGCAGTGAGGAGGAGCCAGGCCAGGTGGGGTGCAGGGCTAGGGACTGAAGGCATCCTCTCTCCG

>chr8(-):43673158-43673374

AGGGTGATTTTTTAAATCAAAACCCAGTGAATCTCATTACTCCTAAGAAACGAAAGATTCCTTCAAAGCCTTTTCAGGCACATGGTTTCAACAAAGCCTGGCTTTGACATTCCTTGTCCTGAGGAGCACTTTCCAGGCATAGTTACAGCTTCCCCACTGTATTTACAAGCCAGAATTGTGCAACTCTTCTGGATCATTAATAAAGTAGCAAGATCCT

>chr8(-):141821046-141821357

ATGACGTCTGTGGCCTGGCAGGGGTGATGATGGTGGCCATTATGGAGGGAGGGGCCATGGGGCTGCATCCATAGCACTGATGGAGACAGGTAACAGGTGTGCACGGGAGGGAAGCATGGCCACATCAGGGGGCACGCAGCAAGACGGCACCCTCCGCTCCCACCAGCCCTGCCCTTCTGGATGTTGCTAGGTCCAGGTGTGACCCGTGTCCAGCCGGGGGCCTGACTGCTGACTGGCTGCTGGAGGCTGACTGCCTCCCTGTGTGCCCTCAGGAGACAGGCCAAGGCTGGAGTGCCGGGGGACGTGTGGCAG

>chr8(+):143164071-143164290

CATGGCCAGCCTTGCCCACTGCCTGCCCGGACGCACCGTCCTCAGGGACATCCATGCCTCACCTCCCCGGCAAGCACCCCTCCAGTCGCCACCTCCTGCCCTCTGTCAGGCCCAGCCCTCAGAGCGCCGCCCTATGTTCCCACACAGACAAGGAGAGCTGGTCCAGGGAGGGCGCCTCTCCCTGCGTGCTGGGCCTGGCTGTGTGGGAGGCAGTGGGCAC

>chr8(-):143164071-143164290

GTGCCCACTGCCTCCCACACAGCCAGGCCCAGCACGCAGGGAGAGGCGCCCTCCCTGGACCAGCTCTCCTTGTCTGTGTGGGAACATAGGGCGGCGCTCTGAGGGCTGGGCCTGACAGAGGGCAGGAGGTGGCGACTGGAGGGGTGCTTGCCGGGGAGGTGAGGCATGGATGTCCCTGAGGACGGTGCGTCCGGGCAGGCAGTGGGCAAGGCTGGCCATG

>chr8(-):41081800-41082019

CAGAAGGATGAGCCTGGGTGGCGTGGCTGCAGGAAGCACCACTGTCTCCTGGGAGCAGCTCCAGGGCAGCGGGGCAGAGGCTGGTGTGTGCAGGGGCCAATGCAGAATGTGCTCAGGTGGCTTCTGGGAACTCTGAGCCTCTTCCTGGACCACCCTGGCTGAGGGAGCACATCCAGGCTGGGGCTTCCCGGGGGTAGTAGCTGTTTGGTTGGCCCAGAGC

>chr8(-):143374130-143374349

CACCAAACTCAGACACAGCCCCTTTGCAGAAGCAGAGAACATCGGACAGAAGGTCCAGGGAGCAGAGGGGGTGCTTGTGGGCCTGGCTGGCCTCAACAGTTGCTCCCTGGGGGCTGGGTGTGGGCAGCTGGGTGGATGGGGCTGCTGGCCCCTGCCCTAGAGGGTCCCTGTGTCTATGAGGCCAGCGTGAGGTTCCAGGTCTTGCCTTCTCTGTCCCAGC

>chr8(-):103869563-103869782

AGTCCCCAGTGCGGGAGAGGCAGGCTCAGTGTGGCAACATCCGCAAGTGCGCCTGGCCTGCAGACCACGCAGCTGGCTGTCTTCCCTGGGCTTGTGGGGGCCACCCTGACACCCAGGGGTACAGTACACCACCCCACACCTCAGCTGGCTCTGTCAACTCCCCAGCCTGCCCTGACCTTCCCTTCAGGCTGGGCTGTGCAGTCCTGCCTCTCATGGAATT

>chr8(+):143562258-143562487

GGGGGCCCACAGCGCAGCCACCACAAGGCACCCAGCACACAGTGCCAAGGNCATGGAGCCTGGCCCCTGCAGGCTCCAGACACAGGCCCTCGGTGTGGCACTGCTCCACCCAGCCCTGTCTCCAGACCTCTTCGGCAAACCTCAGCCTGGGCCCCGGGTTCCCTGCTCAGCACCAACAGCCATCAGAAAAGCCCAGGCCTCACCCTGCCTCCCAAGACCCTCACAGGCTG

>chr8(-):142790241-142790460

ATGGGGCAGGGCAGGTTGCTCTTCACAGCCCTGAAGGGCTGGCCTGAGGCCGGAGGTGCAGGTTGCTTCCGTGGAGCCAGAAGAGAGAGGCAGGGCCTGTTGGGGACCCTGGCTTGGTCTTGTGGGGAAGCTCGCAAGTGGAGGAAGGGGGAAAGCGTGTGCCTGGCACCTCTCACGGTGGCCTCATCCAGCCCCCAGACCCTCTGCACAGGGCGAAGCC

>chr8(+):145344172-145344391

GTCTCTGCCTCTGTGGGGAGTCTGTGTCTGCTCAGAGGAGCCTGGGAGTGGCTCCCCAAGGCCTCATCTCCAGCCCTGATTTTTGGGTCAGTTCTCCCAGGCTCACTTCTCTGACCACTTGCCCACTGCCCCCAGCCATGGGGACGTCCCGCTGCCCTTTCCTCAACCGTGCCCACCCCTTGCCCAGCAGAGGGTCTCCAAGCAGCTGGATTCTCAGGTC

>chr8(+):94395494-94395736

GGACATGGACAGGAGGGAGCCCAGAGGGAACCGAATGGCTCTTGTCCCTGCACAGAAGGGCCCTTCTGATGCTGGCCTGGCTCTTTTTTACCCTCCTGACAGTGGCTCTACAATGTAAAGGAAGGACACCCACCCACACTCTGTTAGCATCAGGAATTTATACAATCATTCCAAATGTTAAAAAAAAAAAAAAAAAAAAAAAGGAAACAATCCCAAAAATGTTCCTACCACTCTACATTCTTA

>chr8(-):97504158-97504377

AGTACAGAGTGAAGAGAGGCAGGGACTGGGGCTGTACATTTTGGACCCTACAGACTGGTCCTATCCCTGGCCTTGCTGACATCTGTAGGTGATCAAGGTGAAAGCAGAAGACCCTTCTGTCTCCTCATTCTCTCAGCTGCTGAGCAGCCAAATATGGGGAGCTGCGTGAATGTTTATTTCGCAGGAAGTTCTGCTGCTCCATCATGATGTCTCCTGCTTA

>chr8(-):142313857-142314076

TCAGCATCCTAAGCCCAGACCCATCTCCTTCCCAAAACCTGCTCCCCGCAGAAAGAGGCCACAGGTCGGCAACCACAGCTGCCACCCTGGCTGACAGAGCCAGACACCTGGGTCAGCCCAGCCTCCTTGTGCCACTGCTGGACACTGGCCACCGGCCCAGTCTTCCCTTTCCCCACCGCAGATGAGCCCCTGCCTCTCAGGCCTGACAACTCTACATCCC

>chr8(+):21497538-21497757

GGCAGGGAGACCACTGAAGGCAGCTATGATAGAGAGTCAAGTGCAGGCATTTAGGAAAGGTTAAATTTCTGGGAGCAAACAGTACCTGTGTTGGCTGCCAGAAGACAAAGAGGGGCTTGAGGGGTGAGAGAAGGGTCCCTCTCCCGGAGAAAGGTCCCAGCACAAGTCCCGCTTTAAAGGGGGTTGGCCAGTAAGCTCTAGGGGTGCCCCACAGAGATGC

>chr8(-):94890629-94890894

TCTGGCCCTTCAGCTTCCTCCTTGCTTTCCCCAGCGCTCTCTGGTGCCAGGAGAGCCTGAGAAGCAGCAGGTGAAAGCCTAGTCTCCCACGCAGCCTCTTCCATTTGCCCCCACCGCCCCTCAATGTTGCTACTTACTAAATTGCTGGCTGGAGAACTCCTCACCTGTGGCAGGCGTTCTGTGGGAAAAACAGGTCAGTTGCTGCCCTGGGGGAGCCCAGCCACAGCAGAAAGGGAAAATGCTGACGGGACAACTTCTACTTTAAC

>chr8(-):70885526-70885770

GGGGCTTTGGGCTAGAGAGGGGGCAAGCGAGCCCGAGCGCGGACCACGCGCAGCGGCAGAGAGGGAACTGAGCGCGGCTTTACTCCCTCCCTCCGCTCACGCCCCAGGCATCCTGGAGCTCCCAGACCCGCTCCTGGCAGAGCTCCGCCCGGCCAGCTACTGCCCTAACCCTGGGGGCTTGGCTCCCTCTCCATCTATCTTTCGGGAGTGCCCAACCACAGCTAGAGACCAGCCGATGGAGGAAA

>chr8(-):142487329-142487548

TGAGATCTGGGATCTGAAGCTGTAGGGCTGGGGGCACCTCGCTCCAGTGTCCGGGGCCTCCAGGGCAGAGTTGGCTCTTGGTGGGCTCCTAGTGCTGAGGACTTGGAGGGTGGTGAGGCCTTGCCTTACCTGGAGTGCTCCCTGGGCTGGGCTGGAAGACCCCGGGCCATGGGCTTTCTCTAGCCCTCACTCCAGGCCTCCCTGAGCCCCCCTCTGCAGA

>chr8(-):103981384-103981603

GCCAGCTGGGAGACACCCTTCCTTCCTGTCAGTGTCTTAGAAATGGGGGTGAGCAATCAGCAGCAACAGGTGGCAAGGAGAGTCCAGTAGGTAGTGTGGGAGCCAAAGGATGAACAGGGCTGAAGTCTCATTTCCAGAACAAGAAGGTCAAGGAACAGATCCTGGATTCTCCTGCCACCATGAATGCTGACTGAGAAGGCCATTCCTGCTTCTTCCTCTC

>chr8(+):92017443-92017826

AACTGCATTTAAAAAAAGATCTATGACCAGATATTCTCCTGTGTGCTCCTCAAAGGAACACTATTAAGGTTCATTGAAATGTTTTCAATCATTGCCTTCCCATTGATCCTTCTAACATGCTGTTGACATCACACCTAATATTCAGAGGGAATGGGCAAGGTATGAGGGAAGGAAATAAAACATAAAATAAATAAAATAGAATGACACAAATCTGAGTTTTGTGAACCCCTGAAGGGATGGTCTAAAGGACGTTATCTGGAACTGGAGAAAAGCAGAGTTGAGATACAATTCTATAGATTAAATCCTGGTAAGGATAAACATTGCCATTAGAAGAAAAGCTTCAAAATAGACCTGTGGCAGATGTCACATAAGTAGAATTTCTGC

>chr8(-):103661985-103662204

ACTGGGTCAAACAGCTCCAGAAAGCTCTTTCACCCATGTCCCTTTCAAGGGCCCCCAAATCCAGCCTCCTCCTGCCAGTGTCCACCTGGATCCTGGGAAACAGGCTTGCCCCTTGCCCTCTCACTCTCATCAGGCTCTGCTGCCTGACGGAGTCACCCCGAGGCAGAACCTCTCCCTTTTAGAGTTTCCCAGGGAGACAGGTACCAGCCCTGTTCCTCCA

>chr8(-):143695856-143696075

CCAGGGAGGAAGAGCTCACCAGCAGGATCAGGGCCATGGAGGTCATGGAGCAGAGCCAGGTGCTGGAGACCATCCTAAACCAGCTGGTGGAACACAGCCAGGTAGGATCCCCAGCCACGCTCTGCACCCTCTGTGGCCCTGACATGTACAGAGCCATGGGGCTCCGGGGGGCCTCTGCAGGACTCACTGGGCAGCTGGTGGCCTTGTCAAGCCCGGAGCC

>chr8(-):143562258-143562487

CAGCCTGTGAGGGTCTTGGGAGGCAGGGTGAGGCCTGGGCTTTTCTGATGGCTGTTGGTGCTGAGCAGGGAACCCGGGGCCCAGGCTGAGGTTTGCCGAAGAGGTCTGGAGACAGGGCTGGGTGGAGCAGTGCCACACCGAGGGCCTGTGTCTGGAGCCTGCAGGGGCCAGGCTCCATGNCCTTGGCACTGTGTGCTGGGTGCCTTGTGGTGGCTGCGCTGTGGGCCCCC

>chr9(+):65996737-65996965

GGAGCCTGGGTGGCTTCACCTGACCTTGTCTACTGGGCCAACTTACCCGCCAGATCCCCCAAAGCCAGGGTCTGTGGCGCCCCTTTCTCCCCTCTTGCTCCAGACAGCGAGGGGGCTTCTCCACAGTTGAGGGCAGGGAGCGCGGTCTTTGCCGAGCCCGCACACTGCTCCCAGGACAGCCGGCACAGGCAGACGCAGCCCTGGCACCGGGAGCAGGTGTGGGCTCCAG

>chr9(+):33390702-33390921

CTTAGGTCCTCACCAAGGCCCCTTCCCAAGGTCCCTTCCCAAGGCCCAACCTGCCCACAGTCTCACCGAGGCCAGCAGGGCCCAGGTCTGGGCCCAGGTCCAGTCCCAGGCAAGGGTATTGTGTGTGCTTCCTGATAGGTCAGGCCGCCCTGAACCCTCCAGGCACACTGGCCCTAAATGTGTCTGCTTCCTGAAGGGTCAGGCTGCCCTGAACCCTCCC

>chr10(+):111456422-111456641

CAGTTCCAAGGAAGCTGTGGTGGCCTGGACCACTGCGCCAGCTGTGAGGTGCAGGGAGGTGAGAGGCAAAGACCTTCAACCTAACGCACCTGAAGCACTTAGCACCGAGCCTGACACCCACTGAATTATGAGTGATGGCCCCTCCAGAGATGGGGCCTGAGGAGGAGGTGCTGGGGGCTAGATGCGGCCACAGGTAGCAGTGACAGGGGCTGGGGAGACT

>chr10(+):79434250-79434490

GGTGCAGAGGCAGGGCTGTGCAGGGCGATGGGAGTTCATCAGGTAGGGACCAGCCTGCAACCAGCTAAGGATGGACGGAAGAGCCTGGCAGCTCAGTCTCCTGACAGATTGAGGGCTGAGGGGGCTGGGCCTGCTGAAGAGGGAGAGGGCGGGAGTGGACATCAAGAAGAAGGGCCTCCAGGGCTTTGAAGGGGCCCATGGTGGGCCTTTGAATCCCTCCATCCGGGGTCAGTGCACCCGA

>chr10(-):98818762-98819027

CCCCACTTCTGGCCCCAGTCCTCTCTCTGAGGCAGGGCCTATAGTTTCCTGGCAGTTTCCCCTGCCCTGGGACCTGGAACCCTGCCCCTGGCCGGCCCCACTGCCTGGAAGGTCCACGTGCTCACTCCTTCCCGCCCACCAACTGCCCTGCCTCCATTGCTACCAGGCCTTCCTGCCTGATTAGCCAGGACAGCCACACTTCCAGCAGACAGTGGGAAACAAGTAACTAAGGAGGGGAGATGCCACAGCAAGGAACAGGTCAGAAC

>chr10(+):132246920-132247145

CCACCTCTGCCTGGGGACAGCCTCGCTGGTATTTTAAGGCCAGACAGGCTTTGTGAGTGGCACCAAGGAGGGAGCACACCCTCCTGGTCACTGTGCTCTTTGCACCTGTGTGGGGGACATTCCAGCAAATGAGTACCTGGCGAGGGGACCAGCACTCAGCAGTGAGGTCTTGTCTCCAAGTGCAAGGAGGGGCTGCCCAAGCAGCCCAGGGCATTCCATGGCTCCA

>chr10(+):71999176-71999391

ACAGCACCATGGGGCACTGGACACCCTGGCCCCCACATCTGGGTATAGCCATCCTCAGCCTGGCAGGAACCAGGACAGGGGCTCCATTTTGTTCTGAGGGCTACAGGTCCAGGAGGTGCTTTACTCATGCAAATCACACCCTGACCTCCCACCAAATGTCCAGAGGAGGTCACCCCTGGTCATTCTGTTGCCCTCCCCAGCCTAGGCCCTCCCTCC

>chr10(-):78379490-78379706

CTTGACAGGTGTCAGGAGTGTGCCTGGGGGTGAGAGAGTCCAACCGGGTGAGAGCAGGTGTGGCAGCAGCTCACCCTGTCAGGGAGGGTGTGCCCAGCATGACAGGTCACCTGCTGAGACTTGGCAATTTGACATCCTGTTAGGGTGCAGGGTGACAACGGGGGACCTGCCCTGCAAGTTGCAGCTGGGTAGGAATCTGAGTGTTGAGCCCTGCGTT

>chr10(+):81681361-81681626

TCCAGCACGGCATCTTCTCTCCGGGGGACTGGGAAACAGGGTGTTTGTCCTCACCTAGAAAACGGCCTCTACGCACACATCTGCTGGAGAGAATAAAGCTGCCGCGTGGCCCTGGGCTTGCTGGCAGGCAGGGGTGGCTGGTGGGCAAGGCTGGGGCCAGCCAGAATGCTCTGCTCCCAAGCAGCCTGGAACTCTCCCTGGAACAGGGCGAAAGCCCCCTAGTAGCCAAAAAAGGAAGATTCTCCCTGGAAACCTGGGCCTGTTCC

>chr10(-):47746462-47746743

GGCCTGTGGTGGATGACTGTTCTGTGGTGGATGGGTGCTGTGTGGTGAATGGGTGGCCTGTGGTGGATGAGTGTTCTGTGGTGGATGGGTGATCTGTGGTGGATGAGTGTTCTGTGGTGGATGGGTGCTCTGTGGTAAATGGCTGGCCTGTGATGGATAGGTGGCCTGTGGTGGATGGGTGTTCTATAATGAATGGGTGCTCTGTGGTGGATGAGTGGCCTGTGGTGGACAAGTAGTCTGTGATGGATGCGTGTTCTGTGATGGATGGGTTGCCTGTGGTGG

>chr10(+):132667943-132668162

GGCATTTGCTCTGGGCTCCTTCTTCTCTTGGCCTCACCTTCTAAGTGTGCCCTGGGCTCAGAAGGAGATGGGAAGCAAAACTCAGCTTCCTCCGTGGACCCCTGACCCCAGAGCCTCATCCATCTTGGCACCGATGGAGCTGCGCCAGCTCCCAAATTCCGGGCTCCCGCTTCCAGTAGCAACAGCAAAGCCTGGGACTGTGTGTGGTGCAGCAGCTCGG

>chr10(+):48792218-48792464

CAGGTGAACATCCTGGAGAGGCTTGGGGGTTAAGGGGTTAATGACATGGCTGCTCCTTCTTCCTCTGCCCTGCACAGCTTCAGCATTTCTCTGGTGGCTGTCGATAAAGGCATGTTAGCTGTGGGGACACTGCATGAAGGAGACATTCAGGGTGTCTGGGTCTTTCCAGCCCAGCCCTGCATCCATGCTGTCTGTGTGCCTGTGTCCCATGCTTGGTGCTTGTGCAGGGAGACTGGGGTGAGCAGGA

>chr10(-):70575545-70575764

GGGGACAGCCTGGAGCTGAGAATTGGAAGCTCTTCCCTGATCATCCGGCTCCATCACTCTTCTGCCCACTTCCCCCACCGGAGCCTGCTGTACCGGCTCAGGGCTCCGGCCTCTCTGGGCTGCTTGTCCTGGGCCCTGGTTCGCCAGAAGAGGAGCTGCCAGGACAGGGGTGGGCAGAGCAAAGTGTAATGGGGGCTCAGCTGGCTCCTGCGCCCGAGGT

>chr10(-):121983219-121983434

CTCGGTGACCACTCCCTCCTTTCTCACGCTCCCACCCTGAGCTTCAGCAGAAACCCCCTTCTGCAAGGATGGCCCGGAGCCCCAACTCCATAGCAACCCACGACAGCCCTGAACCCGTCCGACCCCCATAGGGCCCCTCAGCCCCCAAGGGCTGAGCCAGGGCCTCCCGGCGACCCTCTGCACCCCCCTCAGGCGGGCCTCCTCTGGGCGGGTTTA

>chr10(-):79990763-79990982

AGAACCTGGCAGCACCCCTAGAGCACCTGTGATTCAGGGCACCCCGTGCCAAGGGAGCAGCCTGGCCCGGAGCTGCCCTCTCAGCGGGGAGGAAGTAGAAGCCTCAGCCCAGCCTGGAACCTCCAAATCTGGGCGGGGAGTGTGAACCACCTTCCCTGCAGCCTCCAGTCTTTCCGCTTCCTTGTGGGGTGTGGACTTTGGAGGCTGATGGAAGGGTGCT

>chr10(-):128394603-128394822

GGTGCTGCTGAAGGGATGGCAGTGGGCTGGTATGACCTATTATGTGGCTGCTGGTGGGGGAAGATGCCAGGGCCACTGCTAATGTGACCATGATCGAGAAAGGACCAGGCCCATGAGCAGACGAGGGAGCAGCAAAGGAGCAGGTGGAGGACGCCCATCCTGAGCTAGCTCTTCCTCTCCCAGTGAAGCCCGCGCCCTGCATCCCCACCTGCTTGTCCTC

>chr10(-):104848054-104848273

CAGCCCCGTCGGGAAAGCGCATCTCCTCGGCCCTCCCCAGGGGCCCTGGGCGCTCACGCTCACACAGCCCGAGATGCTTTCAGAGCTCGCCCTGGAGAAGGAAGCCTCGCGCCCTGCCTCCCACGCCAGCTCTGTCTCTGCCTTCCTAGGCCTGGGGCTGGGGGCAGAAGGAAGCCGCCGCCCCCTTGCCCAACCTCCACCCTCCGCGCTAAGCTTTGAT

>chr10(+):132336035-132336254

CATCTCAGGACTGGGTGGGAGGATGAAGGGTCTTTGCCGTGCAGCTGGCAACAGAGCCCACAGCTTCCCACTCTTTCCTGCTACGTTTGTTTGGCCCTCACCCTGTTTCCCAAAACTGCACCCCTAAAACCAGGTAGCAGACTCTGATCTTTGGGAGCACGGGCTGGGGCAAACAGCAAGGATGGAGTCAGGGACAGGGTGGGGTCATGGAGGCAGCCTG

>chr10(-):1318391-1318610

ACTCACAGGAGTCCTGCAGCAACCCCACAAGGCAAGGGCTGCGACAAGGCCACGTTATGGGTGAGGAAGCTGGGTGAGGCGAGCCTGGACTCAGGGAAGCCGCCATCACCACGGCAGAGCAGGGCCCAAGCCCAGCAGCCTGGCACCATCCACTCCCTTCCCTGCTGTGTGACGCCGCCTGTCACAGGGATGTGGGAGCTGACTCGGAAGGGGAAATGAA

>chr10(-):127735265-127735530

TCCTGCCTAGGCTGTTTTTGCCACTCCCAAGGGGCTTCAGGGTTCTGAGTGAGCCACCGCATGGCCATCCTCTCCCCTCAGCTCCCAGCCCTCCCTGCCTCAGGGGTTGTACTCGGCCCCTTTTCTGGGTCTCTGTCCAGACCACTCACACAAGGCCAATCTACTCTAAGTCTGGCTGGGGATAAGGGGGTTGATGTCACGGGAAGCAGGAGCCATCTTTGTCCTTCAGAGCCCGAGGCCAAGGCGCCCTGGACCCGCCCCATTAG

>chr10(+):133976853-133977118

TCCACCTGGCCGGAGGCACCTGCCCAGCCTTGTTCCTCTCCAGCCCCGCAGAGCTCAGGCCCAGCATCCCCATAACTGGACAGGGTGATGGGACTCCCAGCAACCACAGGCACTCATCGCCAGAGGGGGACCAGCTCCTTCAGGCCTCTCCCTGCTGAACGGGGCTGCACTGGGCCGGATCCAGCTCCTTCAGGCCTCTCCCTGCTGGACGGGGCTGCACTGGGCCGATTCCGGCTCCTGTGGGGCCCCACGAGGCTCCTGGCTCC

>chr11(+):77847459-77847678

CTGGTCACTGCCCCTTCCCACACTGTAGCCCACTCCATGGAGGCCTCCCAGGCCCTCCCTGCCCAGGCTCTCTACCCAGTATCTCCCCCAGGACTTAAACTGCCCAACCCCAACTCAGCCTCCCCCATGTGCTCCCCACAACACATCCCAGCAAATGCCACCACCATCCATCTGCTTAGTATTCAAACCAGAAACCTGGATGCAAATTAGACCTCCCCAA

>chr11(-):75930064-75930283

TCCCTTTCACGGAAGCAGTGTGGGCAGGTCTTTGCCCAGCGCAGGCCACTGACCAGCCAGAGGTGGGGAGAGTCCCAGCTCACAGGCCCCCAGAGCCTGAGGAGGCCCCTGGGAAGAGTCTGCCAGTGAGGTGATGAACTCACTGTGCTCCCTCCAGGGATCTGGCCTGGGGCCGGCTCAGAGCACTGGGCCAATGGGAGTGGAGGGGACAAAGCACACA

>chr11(+):71246262-71246481

CAAATTGCTCTCCAGAAAGCCTGTCCCGTGCCCACCCAGGTGCGGGCTTGCTTCAGTGCCTGCTGGGCCTCATGTGGGTGATTGGCACCTTGAGGTGTCATGGACCACCCCGGGGGGACCACTGACACATCCTTCCCTCCCTGGGACCTCGTTTCAGTCTCTGGGGGCCCTGGCTGTCCCTGGCACATCTGGAGGTTGCCCCTAGAGTTGGGGGGGCAGC

>chr11(-):114131727-114131946

GCTGTGACATGGGCCAGGAGGCAGGCTGGCAGACGTCCAACCAACTTTGAGAAATGCTGCCTTTCCCCGAGACATCTGCTCCCAAAGTGTTTCTAGCCAGCCTGCCCCCTGCAACCACCCGAGGAGTTCCCTGGCAGAAAGCAGGTCTCAGCAGCAGTGAGGGGGCCATGCACAGCCCTGCCACTCTCCTCTGCCTGTTTGTCCCTGCACAGCCCACACT

>chr11(+):95014672-95015047

GGGCCAGCGTCGGGGCAGGGGTGATGTTGCTGTGAGCTCCCCACATGGCCCTGGCACTCAGGGGTGACCTGGGGCTCCCCTCTGCCTAGCTCATGGCCCTTCCCTCGGTGGGGGGCTTCTGGGACCAGATCACACGTGCAGGCCAGGGCCAGCTATCAGGAGTGTCAGGTTCAGCAGTCACCCTGATGTGGGGCAGACCTTGGGGATGTGGCTCCAGGGGGCCCTACACAGAGCCTCCTCCTGAGGTGCAGGAACCCAGAGCCATCAGCGGGGTGGGAATGGTGGCCACTGGCCAGGTCCCTGAAGCGGGTGCCACTCCCACTTCCCACCCCAGGCCCCCAAAGCATGGCCCCAACTCTGCACCCTGGGCCTGGGC

>chr11(+):77425044-77425263

AGGGGCAGCTGGGAAGAGCAGGGTGGGCCTGGGTCCACTAAGGAGGGGCTCTGGGGAGTCTCCCAGGGAACCCGCAGTTCTTCCAGGCAAGAGCCTGGCTCCAGTGGGAGCAGCTGTGAGGGTGGAGTCCCAGGGAAGGACCCTCAAGCCTGGGTGCAGGAGGGGTTCCTAGAGCCCTCAGATCACACCGGGGGTTGGGGGGTGTTGAACCTCTTTGCAG

>chr11(-):115397919-115398138

CTTCCTCCCTGCTGGCTAGGAGGCTGTCATAGTGACTGACAGCATAATGGTGGATCTTCAGCTCCACCCTCAGAGCCCAGCGTGCAACCTGCCATCCACTGGGTTCTGAGGGTTCCTCCAAACCATCTGCCTTGCAGTACCTAGATCCCTGCCCTCCTTTGACTGCCAGACACTGTTGTTGGATGTACCTCATCTTGCCAGTCTCCTTTGTGGGGCGTTT

>chr11(+):80744098-80744317

GCATCAGATATATGCTCCCAAGAGTTGAGGAGGGGCTTACAAAGGGTAACAGGAAAGAGGTGGCAAGTGCATTCAGTGGAGAGTTTGGTTCCACTCACCTCTTGGGCCTCATCCCCCGGGTCTGTGCCAACCCATCCAGCCTCACCCTGGATCTGTCCTGGTGCTCCTGGCTCCTGACACAGTGCACTGCCCATCCTTGCTCCTCCATAGGCCTTCCTCT

>chr11(-):135335466-135335731

GAGCTGGTCAGAGCCCACCCGGCCGCCTTCTGAGACAGGCCCACCCTATGGTCCTAGGCTTCAGGGTCTGGGCTGGCAAAACCTGTCCCCAGTGTGGGGTTGGAGTTGCCGACCTCTGGCTCAGGCCCTAAGAAAGGGAAAAGTGAGGCTCAGTGGGTGAAAGGCCTTGATCCCGGCGCCTGGGTTGGGCCAGGATGACCTCTGCCCCACCTGCTTTCTGAGAAGGATAGGGACACCATCCGTGCATCGGGCACAGCTGGAACCTA

>chr12(-):104173891-104174156

CCAGAAAGCCTGCAGGTGAGCGGAGAAGGCTGGCCCCAGAGTCATTTATTGTGGCTGCCTTTGCTGAGACCTGGTTTCACCTGGGTGAGGTGGATGTGGGCAGAGGAGGAAGAGCTGCTGCTGTGAACACAGCCAGCCTGAGAGGCCAGGTGCTCAGAGCAGATTCAGCCTCTTTGCAGAGAGCATTTTTCTGCACCCCACCTCAGCTGTCTGAAGCTGCAGATCCTGCGTGGGCTGTGAGCCAGGGACCCTTTCTATCTGGGGCA

>chr12(+):107768836-107769055

GCAGTTGGCAAGCTAGGATCTGGGCCTATGAAATGAGCCTGCTGAGTCCCTCACCCCACCCACGGTAAAAGGGGCTCTGACAGAAGAGCCGGGACCTTCCCAGATGATCCTGGGCTGAGTCTGCTGGTCAGGGATCTGGGAAGGCAGGAATAAGGAGGTAGGAGCTGGTCAGGAATCAAAGGGACTCCCTTCATGAGCTGACCAAAGGCCAGGGCTGTTT

>chr12(-):114834668-114834933

AACGTGGGCAGACAAAATGGAAGGAAAACTCTCTCCTGCCATTTCACCAACCCAATGGGCTGTCCTGAGGCAAGGCTCTTCCTTCCCCTTTTGAAATGGGTGTTGAAGGTCCCGGCTCCATCTCTGCCTCAGGCCCAGCAGACAGGAGTGTGTGCTGGGTGGGATGTGCCCATTCCAGCTCCAGGAGGAGACACAAGCAGCACCCCAGGCTTCCCAGCCCAGCACTGCTCTGAGATTCCTGTTGAATCTGGAGACTGGGAGACCTC

>chr12(+):130367471-130367690

AGTAGCGGCTCACTGCTGGGACACTGCTGTCTGCTGAGGGTTGCTCACTTGGCTGTGGTTGCTGCTCTCAGGCCTCTCATGGAGCCACCGTGACACACCCCCCGTGACACACACACCCCGTGACACACACCCCCCGTGACACATCCCCCGTGACACACACCCCGTGACACACACCCCGTGACACACCCCCCGTGACACACACCCTGTGACACACACCCCG

>chr12(-):2284650-2284869

GTCAACACCATTGTCTCTGAAAACACGCCCCTGCCCAGCTGAGCCCAGGGAGGCCCAGAGGACAGTGCCCCAGCACTGCCACTTTCCTGATGCCCTTTATCATACCCTGCCCCCAATCCCCAATTGCCTTGACATCTGTGAAGCAAGGCAGGCGCTGGCCAGGAGGCTCTCTCTGATGCTGGCTCTCCACCTGTCCATGCTTACCACCTGTGGACTCCAG

>chr12(+):108118311-108118576

TCACAATTAGCTAAGCCCGGGGCACCAAAGCCTTTAGCAGCGAAACCGCCGCTTCCGACCTCTCGGACCTAGTGAGCCAGGCAGAGCCTTTAGGCCCACGCCTGGGCACCACAGCCAGCCCGAGGCCCAGGAAGCCGCTCCCAGGCTGCGCGCGGCTGGCGCTGGGCTTCCAGTGCCGGACTGCCAGAACAGGCTGGGAGCGCTGGGGTCTTGGCGCTCTGCAGCCACCAGAGATCTGGCCGAGTGGGTCTGGTTACCCCCACCCC

>chr12(+):63751404-63751623

TCAACCTCACCACTGACCCCTTCACACTGTAGACTTCCTCCCAGGCAGCAGGGAAGAAGGACTGAAACTAAATCAAGTTCAGAGTTTTCATCTCTATCTCAAACAGAACATTCTACTTTCCAAATGAAGACAATGTTTTCACTGTATTCTCCCTGTAGGGCTCTCTGTTATCTTAGAGGGAATGAAAACATCATCAACTTGTCAAGTTTTCATCCAGAGT

>chr12(-):124504814-124505034

TAGGGGCAGCTTGGGCAGGAGGTGGCTTTCCCTGCTGGGTAAGTCCCCAGGGCCAACAGCAGGAGAAGCCCGACGGGGAGGGTGTGGCTTCCCTGCTGGGCTCGGGTACAGGAGAGGCTGTCCTCCCTACCTTGCTTGTGTGTGGCAGGTCCTGGGTTGTGGAGGCAGCCCAGGAAAGCCGTTAGCCAGGGACAGCACCGACTCACAGGCTGCTTATCCTC

>chr12(-):52457025-52457244

AGAGACAGGACCAGGCAGGGTATAGAGAAAAGGCACTCCATGGAGGAGAGAGGCTGCCCCCAAACCTTAGGTATGCGGCAGGGCAGGGCTGAGATCCTGAGGGTGGGAAGTAGTGGGCCAAGGTAAGCCACAGGACCCAGGACCATAAGCCAGACTCTCAGTGGCCCTCCCCAGGTGCCCTTCCATCTCACAGCATCTGAAACTCCACCCCACCTAGGAG

>chr12(-):76146929-76147174

ATTCAGCTGCTGCCCCAACCTGGGACCTCCCCAGGAGGTTGGAGCAGAAAGGGTTCTCCTTGGGGTGGTGGTTCTCCTCCAGGGTATCAGGACACATTATCTGCACTGCCAGCAGAGAGGGTGTGTCTGGGGAATGGGGTGGTAGGGGCTTGTGTACTCTGTCATTCCCTTTCTAACCCCTGGACCTCCGACAAGCCCAGGGTGACAGCTGGTGCTAGGGGTGTGAGGTTAATAAATGGCTTATCT

>chr12(-):50379345-50379590

CCAGGAAATCTGGTCCTTCTCCCTCCCACCGTTCATCCCTCCGCCCCAACTCCCCTGCCCAAGAAAGCCTGTCAGAAGGCTTTGCCCAGACCTCAGCTCTCTTTCTAGAGGCAGCTCAGGTGAAGAGCAAGGTAGAGAGCTGTGGGTAGGGGATGCAAGGTGGCAGCCAATGGGAAGGCGGTGCAGGGTTACCTTTTCAGAAAGAGATGTGGCAGAGGAAGACTGGTGGCCTGCACCTCATCTGCG

>chr12(-):107742853-107743075

CTCCCGTGCCCAGCTGGCAGCTCCCAGCAGCAAGCTACTCACACGCAGGTCCCTGGCTATCTGCAACTTCCTGTGGCCCTGGTGGTCTCCTGCTAGAAGCTGGAGCGCCTTTAAACTCTTTATACTCACAAGGCCACTGACTGGTCAGCTTCCCTCTCTCCCAGCATAAGAGGGAAAAGAGTGCCTACCTCCTGCCAGGCCCACAGAAGTGAAAAATAAACAC

>chr12(+):106136091-106136310

TATCTGTGAAAGAGCTTCAGGGCCCACATACACTCTGCTGGAAGTGCAGCCCAGACCTGCTGCCCCAGTTCCTACTGTGTGCCCACTGCTAAGCTGGGCACTAAGAAGTAAACCAAATGATCTGTGTGATTCACAGAGCATCTGAAGCAGCCTCCAGAGGGGCTCAAACTCATCTCAAAATGCATGGAAGAATCTGGGGGATCCTGGCTCTCCATGGGGG

>chr13(-):98327745-98327964

AACTGTAAAGACAGGAAGAGCGATGGGAAGGCTGACTGTCAGATGATAACCAGAGAGACAGTGCAGCGTGTACAGAGGCACTCACTCATGTCACCACGGGCTGCTGAGAGAACACAAAGACCTGTCTTAGATATGGGAGTTGAAGGAGATGAGCAGGCGAGCAGGCTGGATTTTCCTTGGGCTATAGGCCCAACTCAGGCCCCACCCAGCATTACAGCAT

>chr13(+):67172599-67172841

TCACCAAAAATTGCAATATCTCACACTAAATATGCCAAAAGAAATGAAGATCTTTCGGCAATCACAGTGGTTGGCTTTCCCAACTCTATCCAGGTTTCAAACCATAGGTCTGCAATAAACCATATGTTCTACAGTATCTTGGATACTTATACTGCTTGAACCAGTATGGAGCATGTGCTTGATTAATAACTAACAAATATTATACTTGATAAATATTAATGGATAGGGTGCAATTAAAGCTTG

>chr13(+):19163242-19163461

TAGGCGGTGCCGCTGTGCTTTGATAAAGGCCCCTGTGGCTGGCAACAGCTCTTACCTTTTGTCTCTGGGTCCTGGCGTGGGTGGAATTCCTGGGCGTTCTGTTGGCTCATCTGGGGCCATCAGTCCACTTTGAACTACTCACTGTCTCCTCTGCCTCCACCAATTAGAAACCCTTCCAAGGGACAGTGAGAGCCAAAGCCAAGAAAAGCCTTGTTCCCTA

>chr13(-):66598628-66598847

TTTCCAGATGCCTGGAGCAGCTGTAGCCATTGCAGAGCTGTCAGAATCCTGGGCCCACTAAAGCAGGTGCAGCACTGGGGCAAGGCAGAAGCCTGAGGCCCACCGTGGCTGATGAACCATGAAGCCTGGGGCCTCTGTGGGCTGCCTGCTGCTGAAGATTGTCCAGGGCCCAGGGCCACTACATTTTGCCTGGCAGTGGTATGGAATTGGCTCACAATTC

>chr13(+):111134991-111135302

AAGGAGGTTGTGGTGAAGAACAGGGCAACTGAAAGCACAGCTTCCCAGCAGGGGCAGACACAGGTGGTGATGATGCCTGGGTGTCCTGGCAGGGGTATGCTGAGGGGCGTGAGGGTAGGACATGAAACCTGGCAAAGGAGGTTAAGGCAGCTGGCAGAGCCGTCCCACGGGGGTAGGGCTCATTTAGGACAAAGAAATGGGAGAAGAGCAAGATGGGCCCTGACGCTACAGGCCCCACTGAATGAGGAGAAGGGCAAGCTACAGCAGAAGGGTGAGGAGCAGCCGGGGCTGGAAGCAGCTACATGAGCAGGA

>chr13(-):36110897-36111116

GGGGCCCATCCCCGGCATCAGGGCAGCTTGGTTCTTGCAACCTCTTGAGTGCAGACTCGGCCTCCTCAAACCCCTGCAAGGCCTCCTGCTTCAGGGCAGCTCTGAGGGGATGGCCCGAGCCGCGTCCTCACCAGCACATCCTGATGGACCCAGCCAGCCTCCAGCCTCCCCTAACCACTGGCACTGGGTCCCCTACCCACCCGTGACAGGAGCCCCACTC

>chr13(-):92786246-92786468

GTACAGTTGTGAGGGAGCTTGCTTGGGAAAAGCTTGAGGGAGTGGCCTGGGGCTGAGTCCCGAGGAGTGGGTGGCATCTCTGGACCCTGTGCTAGGTGCTGAAGGCCTGCAGCTGAGAGGAGGCTGATGCCTGAAGCCAGGGTCCTGTCAGCCTGGGCTGGGCAGACTCAGGCTGTCTGTAGCCTCAGTGCAAACGATGATAAATGATGAAGAGCCGGACACC

>chr13(+):18558567-18558786

TTTATTTCCCGGCTCTCTAGTTCTCTAGGAAAACCAAGGGGTTTTCTTAGCTCTGTGGCCATCCAGGGGTGAGGGGCTCCTCACAGGGGCGCAGACGGGACTCAGGGAGGGTAGGGCTGCCCCCTTATGAAGGTGCTCCTTCCTTGGGAGTTCTCCTGGGTGTCCTGGAGCTGGCTGTGGGCAACAGACCCTGCCCCAGGAAGACGCAGGTGTGGTCAGC

>chr14(+):90896693-90896912

AGGTCCAGGAGCTGTGGGGAAGCAGAGGACAGAGCACAGCCCATGGGACCCTGATGTGCTGGGCAGGCAGGCTGAGTTCCAAGACCAGCCCCCTGCAGGGTGCTTGGCTCCATCCCCTCCTGGTGCTCTTGGACCTCAGGCGCCACCATCCACCCTATGAGGACACTTCAGGCCTCTGCAAACCTGGGGAGGACCACAGGGTCGTTAGTGGGTTGGTGGC

>chr14(-):98674295-98674606

ACCCGGCCTCCCAGACATCCCGGGCCTCTGCCCTGTCCACAGGCCGTCCAGAGCCTCACACACGGTTTCCCACCCGCTGGAATCAGCTGGGCTTGGACCCAGCACACCCTCAGGTCCACACAGCTTTCCAGGCCCTCGGGAACCACAGCCCCACCCGTCCAAGCCCCTTCCCTGGGGGTTCCCTGTGGGCCCTGAAGATACTCCTGGGAGCCTCCCCTTCCCCATCGTGTGCCCCGGGGCCCATCTGCAGCCCCCATCCATCCAGGGTGTTCCACGTGGAAGGACCAGCTCACAGCCAGGTGTTCACGCCTC

>chr14(-):99951462-99951681

CCTCCCACAGTCAGCCCTGTGCCCAACCCTTCACCCAGTGTGGGCTGGCCCCCCACACCCCTAGGTGCCCCCTGACACACCAAGTGAGACCTGCTTGAGATGCCAACACCACCAGCTCACACCTGAGATCTGTGTTCCTTTATTCCAACAGCCTGCCCTTAGCCAGCTGCCTGCCCAGCCCAGGCTCCTGCGTCCTGGGCCCCCACCCGTGCCACGTCTT

>chr14(+):99671134-99671491

CAGAACCGCAAGGGCTCCTGGGGTCTGCGGGCTAGCTGGGGGCCTGTCTCAAGGGCTTCCCCCTCTTCCACGCAGTATCCTGTGTGAACCTGAGGCAGCCAGGCCACACGGGAGGGCCTGAGAGCGCCTGGAGAACAAGCTCCCACCTGGCATCACCTCGGCCATGGCTTTGGCCTGTACGGGGGTCCCCAGCCAGGGGCCTGAGGATGTGGGGCGCTGCGGCCCATTGTTCACGCAAACCCACTGTTGGGCTCAGGCTGCTCCCTCTCGCAGCCGCGCCAGCACCTGCTGACCCCGCTTGGCTGAAGCTCGGCCCCAGCCCAGCCTGGGGGGATCCTGGGGCCTGTAAGCAGGCAAG

>chr14(-):13719769-13719988

TGGGAACTGTGCAGCCAAGGAGACTGGGCCGAGGGCAAAGGTTTCTGCCCTGCTGCAGCTGCGGGGCTGACTGCCTGAATTAGGCGCTGAGGCTGCGTTGTCCCCGGTGTCAGGGCTCTGGTGCAGGCAAAGTGCCGGGTTGCTCTGCTGCTGTCGTGCCCTTGTACAGGTGGCAGCTGCAGCTGAGCTCTCAGTAGAGGTCGGCAGGGTTGGTCCCAGA

>chr14(+):98727629-98727866

CTCTCTTTGGCTGTCCCTGGGATGCTGGCTCCGGGTCGCTGCAGGTCCAGCCTCCTGTCCGGCTCCTGAGCTCCCTCCGTGCTCCTGGTTCTGCAAGTCACTCTGCTCCCTCTTGAAGGTCTGAACCTATCACAGGCCTGTATGTCCCTGGATGCCCTGCTGTGCTTGGGGCCCCTTCCCTTCCTACCCCTCTGGCCTTTTTGACACCCTCACTGATACCAGGGGGGCTCCCAAAGCT

>chr14(+):99708144-99708363

AGGGATTGGAGTCTTAGGCATCTCTGGTACAGTGGGGTGCACGTCTCAGGTGGAGGAAGATTTACGGCTCAAGACAGGCCCCAGATCCCCTCCCAGTGGCACCCATGCCACCTGCTTTGAGGGGTTGGATCTTCCTGCTACCCTCTTGGATTCTAAGTGGTTCCAAGCTTAACTTGAGACCTTCCCTTCAAATCTAAAATTGGCAAAAAGTCACTTAAAA

>chr14(+):99614135-99614429

CCACCTGTCTCTCTGCCACCCCCTCAGGAAACACCCCTGCCCTGAGCCGCTGGGGTCCCCACAACTGCTGCTCCCCACCTGGGCACCCCCACCGCCGTCCCTGCCCTGAAAACAGCCCCTCCACTCTGCTGGCAGCCAGGATGCCCTGGCCCCGCTCTCCCTCCTCCAACCCATTGGCCAACCCCATGCCTCCACTTCTGAGAGCCCCTCTCACCCCCAGACTGCCCCTGGTCCAAGTCACCCTGACACGCCCAGATTCACGTGGCAGCTCCTCCTCGGTCTGTGTCCTGGCCCC

>chr14(-):99432304-99432646

GCTGGCCTCTGGCTCCTGCCCCAAGCCTCAGGCAGTGCCCACCTGGTGCAGCTCAGGGAAGGGGGTGAGGCTCAGAAGTGGACAGGAGGGTCAAGCCCGCAGAGCCTCCAGTTCCAATGCCCCAGGAGCCCGCTCCCTCCCGGCCTTCTCCATGCCCAGGAAGCTGAGTGCCCACTCCCATCCGGGCTCGTCGTGGCTGGGCCTACAACAGGAATGCCCTCATTGCCTTCTCTCCATTTGGCCAAGCTCTTCTGTCCTGGGCTGGGCTAGGAGCTGCCTCTTCTGGGAAGTCCCCTGGGTTGCTGGGCCCACTCCCAGCTCTCCCTTAGACTCTGGCCCGCTC

>chr14(+):93842768-93843033

TCCTCAGAGAACAGGGCTCCCACAAGGCCTCCACCCCCAGGCCCACAGGGAGCCTTGCCAGGGTAAAGCTGCCTGTGTCCACTAGAAGCCTGGGCCGGGCCACAGGACAGTTCCCCTGCCTCCCTCAGTCTCCACAGGGCAGGGCTGCATTTGCAGAGAGGGCTCTGCTGCTCCCTTCGGCAGAATCCACAAGCCACTGAGTGCTGAAGCCCCACCCACTACGTGCATGGGGGGGTGCCTGCTGCCACACGCCTCCAGGGCTCCCG

>chr14(+):98645894-98646113

AGGCCACACACATGAGGAGCCAGCTCCCATGCAGAGGGCCCCCTCATCGCCCCACATGGACACACCTACTTTCCATTGTCACCTGTCAGCATGTCCGGGCTGTGCTCCAGTCCTGACTGTGGCCCTGATCCCTCTGGGACTGATCCAACCCCTCCCCCTTCTGCACCAACTACAGGAAGAAGGGCTTCCACTAGGCCAGCACCCAGGGCTCCTCAGGCCC

>chr14(+):98475032-98475271

TGTGGATGTGTCTTTCATCTGTCCCTTTGTGGCCCTCAGCCCTGTCCTCCTCCCTGCACTCTCCCTGGGCCACCTGCCCCTGCCCTGGGGCTTCACGTCCCCCTGGGCCACCTTGGCCCCCAGACCCCATCCCTGTGCCCCACATCTGCAGTTCCATCGTTTCTCTGGTATCTGCCCCATGTCCTCCGGCCCCCAGATCTCTCAGACTTGTTCTCCCCTGTGCACCCCTCCTGGGGGCCC

>chr14(-):99675341-99675560

AGATCCAGGGTCTTCGGGCAGCTGGGGCCTTTGACGTGGGCAGCGGTGCTGGGGTGGATTGTCTGTCTTCACTGAGTAGCCATCTCTGCTGCAGCCCTCACAGTGCAGCAGCACATCGCCCTGCCGTGTGCATGTGGCCCTGCCCTGTGTGGGACCCAGGGGCAGCAGGGCCCTGAGCACCTGTGGCATGTTTGCTGCTGTTGCTGTCTGGAGGCCCTGC

>chr14(-):99607730-99607949

TGCCACTGCCCCCAGGGCTTCTCCGGGCCTCTCTGTGAGGTGAGGTCTGCCTGGTCACCCTGCCCCACCTGCTGCTCTGGGAGCTGTAGGGCAGGCCTCGTCCCCTGACCATGGGGCCTGAGTGACCCAGGGGTGCTGCAGGGGAAGTTGTCCCCAAGGCGTCCCAGGCTCAGCTCTCCACTGGGTGCCAGGTGGGCAGGCGGGGCTGTCACAGGTCACC

>chr14(+):96839509-96839728

TCCAGCCGAGCCTGCAGCCCAGACCGAGCTAGACTGGAAGGGGTAGAAGGGCATTCAGGTAGGGACACAGCCTGGGCAAAGGGGCCCTGACCTTTGGCAGCTGCGGGAAGATGAGCCCACCTGAAAGGGAGGCTGCATTGAGTGCAGTCAGGACCTGGGCTGTAGGGCTCAGCCTCTAGACAGCAGGAGCACAGAAGGGCGTGGCGGGGTCTCCTGCCCG

>chr14(+):99939489-99939708

CTATGCCCTCCCAGCCGCCTGCCAGCCAGGTGCTCCCTTGGGTGCCCACAGACCCAGATATCCCCTAAAAGGGAGGGGAATCTGGAATGGGCTCTTCTGCCTTTGGGCGTGCCTGCTTCTCTGTCCCTGCCTTGGGGGCTGCTCTGCCCTCAACCTTCACTCTCTTCTACTTGGACCTATGCCTGGCCAGTGAGGCGGCTCTGAAATGCTATCTGCTCCA

>chr14(+):97410416-97410635

TCAGCAGGGCATAGGGGAACTGGCACAGGGCAGGGGCCACGGGAGAGATAACCCAGCCCAGAGCCAGTTCCCGCTTGCTCGTCCTGACTCACCAGGAAATGCCGGGCACCTGCACCCCTGCCTGCCAGTCTTTCTGTCCCAGTGACCTCGGGGTCTGCTGGCCTGGGGTTAGCAACAGGCAACTAAAGCCAAGCTGGGCATGGAGCCCTGGTGAGAGGGT

>chr14(+):99989847-99990184

GCGTGTCCACTACCCGCTGGCCCTGTGCTGGGGCCTGGTGCCCAACCCAGGCAGAGACGTCTGGGGACAGCCACACTTTGGGGAGTTAGGGCCTGGGAGGAGCAGGGAGGCCTCCTCAGATGGGCTTGGGTTGGACTTGGGAGCTGGCCTCGAGGGAGGCCCGCAGGTGCAGCAGCCTGGAGGCAGAGAGCTGGGTGTGTCAGTGCCTGAGAGGGCCGTGGTGGGCAGCTCACCAGCAAAGGGAGCAGAAAGGGCCCGGGGGAGAAGTGGGCAGCTCAGAGGGAAGAGGCGGGGCTGGGATGCTGAGCAGGGTGTGGACGGAGATGCAGCTGGGTGCA

>chr14(+):94969804-94970174

TCTTTCTCTGGTTCACACAGCACCATCGACTTGGCCCCCAGCATGTCCTCAGCCACCGTCATCATCTCTCCATCCCTCAGCCACCGTCATCATCTCTCCATCCCTCAGCCACCGTCATCATCTCTCCACCCCTCAGCCACCGTCATCATCTCTCCATCCCTCAGCCACCGTCATCATCTCTCCATCCCTCAGCCAACGTCATCATCTCTCCATCCCTCAGCCACGATCCCAGTTGAGGTCACATCCTACCCACAGGGGCCCTACTGTGCCCCAGGTCCCTCGGCGACCAAGCTCCGGTCCTCATCTCACGTTCACTCTGAACCAAGGCAAGCTGTCTGGACTTCCACCCTGACCATGCCACTGCCCTGCTC

>chr14(-):31949199-31949418

ACTTTCCTCCGCCCGCTGCTGGCCTCCATGTAGCCGTCCTCTGCGGAGACGGAGCTGGCCTTCCTCTCTTTGGCCATGCAGGGCCCTGCGAGAGGAGCTACCTCCTGGCTCTGTTCGCGCTCCCTTCCCAAGTGGAAGACCCGCCTGGGAGCCGCGTGCCGCGAGCTCTCCAAGACCCCGCGCGCTCTGCGCTTCACGGGTGGCTCGGGCGGAGGCAGCA

>chr14(-):99190775-99191010

TGTCCCCAGGACCCCAGAGCCCAGATCCAGCCCCACCTTCCTGCCTCACCTCCCGGCAGAAAAGCCCTCAACTTGTCTACGCATGGGGGATTCTCCCAGCCACACCCCACTGCTCTCTGCCCGGCAGAGGGGTCTCAGCTCCCCTCCCACCCTCACAAGCCTGGCAGTGGGGTGTCTCACATAGCCTTTCCCCGCCTGCCCAAGCAGGACCCTCCGTGATGGCGGCCCCCTCCCTC

>chr14(+):99423748-99423993

TCTGCACAGCTCTGTCCTGGCCACCTGGGCCCATCTCACTCACCTTTGCATTCAGACACTGCCCCACCACCTGACAGGGCCCCTGCTGCTCCCACCTGGACGCACCCCCAACCTGCACAGACAGAACTTGGCCAGCCCCACCGCTCTGCCTCCCAGATGGCTGCCAAACCCTCCACTCCTTGCCCACCTGCCCCCTCCCACTCCCTCCACGTGGCCCTGCCAGGGCTCCCCAGTGCACCCCACAAC

>chr14(+):99952618-99952883

AATCCCAGAGACTTGGCCCCTGTACCTCATCCCAGGCCTGCCCAGGGGGACCTAGGGGTCAGAGGCGGATGGCCTGGCTCCTGGCCCCTAGTCTTGTGTCTGCTGGGGCTTCCTGCTCGTAGCAAGGGCCTGTGGGTTCCAGCTCTGCAGCCCAAGCCTGGGGGATTGCATGCCTCACTCATGGGCTTACAGGGCCCAACCCCTGACGCCTGCCTGGCCCCTGGTGCCAGCTGGACCTGTGTCCCTGGCTGAAGCCGGGCTGGCGC

>chr15(-):33535415-33535680

TTCTCACCCTGGTTGCCCTTTCCTGCCTAGCCACCTCTAGGCTGGGTGAAAGCCCCTGAGCCACACCACCTAAGGCCACTCCAGAGGCCCATGCCCTCTCCTCAGTGGCCTGGGCTCTCATCCTTCCCTCAGCCTCCTAGTCACCCTTCTCTCCATGCCTCAAAGCCACACTGGACCTTTTCCCTGCCCCAGAGATGCCACCTCAGACCCATGTGACAAGCCTAGAGAAGGGGTAGAGACTGTCCCACCCAGCCTTCCAGCCGCTG

>chr15(-):63837252-63837535

GCTCCCGGCCTCCCTGAGTGTGCCTGAAACCACCCACCTGGGCCCCTGTCAGGCTCTCCCTCCCGGGCATCATCCTTGTGCTGTCCACCCTGGCCTGCTACCCTGGAGGCTGGGGTCATGTCCATCCTGAGATAGTGAGGAGGAGGTGAGGGGACAACTGCTGGGACGGTCTGGAGCTGACTGTCACTGCAGGTGGCAGGCACATGTGGAACCCCAGGGAAGGCGTGGCAGGGGCTCATCTCTATTCCTGTCACACTCTCACCCCAGCCACGCACAAGTGAGTT

>chr15(-):95913516-95913873

AGAGGGTTGTGGAAATGCCGTGACCGTGTGTAAATCCTTTACATCTAAGTCCTTCTCACTGTTGGCACTTCCCATTCCCTTCAGGGTTTGTGGAAGACACGCCTGTTTTCTTCCCTGGGCTAACTGTGAAGCTCAGAAGAGGAAGAAGGGCCCGGATGGCAGATGAACCGCACAGGGCTGTGTGGCCTCAGCCCCGCAGCTGGGGAGCTCAGTGGCCTCCTGGAGGAGAAGCTGACTTTGGAGGACCTGGAACACCATGGCTGCTGGGAGTTGGGGCTGTGCGGGAGGCTTCTGTCTCTGTGTGGCGTTTGGTCATAACCACTGGACTTTGGAACATCCTGCTTAAAACCACAACTTT

>chr15(-):46854444-46854767

GCAATGCTCTCCTAATATCCATACGCAAGTGTGTTTATGACACAAATTCACTAGTCTGTTTAAAAATGAATTCTTTATATTGACTGGTGTTCCACATATTTCAGTAATTTCTGTTATGAGAGGACTTGAAATAGCAAATTGCCACACAGTTAACTGGATAGACCACGTACGTGGTGATCATAACCACTTGGTACTACACCCAGAAACTCAAAATTGTCTTTCTCCTGATGAGATATGGGTGTCCTTTTGTACGTCTAGGCCTAGGTAACCAGTGGAGTGATTATATTAGCAAATGTGTTTGTATCCGGAGTCTTCCTGTCATTG

>chr15(-):68562381-68562600

CATGCTACCTGCAGAGAGGACCCAGGGCACAGGCCCCAGCCCCAGTGGCCGAGATAGGGGGAGGGTTGCCCCAGGGACCCCCCCAAGTACTGAGGCCTGGGCATGGAGATGTGGGAGACCCCTCTCAGATCCCTGCTCCTGACACACACAGGCAGGGTCCCTTACTGCCTTATCTTTCCTGGTGCTCACACCTGTCCCCTTCCACAGCTAATTTCCATCA

>chr15(-):19429934-19430153

CCATCAGGGCATCATCTCCTATTTTGACCATGTCTACAGCCCTTAATTATACCCCTTGCATCCATCGTCTGTCTTCTGCCCCTTGGACCCACTATTACACGGTTTCCAAACTCTCAGGCAATGGCTCAGTCTCACCCTGCTGGGCTCCTGTATACACTGTCCCCTGCCCACAGTATGAAGTCCAAGGGTTTAGTGGGGCCCAGCAGGCTCTCCTCCCTTT

>chr15(-):67226738-67226957

GTCAGGAGGAGCCTGAGGCCCAGGAGAGTGCCTGGTTTACTCAGGGAGGAGGCAGTGAATGCAGAGTGTGGTTGGAGCCCAGACCCCAGCCTTTGGAACTGGCCTCCTCTCCACCCTACCGTGCCCTGGTGCCTCCTGCCCCTTGCCAGCAAACCCGCCCATTTGGTTTCACTCACCCCGTAGAAAGGAGCTGGAGCCTGAGCCCAGTATCGGAGTCCTC

>chr15(-):46855566-46855785

GGGATATAAAGCATTAAATATATGATATATAGCTATATCTATGTATGTATCTAACAGAGAAGTTCAAGTCACTTCAATTAAAGAAACATTTTTGAGCATGGGACCAGCCTCAGGTCTTATGCTGGGATGCAGTAGACAGGAGATGGGGAAATTAGAAAAGAGAACTGTGTAATTGAAATGACGTGGGCTGCACCCTTAAGGAACTTATAATTAATGATGA

>chr15(+):33535369-33535634

GGAATGGCCTGGCCTGATGTCTCCGGGAAGGGTGCCCACTGGGCAGCAGCGGCTGGAAGGCTGGGTGGGACAGTCTCTACCCCTTCTCTAGGCTTGTCACATGGGTCTGAGGTGGCATCTCTGGGGCAGGGAAAAGGTCCAGTGTGGCTTTGAGGCATGGAGAGAAGGGTGACTAGGAGGCTGAGGGAAGGATGAGAGCCCAGGCCACTGAGGAGAGGGCATGGGCCTCTGGAGTGGCCTTAGGTGGTGTGGCTCAGGGGCTTTCA

>chr15(-):35276753-35276972

CAGGGCCGCCACACTGCATGGCTCCTGGGGTTAGCATTTCATAGGCCTTCATGTGCTTGGTGCCTCCTGGGGGTCCTGCCATGCTGTGGCCCTGCAGGATCCCCTCCTGGCTGGGGCTCCCACTGCCCCACTCCCCGGCACAGGCAGTGTTCCTTCCTGACAGCCCCTGTTCTAACAGACCCTCTGGACATGGGCTCTTTCTGCTGGGGCCACGTGCCTC

>chr15(-):66832373-66832592

TCTCCACACAAGGCACAGAATAGAGTCAGGCTCCCTGCATGAAGCCAGGAGCTGGAGTGGGATGCTACTCCATTGAAAGGGGCTCAAAAAGACTGCCACTCTGCTGCCCGGTGTAACTGTTCCCAGCAACATGGGGCCCAAAGGAGGCAGGTGGACAGACACAGCCTCATGAGCAGGACCTGAGCCAAGGCTCGTGCAGACTCTGGAACTGGGTATCTCC

>chr16(-):68920241-68920552

TCCAGGGATACTGGGGGGTCTGGAGAAGGGACAGCAACTCCCAGGAGATGTGGGGAAGCTGTGGCCAGCAGGGGACAGCTGCAAAGCAGGAGATGGAAGGCTGAGGATGTGTCTGTAACAGATGGCCCCTGCTTTCCAGGGCGTGGAGGACAGCACTTGGGGAGTGGGTGAGAAGACAGGAGACTTCAGTGGGTGGGAGGACAAGATGGGGTGACCAAGAGTGTGAGGGTGGAAGTCTGGACAGGACCCTGGGCTCATCACATGGGCCTCCGGGCAGAACCAGGGCAGCAGAGCCTCCACTCACAGCCTGGA

>chr16(-):89209094-89209313

CATCCTGGAAGCCCCGTGAGTGCCCTGGAGTCCCTCCCGCAGGCCCTTCAGACAGCAGCCCCTTCTCTCAGGTGCAAATGGATCTCATGGGCTGGAAACCACCCTTGTGCTGTGTGGAGGGCCAGGGAGAGGTGTAAGGAATGCCCCCCCACAAATGCACAGCACACGGGAGCAGCCGTCCCCAGGGCAGAGACTGGAGACGGTGGGAGAGAAGAGCTGT

>chr16(-):29527333-29527570

CGGGGACAGCAGAGATGAGGCCAGGCTTCTGCACAGAGGCAGGCATGTGCCCAAGTTCAAGGTGTGGCAGGTGGTGCTTCCACTGTCAGAGTCTGAGGCCCCCAGCCTGAGCTCTCCCACACTCCCCATGGGGTCCACAGCACCCCCAGATGGCAGGATGTGGCCTTGCAGCTCGGGGTGGGTGGGGCTGCAACCCAGACACCCTTGCCTGCCCCGGGGGCTTCCCATGCCCCCACCC

>chr16(+):85514525-85514744

CTGCCCCACTCAGTTCCCCCAGGTAAAATGGATCCTGCCTCTCACCTACTTTAAAACCCTTCCACGACTGCCCACCACCCTAGGCTGCCCTCTCACTCAGCCCCCACCATCTGGGCCTGGTCTCTCCCCACATGCAGGGCCCAGCTCGACTCCTTCACCCCGACCTTTCCATGGTCCTCTCTCCCCACGCGCACGGCCCAGCTCAACTCCTTCACCCGGG

>chr16(-):3726562-3726781

GGCTCCTCTGCCTCTCAGCAGCCGCCCATGCCCAGCTTCTCAGCATCCCGCCCAGCCAGTGCCCCAGGGAGAAGAGCCTGTGCGGGAGCAGGAAGTGGGGCTCACCTCCCCAAGCTTCCCTGTGTTGTGCTGCTGGGCGGCTCTCCAGGCCTTCAAGCTGGGGCCTTCTGAACGGTGGCCGGCTCCTCGGGTCCTGGGCAGGAGTGCTTCCCCGTGAGCT

>chr16(-):89369201-89369415

GGAGGAGCAGGGCCCTGTGGGCCCAGTGGGTGTGGAAGGTCATGTGTGGGGCAGTGTGTCTTCCAGAGGGCTCAGGAGTGTGGGGCTGGGAGCCACAGAGCCTGTGGCAGGCAGGCGTGACCAGGGACAAGTGTGTTGCCGGGGAAGACAGACCCGGAGGATCAGCGAGGCCAGGCTTCCCAGCGGTGGAGCCCCTGGACAAGGTTGTGTCCCTG

>chr17(-):82954131-82954459

CAGACGGAGGACGCCCCGCCTGCCTCAGGCCTCCTGGGTCCGTACGCTGGTGGTGACTTGGAAGAGGGCTTCTCTGGGGAGGGGCCCAAGCCCCATGGAAGTGGTTTTGCTCAGGTCGGGGGCTGCATCCCCTCACCCTCCCCAGTCTCCCTCCCAGGAGCAGCAGCTGGCAGCCAGGTCCCGGGGCGGCACCAGGAGATGGTCACTGCCGATCCTGCCTGGCTGAGACCCCGGGCTCTGGGACCAGGCTGCCCCGCTCCCTGCATCCGCCCTCACCCCGAGCTTCAGAAACCTCAGTACAAGGGGGTGATCTGCAAAGGGACCTGGGC

>chr17(-):41586347-41586643

GCAGGAGCTGGGCCCCTGTCCCTGTGCCTCTGTCTCCTCCAGGGCAGGAAAGGAAACCCAACTCCCAGTACATGGAGAACCCCACGTCCCAGGTCAGGCCCTGGCTGGGACTCAGCCTCTCACCAGCCCCACGAGGGGCTCCAGCACCCCCGACTCCTCTGGCCCCATGGGATGCTGGGCCTCTAGGGAAGAGTCGAGGGGACCATAAACTCACCAAGTGCCTCATGGTCTTGGGGTGTGACGTGGGTACCACATGCTCCTGGTGGTCTTGGAGCCCCTGGACCCCTGTGCCATTTG

>chr17(+):45658489-45658754

TCTGCTGCCAACTGCGACCTGCTACTGCCTGAGTCAAGCAGAGGCTGGGACAGTGGGTGGGTGGGACCTGGCTGGCACAGGTGAGAGGCTGTGTCTGAGGTGGAGGCTCCCAGGGGAACCTTGGGGCAGGCTGGCCCATCATGGGACCCTGGGGAGAAGGCAGGGTGGCTTTTGTACCCTTGAGCCCAGGAGCTGAGGAGGGCCAAACAGGTGAGCCAGGTAGTCTCTACCCGTAAGGCCAGGGCACCAAGGAGACAGGGAGAATT

>chr17(+):75079869-75080134

CAGGAAGGTCCCTGGCTGGGTGAGAGGCAAGGGGCTGGAAGCGGTGGGGGCGGGGATGAAGTTGGCAGGAGCTGGACCTCCCAGATCAAGGAATGGTGCTGTGGGGGCCCCTTCAGGGTCCATGTGACCCCCATCCTCACAGTGGGACCAGGGACGGACGTTCAGACTTAGAGAGGCCACCAGCCAGAGCCTCCTGAGGTCTCCCCAGCCCCTCATGGCTGGGGGAGCCAGACAGGCAGGGAGGCAGTGGCTGGAGAGTACAAAGA

>chr17(-):80952355-80952586

TTGCAGGCTCCATGCAGCCCAGCAGCCCCACCTCAGTCCAAATCCACCCCATGATGTGTGTGAGGCAGCCTGGCTCCTAGATCCAGCCAGAAAACAGGACACCAAACACTATAGGACACAGGGCACCGGGGAGGGATGATGGACTGGGCTGCCGGTGCGGCTTGGTGGGGTCCAGGACAGTGGCTGAGGGAGGAGTGAGTGTTGTCCGGGGCTCTCTGTGCTCTGTGAGGTT

>chr18(-):60741580-60741891

TCAGGCCCCAGGACCCCACTTGCCCTGCTGGGAGTGAGCCCAGTCATGAAGTCCTTGGCCTCCCAGAGCCTGTGTCTGCACTCAACAGCTGAGCCTGGAGGAGCAGAGAGCCAGAAGCGTCAGGTCCTCCGAACTGGATATTCCCCTCCACTCAGTGCCCCCAAGCTCAGAGCTTGGAGAGAGCCACACATCGGCTGCCTGTGCTGCTGGTCCCCTGGTTCAGGAGGGTCGGGTCCGCTGTGCCCAGGAGCTGCAGCCTCAGGTGCAGCACCCCATGGCTCTGGAGATGGCACCTGTGCAAACCGTGGGTGG

>chr18(-):43717400-43717636

TTCCAGGGCCTCCTCCCAGCAAAGAAGATTGTGCATCCCTGCCTCCCTCCCGGGCTCTGCAGACTGGCATACCTGCCAGGGAGCCCCTCTGCCACACGTGAGCTCTGGGGTCTGCCCGCTAGGGCAGCTCCCAGCCCTCTGGTCTCAGCGCTGCTGGCGTCTGCCATGATTTATGGCTGCACCTCCTGCACACACGCCAGGGGCAGAGGGACTCAGCACACTGTTCACAAATTAAAC

>chr18(-):56055337-56055556

AGTTGTCGTTGAGCAAGAAAGAACTCTGGGGCCATCAAAAAGAGGTTGCTTGGTGAGAGGAGTTTGCGCACACCAATGGTAGGCACCCAGGGTGAGTCGAGAAGACAGGATGTTCCAGAACATTCTCCCTTGCCTGTACTAATTCCATGGTAGGTGGGTGGGGCTGCACAAAAGCCTGGAACATTTTCCTTTTGATGTGAGCTGGAGGCAAGCTGATAGA

>chr18(-):45117249-45117514

GACCTGAGTTTGGCGGGCCCCTGGTTCTCCTGGCAGCCACGGTGCTTTTCGCAGCTTTCCGTTCTGAGAGGGGCCACTGTCTCTGGAGGTGGGGGAGGCTTTCTGCTCCTACCCAGCCATGGCCGCTTAGCAACACCTGGTGCCAGGAGAGCGTCCAAGGAGCCGCTGGTGTGTCCTGGAGCAAGCTCGCTCCCTGGGGTCCTGCCTGCACCGCCCAACCCCGAGTGCCCCAGCTTCCTGCCCTCAGATCCTAACCTTGACTCTCT

>chr18(-):46214058-46214400

CGGCTCCTTGTGGGGGAGGAAGGGCTCCCCTGGGGCCCTCTGCTCCAGGAGCACCATGCGTGTGCCCTGGCCCTATTCCCACAGGAAGAGGATGGGAGAGGTGGGGTTCTCACCTCATCCTGAAAGTAGGGCCCACTGTCCAGCAGCCTAACCCTCATCTCATTCCGGCAAGGGCAGATGTTTTAGAGAGGTGGGGCCTGTCTCCCCAGGCCTCATGACCTCAGGTGGGAGAGTCGAGTCCCTACGCCTGATGTCGCGGCCTGGAGATGCCGCTAGTTGGGTTAAGCGCCTGGCCCTCAGTTCACCTTCCAGTCCTGCTCACCTGCTCTGAGGTCATGTGCGT

>chr18(-):44706312-44706531

AGGCTGACCCCAGGGCAGAGCTCACGTGGCCCGCCTCTCTACCCCCATCGAATCAGCCCATCTCCCTCATACTCACCTGACAGGCAGGAAGCAGAGCCCCGGGGTAGGAGCAGGGGCAAGGCACGTCTCCTGGGGCCACCCCCACATCACTAGGGCAACTGGGCAGACTTCCTTGCCCCACCTACTCCTGGCCCCAGGTCACGGTCCCCACCCCTGCCAG

>chr18(-):61084244-61084463

TGTGTTTTGGAGTATGCGTTATTGGGTGTGGCAGCCACCTCCAGGCAGGTACACCCAATAACCCAATAATGCCTCTTTCCCTTACACTCTCAGGCTCTCCCACGAGCCCAGGCATGGCCACGATTAGCAAGCAAAGGTGTTGAAAGGAGTAAAGCAGAAAGTATTTTAAAGAAGGCATAACAGAGAAGACCCAGTTCTCCTAGGCCCCACTCCCACTCCA

>chr18(+):76386474-76386693

TCCTATCTTCTTTATGGCAGCGCTGTCTTTTTCAGCTCTGCTTCTGGCCACCCAAAGCCCTCCACTCAGACTGGGCAAGCTGCCCAGGGGCACAGAGGCACACACCCACCGTCATGCCCCAGAATCCCGAATGGGTGTCCGCACCAGCTCCACTCTGAACAGACAAACACTCCTTACTCTCCTATTAAAGGGCTTGGAATATTATTTCGTACACAAGCAA

>chr20(+):56322272-56322491

AGTGGTGGAGGCCATGGAAAAGCAGGAAAGGGCACACCACAGAGCCCCAGGGCAGGCAAACGGAAGGCCCTTCTGTGCAGCCAAATGTCATTCAGCCAACACCTCTGAGAAGCTTCTGTGAAACTCCTGGAAACTGCAAAGGACTTCCAGGCAGGGCAGCACAGTGAGCTCATCCTTCAGCATGCTCCCTGTGCTGCAAACACACGGCCTCAATTAAATA

>chr21(-):43005923-43006158

GGTACGGTGCCTGGCAGCGCTGAACGGGAGTGGGCGGGAGGACGTGGAGCCAGGCCAGCCTCAGTTCGGGGGTCCTGGGGCCTGTGTCCAGAGGCGAGGCCCACACAGCGGCGGGACTGGTCCTGCAGGCGCGTGGCCACAGTGGGCAGGAGTGGGGGCTTTGGGGCCCTGGCTGCCCAAAGCTTGATGCTAGCCTAGGGCCAACCAGACCCCACATTCAGGGCATACCCCACAGC

>chr22(-):16539177-16539396

CTCAGCACAGTGCCCCAGGGCTCGCCAGCCACCCAGGACCGAGACTCGTAGTGCTGGGGGGCTGCAGCTCCTTCCCATCCTTTCCTAGGGCTGCCTCTAGCCCCCATCTCTCCAAATTAGCAGGGGAGCTGGAGCCCCTAAGACCCCAGCCTCACATCATCCTCACGCCTCTGTTGGGAGCCATGCCCTGCTGCACCCGAATCTTCTGTTTCTCCCTGAC

>chr22(-):46986963-46987182

GGTGGTCTCTGCCCCACCCGGGCCCTGAGCCCACCCAGGGTCTAGTCTACCTGGCAGGGAGGGATGGGAGGCCAGCCCTGCTTTGTGGGTGCTGAGAGGATGTGGGAGAAGGACAGTGTTTAGCCTGATCCACCCTTCCTCCCATTGCCTGTGACCTGCCCTCCCCCACCCAGGGTGACCTGGCAGCCGCCCCTGCTTTTGCCTTCCTAGACCTTCCCAG

>chr22(-):40416993-40417212

ACCACCACCCCCATCTGGACACTGCAGGGCACTGGTCAGCCCCAACGCACCCACTGCTGCTCCTGCTCTGGGCCAGATCCTGTTCTGGGAGCCACAGGACCCAGACACGAAACTAGCCCTCCAAGGGCTCAGACCCGGCGAACCAGCCAGGCAGAAAACAGACCTCCTCAGAACTCTATTTTGAGGAGTTGCAGTGGAGGGGGTCTGTGGGCCCCTAAGA

>chrX(-):97883022-97883287

CTCCCGGGGTGACAGAGGCCCTTGGGGCTGCAGAAGCCCCTGCAATGGCAGGGGCTCCCAAAGTGGCAGAAGCTCCCAGAGAAGCGGAGACTTCCAGGGCAGCGGTGCCTCCTGGGACAGTGGTGCCTACCGAAGCGGCAGCACCCACTGAGGTGACCGAGGGTCCTGGGGTAGCAGCACCTACCAAGGTAGCTGAAGCTCCCGGGGTGGCATCGCCTACCGAGGCAGCTGAGGCTCCTGTGCCCGCAACGCCTACTGGGGCTGCA

>chrX(+):8621551-8621881

CACAGGAGAGTGGGCGCTGGGCTGTGCTCTGAGACCGTTCCTTGTCTTGCTTGGCTTTTTCTTTGGCCAAGTGGAGACAGCACCAGGTGGCCAGACTGTGTTGGGCCTGGTCGTGTGCTTGATCTGCTGTACTGGGGATGTCGCCTCGTACCCCTGTGCCCAGCTCCTTGCGGCTGGGTGGGTCTCCTTCACCCTCCTGTCCTTGCTGGCTCAGTGTTTGTGTGCCTGGCCAATATAATGTGTGGCAGGTTCCTCACATCCTTCTTGGGCCAGGCGGGGTGCCTTCGCTAGGGCACTGCAGCCTCAGAAGGTGGTAGCACCTGCCCCCATG

>chrX(-):127052964-127053183

CAGAGGTGAGGGTAGGGGGCTGCGGAATGTGCGCCCTCTGCCTCTCGGGGTTGCCCTGGGTCTCTGGCTGCCACTGCGCTGCAGAGCATGGCCTTTAGGCTGGGTTGAGGTGCGCTGCGCGGGTCCTGCCCCTGGGTAGCTCCAGGCAGCGCAGAACCTGTAAAGTTAAACAAAGCCCTGCGGCGCGCGGAAACCACTCACTGCTTCCTGCTCCTCTGAG

>chrX(+):129481471-129481690

CTTGCCTCTGATTTCCCAGGAACCTTTAGCATTTTCTGATTTCTGTGTCTGTGCTTGGAACTGGGAGTGGATGTGTAGGTCTGCTCTCAGGAAGAACAGATATCCAGCTTGTGGTTTAGCTTCCTTTTTCTGGCTAAGAGATCCTTGGACAACCTCAGGGGCTAAGTCCCTTTGCCTGGGTATACCTGGGGACTCCAGCAAGCCCCTTCTAGGCACCCCA

>chrX(+):110089-110400

TGGGGCCGTGCAGCTGCTTGGCTGAGCTCTTCTTGCCGGAGCCACTGCCTGCGCCTCCTGCCCAGGGATTCTCGGGCCTTCTGTCCCCGGGCAACAGCAGGACAGCTCAGACCTTGTTCCCAGCCCAGGACAGCTGTGCTGCCTAGCCTGGCCCTCCCCGCCTGGGGAGTGACTCTCCCGCTCCCCACACACTGACTCAGCACACAGACGCCCCTCTTCACCTGTCCTGGTCACACAGACACTGTCCGGCGTCCCCACGGGGTCCTTCGGGTGGACGCACAGACCCTACACGGCACCACGCATGGCTGAAAG

>chrX(+):7504256-7504475

TGTATCCTGTGGGAGCAGGAGGGGTCGGAGAAGATGCCAGTGTCCCCTCCTATCCTTCGTGGCCCGTCCAGCCCGTCCCTGGGGCTGGCTGCAGAACTCACAGCTGCCTCGCACACCTGAATGGCCCCTGCAGGATCCTTGCGGTGTCCCTGGGACAGAGTTCAGCCCTGCCCCTGTGTGCCCCAGAGACCTGCGTGGCCCGGAGGACCACGGGTGTCAC

>chrX(+):2575220-2575439

ACCACCACTCCAGTGCTTCCCAGGTGCCTGCTCTCCGCAACTCTCTGGATGGCTGGGAAGCGGGGCACCCAGGAAAGCAAGCAGTTCCAAGAGGAGGAAGGGTCCCTGCTGGGCGGGTGTACACAGGTGGGCTCCTGGCGCCCACCTGCTGCCTGAGTCTCAGCCTCAGAGGAGCTCAGAGGATAGATGAGATGCACCCACTGCTGGGAACTGGGAGAAA

>chrX(-):38485130-38485391

TCAGTGTGGAGGTGCTGGGTCTCAGAGCTTCAGTCACTACCTCAGACACTCCCACAATAGCCGGGAAGCCAGCCCCTGAGGTGGGAAGGAGGGCTTCCTGCCAGCCCCTTCCCATAAGGCAAACAGCTTTGCCCATGCCCTGAGCAGCAGCGCCCAGCTCCCTCCTTCATGCCACACCCTCCAGGCTCACCTGAGGCACAGTGGTTGGGACGGGCCTGGGTTTCACCTGTTTTGGCTGCAGGTTCCATTTGTCAGCCTGGAT

>chrX(-):115083131-115083381

CCCGTGGCTTCCAGGAGCAGCAGGAAGCACTGGGCTTGAGGACCAGGGGCCCTACCCCACCTGGAGGGAGATGGCACTCCTGCACTGGTGTTTCTCTTTGGAGAGTGCCCACAGCCTGCTTTGGCTGTGACACAGTAGACTAGGAGGCCAGCAAGGGACCAAGTCACTCCCTGTATGTGGGGGGCGGGCGCAGGGTCTCCCCTGAGGGGTGAGATCCCTCATTGTCCCCAGCCTCACAGCAGAATCACACT

>chrX(+):1009488-1009753

TATGCCCTGTCACCCCCAATCATGGAGACCCATGCCTTCTGTGAGGCACCCTGACCACTCTCATCCCCGTCACTAGGACCAGGCATGTGGTGTCCCCCCTCCCAGGGTACCAGACCTCCAGTCCGTAGTCCCCAAGAGTAGACTCCTGCCCCACTGCACCATCCATAGGCAACCAGTAACTACCCTACCATCACCAGTAACTTCTCTGCCATCACCAGTAACTACCTCACATCACCAGTAACTACCCCCCATCACCAGTAACTATG

>chrX(+):7035409-7035628

ACAAGGAGGGAGGCTGAGTGAGCACATTCCCAACAGCATGCCTAGATAGACCACCTGATCTCACAGAGAAGGGGTGTTAGAAGGAAACTCTACTGGAGGTCAGGAAGGTCATTGCAGGCCAGAGTGGTTGAGAATGCTTCACCAAGACAGAACTATCCCTGAAAGGGAGGTTATGTTTGAATCTGGGTGAGTGGTCTAACGTTGGGAGGATTCTTTATAA

>chrY(+):27517507-27517801

TCCTGCTGCCAGTCATCCCGAATGGGCAGTTACAAAGATATGGCTCTGGCCTAGAAGCCGGAGATGCCCTGGATGATGGCCCCTGTGCCCTCCAGGCCAGGCAGACACTTCTGACAAAGCTTCTGCCTCAGCCATGGGCAGGGCATGTGGCCTGGGGCATTCACGGAGCCCAGCTCCCTGTGAAGGACCTCCAGCGACTCTGTGGCCGGCTGGGGCATGCTGGGGCCAGGGCAGGCTGTGTTCACTGGTCCTCCACCTGCCGCTCCATGTCGGCTTTCTCCTCAACCACCACCTA

>chrY(+):9842107-9842326

GCAGTGGCATGTGGCTGGCCCAAAAGCACGGTCCAGCTATGTCCACCTGATTCCAGGCATCACCATCAACTCTGGGCTGTGAGGCTGGGATCAGGCACCCCAGAGCTGCTTGCCTGTGGCCGGGCTGCTCTCCCCCTCTACGCCCAAGCACCACTAGGCACCGCGCTGCACTTTCTGCCGACCTCCCACAGTGTCCCCCTGTCGCCGGTGGCTAGACCAC
