# Supplementary material for: Comparative analysis of genome tiling array data reveals many novel primate-specific functional RNAs in human
Source: BMC Evol Biol. 2007 Feb 8;7(Suppl 1):S14. doi: 10.1186/1471-2148-7-S1-S14 (PMC1796608; doi:10.1186/1471-2148-7-S1-S14)
Supplement: Additional file 3 — UCSC genome browser screen shots of these 3 candidate sequences as shown in Figure 2. These screenshots show that these sequences are either absent or less conserved in the mouse and rat genomes, thus are primate-specific. [file 1471-2148-7-S1-S14-S3.pdf]

Home Genomes Blat Tables Gene Sorter PCR DNA Convert PDF/PS Help

UCSC Genome Browser on Human Mar. 2006 Assembly

move <<< << < > >> >>> zoom in 1.5x 3x 10x base

zoom out 1.5x 3x 10x

position/search chr2:10,311,401-10,311,620 jump clear size 220 bp.

configure

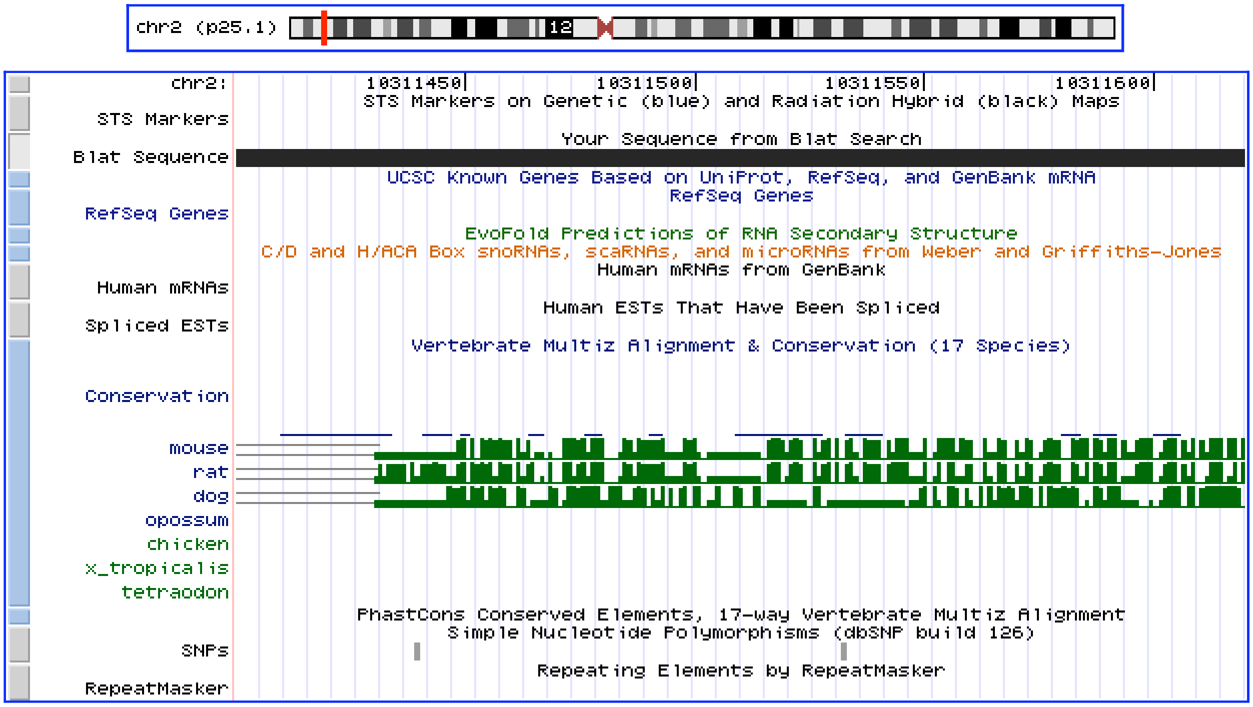

move start < 2.0 >

Click on a feature for details. Click on base position to zoom in around cursor. Click on left mini-buttons for track-specific options.

move end < 2.0 >

default tracks hide all custom tracks configure refresh

Use drop down controls below and press refresh to alter tracks displayed.
Tracks with lots of items will automatically be displayed in more compact modes.

Mapping and Sequencing Tracks

Base Position	Chromosome Band	STS Markers	FISH Clones	Recomb Rate
dense	hide	dense	hide	hide
Map Contigs	Assembly	Gap	Coverage	BAC End Pairs
hide	hide	hide	hide	hide
Fosmid End Pairs	GC Percent	Short Match	Restr Enzymes	Blat Sequence
hide	hide	hide	hide	dense

Genes and Gene Prediction Tracks

Known Genes	RefSeq Genes	Other RefSeq	MGC Genes	Ensembl Genes
pack	dense	hide	hide	hide
N-SCAN	SGP Genes	Geneid Genes	Genscan Genes	Superfamily
hide	hide	hide	hide	hide
EvoFold	sno/miRNA			
full	full			

mRNA and EST Tracks

Human mRNAs	Spliced ESTs	Human ESTs	Other mRNAs	Other ESTs
dense	dense	hide	hide	hide
H-Inv				
hide				

Expression and Regulation

Allen Brain	GNF Atlas 2	GNF Ratio	Affy U133	Affy GNF1H
hide	hide	hide	hide	hide
Affy U133Plus2	Affy U95	CpG Islands	FirstEF	Reg Potential 7 species
hide	hide	hide	hide	hide

Comparative Genomics

Conservation	Most Conserved	Fugu Chain	Fugu Net	Tetraodon Chain
full	full	hide	hide	hide
Tetraodon Net	Tetraodon Ecores	Zebrafish chain	Zebrafish Net	X. tropicalis Chain
hide	hide	hide	hide	hide
X. tropicalis Net	Chicken Chain	Chicken Net	Cow Chain	Cow Net
hide	hide	hide	hide	hide
Dog Chain	Dog Net	Rat Chain	Rat Net	Mouse Chain
hide	hide	hide	hide	hide
Mouse Net	Rhesus Chain	Rhesus Net	Chimp Chain	Chimp Net
hide	hide	hide	hide	hide

Variation and Repeats

SNPs	RepeatMasker	Simple Repeats	Self Chain
dense	dense	hide	hide

refresh

Home Genomes Blat Tables Gene Sorter PCR DNA Convert PDF/PS Help

UCSC Genome Browser on Human Mar. 2006 Assembly

move <<< << < > >> >>> zoom in 1.5x 3x 10x base

zoom out 1.5x 3x 10x

position/search chr11:113,391,068-113,391,287 jump clear size 220 bp.

configure

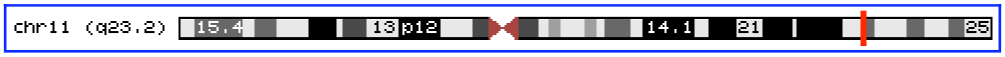

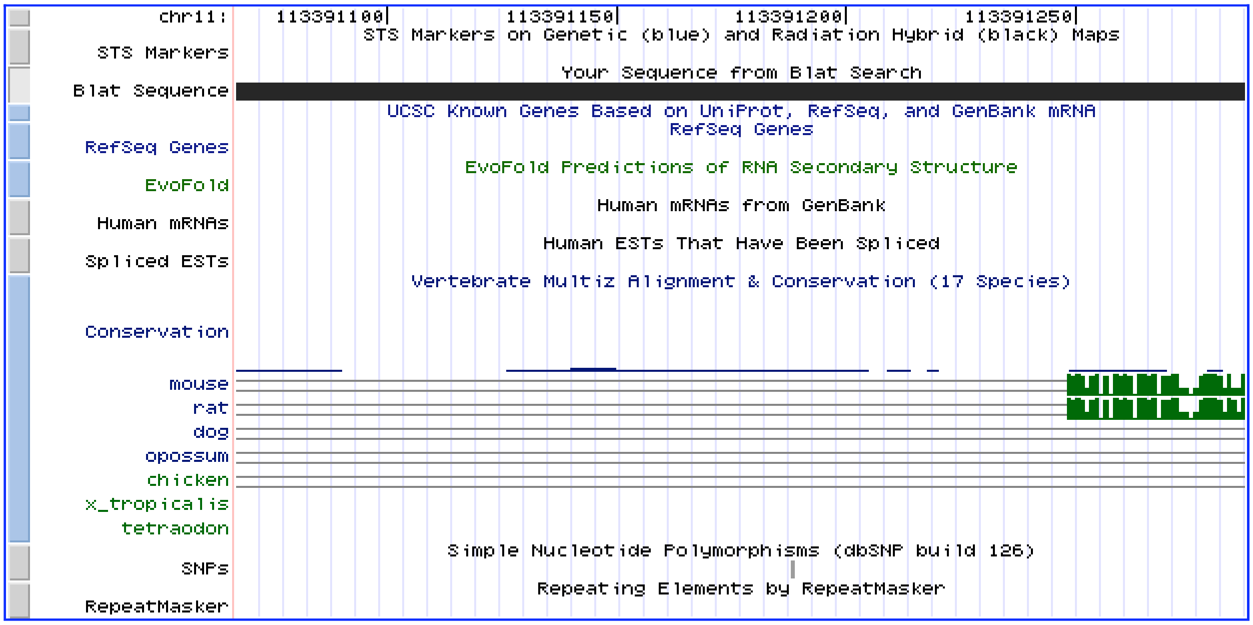

move start < 2.0 > Click on a feature for details. Click on base position to zoom in around cursor. Click on left mini-buttons for track-specific options. move end < 2.0 >

default tracks hide all custom tracks configure refresh

Use drop down controls below and press refresh to alter tracks displayed.
Tracks with lots of items will automatically be displayed in more compact modes.

Mapping and Sequencing Tracks

Base Position	Chromosome Band	STS Markers	FISH Clones	Recomb Rate
dense	hide	dense	hide	hide
Map Contigs	Assembly	Gap	Coverage	BAC End Pairs
hide	hide	hide	hide	hide
Fosmid End Pairs	GC Percent	Short Match	Restr Enzymes	Blat Sequence
hide	hide	hide	hide	dense

Genes and Gene Prediction Tracks

Known Genes	RefSeq Genes	Other RefSeq	MGC Genes	Ensembl Genes
pack	dense	hide	hide	hide

N-SCAN	SGP Genes	Geneid Genes	Genscan Genes	Superfamily
hide	hide	hide	hide	hide
EvoFold	sno/miRNA			
dense	hide			

mRNA and EST Tracks

Human mRNAs	Spliced ESTs	Human ESTs	Other mRNAs	Other ESTs
dense	dense	hide	hide	hide
H-Inv				
hide				

Expression and Regulation

Allen Brain	GNF Atlas 2	GNF Ratio	Affy U133	Affy GNF1H
hide	hide	hide	hide	hide
Affy U133Plus2	Affy U95	CpG Islands	FirstEF	Reg Potential 7 species
hide	hide	hide	hide	hide

Comparative Genomics

Conservation	Most Conserved	Fugu Chain	Fugu Net	Tetraodon Chain
full	hide	hide	hide	hide
Tetraodon Net	Tetraodon Ecores	Zebrafish chain	Zebrafish Net	X. tropicalis Chain
hide	hide	hide	hide	hide
X. tropicalis Net	Chicken Chain	Chicken Net	Cow Chain	Cow Net
hide	hide	hide	hide	hide
Dog Chain	Dog Net	Rat Chain	Rat Net	Mouse Chain
hide	hide	hide	hide	hide
Mouse Net	Rhesus Chain	Rhesus Net	Chimp Chain	Chimp Net
hide	hide	hide	hide	hide

Variation and Repeats

SNPs	RepeatMasker	Simple Repeats	Self Chain
dense	dense	hide	hide

refresh

Home Genomes Blat Tables Gene Sorter PCR DNA Convert PDF/PS Help

UCSC Genome Browser on Human Mar. 2006 Assembly

move [<<<] [<<] [<] [>] [>>] [>>>] zoom in [1.5x] [3x] [10x] [base]

zoom out [1.5x] [3x] [10x]

position/search [chr7:56,839,906-56,840,127] [jump] [clear] size 222 bp.

[configure]

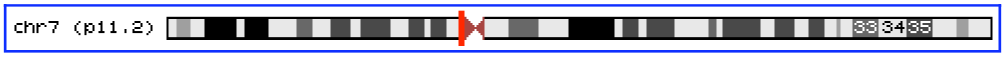

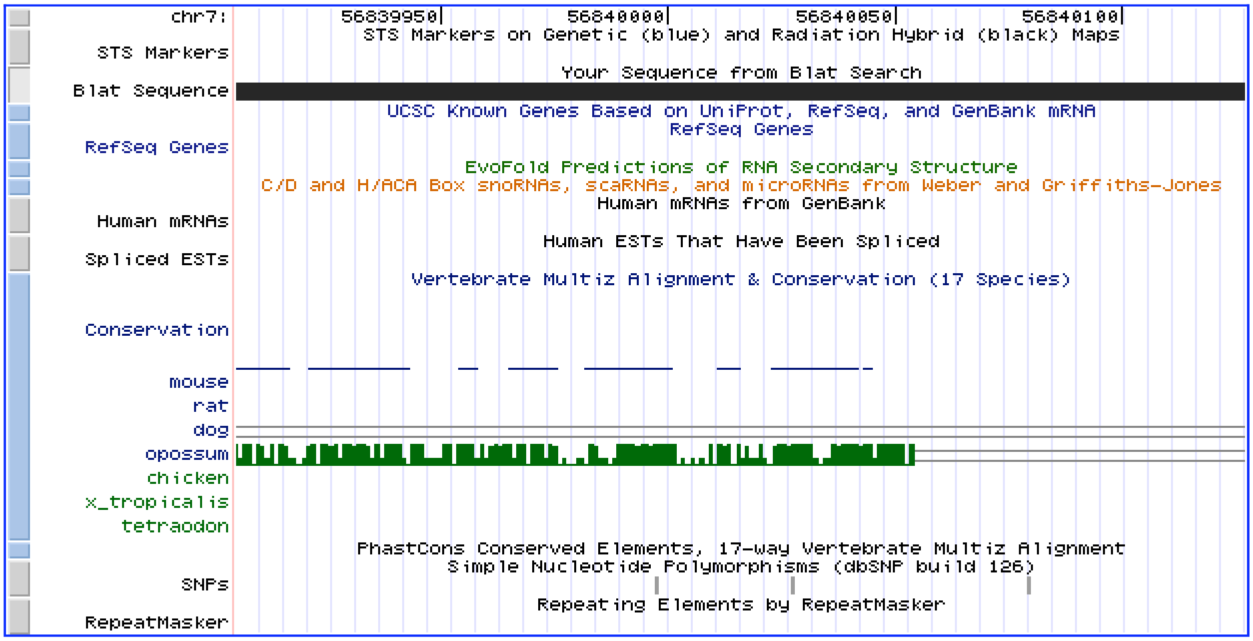

move start [<] [2.0] [>]

Click on a feature for details. Click on base position to zoom in around cursor. Click on left mini-buttons for track-specific options.

move end [<] [2.0] [>]

[default tracks] [hide all] [custom tracks] [configure] [refresh]

Use drop down controls below and press refresh to alter tracks displayed.
Tracks with lots of items will automatically be displayed in more compact modes.

Mapping and Sequencing Tracks

Base Position	Chromosome Band	STS Markers	FISH Clones	Recomb Rate
dense	hide	dense	hide	hide
Map Contigs	Assembly	Gap	Coverage	BAC End Pairs
hide	hide	hide	hide	hide
Fosmid End Pairs	GC Percent	Short Match	Restr Enzymes	Blat Sequence
hide	hide	hide	hide	dense

Genes and Gene Prediction Tracks

Known Genes	RefSeq Genes	Other RefSeq	MGC Genes	Ensembl Genes
pack	dense	hide	hide	hide
N-SCAN	SGP Genes	Geneid Genes	Genscan Genes	Superfamily
hide	hide	hide	hide	hide
EvoFold	sno/miRNA			
full	full			

mRNA and EST Tracks

Human mRNAs	Spliced ESTs	Human ESTs	Other mRNAs	Other ESTs
dense	dense	hide	hide	hide
H-Inv				
hide				

Expression and Regulation

Allen Brain	GNF Atlas 2	GNF Ratio	Affy U133	Affy GNF1H
hide	hide	hide	hide	hide
Affy U133Plus2	Affy U95	CpG Islands	FirstEF	Reg Potential 7 species
hide	hide	hide	hide	hide

Comparative Genomics

Conservation	Most Conserved	Fugu Chain	Fugu Net	Tetraodon Chain
full	full	hide	hide	hide
Tetraodon Net	Tetraodon Ecores	Zebrafish chain	Zebrafish Net	X. tropicalis Chain
hide	hide	hide	hide	hide
X. tropicalis Net	Chicken Chain	Chicken Net	Cow Chain	Cow Net
hide	hide	hide	hide	hide
Dog Chain	Dog Net	Rat Chain	Rat Net	Mouse Chain
hide	hide	hide	hide	hide
Mouse Net	Rhesus Chain	Rhesus Net	Chimp Chain	Chimp Net
hide	hide	hide	hide	hide

Variation and Repeats

SNPs	RepeatMasker	Simple Repeats	Self Chain
dense	dense	hide	hide

refresh
